# Identifying oscillatory brain networks with hidden Gaussian graphical spectral models of MEEG

**DOI:** 10.1038/s41598-023-38513-y

**Published:** 2023-07-15

**Authors:** Deirel Paz-Linares, Eduardo Gonzalez-Moreira, Ariosky Areces-Gonzalez, Ying Wang, Min Li, Eduardo Martinez-Montes, Jorge Bosch-Bayard, Maria L. Bringas-Vega, Mitchell Valdes-Sosa, Pedro A. Valdes-Sosa

**Affiliations:** 1 The Clinical Hospital of Chengdu Brain Science Institute, MOE Key Lab for Neuroinformation, School of Life Science and Technology, University of Electronic Science and Technology of China, Chengdu, China; 2 Department of Neuroinformatics, Cuban Neuroscience Center, Havana, Cuba; 3 School of Electrical Engineering, Central University “Marta Abreu” of Las Villas, Santa Clara, Cuba; 4 School of Technical Sciences, University of Pinar del Río “Hermanos Saiz Montes de Oca”, Pinar del Rio, Cuba; 5 McGill Centre for Integrative Neurosciences MCIN, Ludmer Centre for Mental Health, Montreal Neurological Institute, McGill University, Montreal, Canada; 6 Center for Biomedical Imaging and Neuromodulation, Nathan Kline Institute for Psychiatric Research, Orangeburg, NY USA

**Keywords:** Network models, Statistics

## Abstract

Identifying the functional networks underpinning indirectly observed processes poses an inverse problem for neurosciences or other fields. A solution of such inverse problems estimates as a first step the activity emerging within functional networks from EEG or MEG data. These EEG or MEG estimates are a direct reflection of functional brain network activity with a temporal resolution that no other in vivo neuroimage may provide. A second step estimating functional connectivity from such activity pseudodata unveil the oscillatory brain networks that strongly correlate with all cognition and behavior. Simulations of such MEG or EEG inverse problem also reveal estimation errors of the functional connectivity determined by any of the state-of-the-art inverse solutions. We disclose a significant cause of estimation errors originating from misspecification of the functional network model incorporated into either inverse solution steps. We introduce the Bayesian identification of a Hidden Gaussian Graphical Spectral (HIGGS) model specifying such oscillatory brain networks model. In human EEG alpha rhythm simulations, the estimation errors measured as ROC performance do not surpass 2% in our HIGGS inverse solution and reach 20% in state-of-the-art methods. Macaque simultaneous EEG/ECoG recordings provide experimental confirmation for our results with 1/3 times larger congruence according to Riemannian distances than state-of-the-art methods.

## Introduction

In vivo identification of functional brain networks would greatly benefit from the inverse solutions for MEG/EEG electromagnetic observation modalities^1,2^. A direct relationship to the activity of postsynaptic potentials (PSPs) is attributed to the latent brain variables generating the MEG/EEG^3–5^. Where the actual latent brain variables are local currents, vector process ${\varvec{\iota}}\left(t\right)$ in time-domain $t$ with components representing locally summated PSP myriads within a spatial scale of millimeters^6^. See Supplementary Information SI I. Notation for variables and mathematical operations and SI II. Nomenclature for theoretical quantities.

MEG/EEG observations ${\varvec{v}}\left(t\right)$ with millisecond temporal resolution are due to these latent brain variables (or activity) ${\varvec{\iota}}\left(t\right)$ converted according to a linear and stationary forward-operator ${\mathbf{L}}_{{\varvec{v}}{\varvec{\iota}}}$ or Lead Field summarizing an *Electromagnetic Forward Model* (EFM)^7–9^. An inverse solution from data ${\varvec{v}}\left(t\right)$ provides estimates $\widehat{{\varvec{\iota}}}\left(t\right)$ for this latent brain activity within a natural timescale^10^. Estimates $\widehat{{\varvec{\iota}}}\left(t\right)$ could therefore help identify functional brain networks in association with the actual PSP activity and within very small timescales, avoiding time-domain distortions^11,12^.

The target of all identification is estimating functional connectivity within a given functional network model interpreted as the set of parameters ${{\varvec{\Theta}}}_{{\varvec{\iota}}{\varvec{\iota}}}$ governing coactivation in ${\varvec{\iota}}\left(t\right)$, which is without loss of generality represented by a multivariate probability $p\left(\left.{\varvec{\iota}}\left(t\right)\right\vert {{\varvec{\Theta}}}_{{\varvec{\iota}}{\varvec{\iota}}}\right)$^13,14^. Therefore, inverse solutions estimating $\widehat{{\varvec{\iota}}}\left(t\right)$ are only a first step to subsequently estimate latent functional connectivity ${{\varvec{\Theta}}}_{{\varvec{\iota}}{\varvec{\iota}}}$ (a **multistep** procedure). Estimation ${\widehat{{\varvec{\Theta}}}}_{{\varvec{\iota}}{\varvec{\iota}}}$ commonly requires a second-step inverse solution from such brain activity pseudodata $\widehat{{\varvec{\iota}}}\left(t\right)$ given the functional network model $p\left(\left.\widehat{{\varvec{\iota}}}\left(t\right)\right\vert {{\varvec{\Theta}}}_{{\varvec{\iota}}{\varvec{\iota}}}\right)$^15–17^.

**Figure 1:**
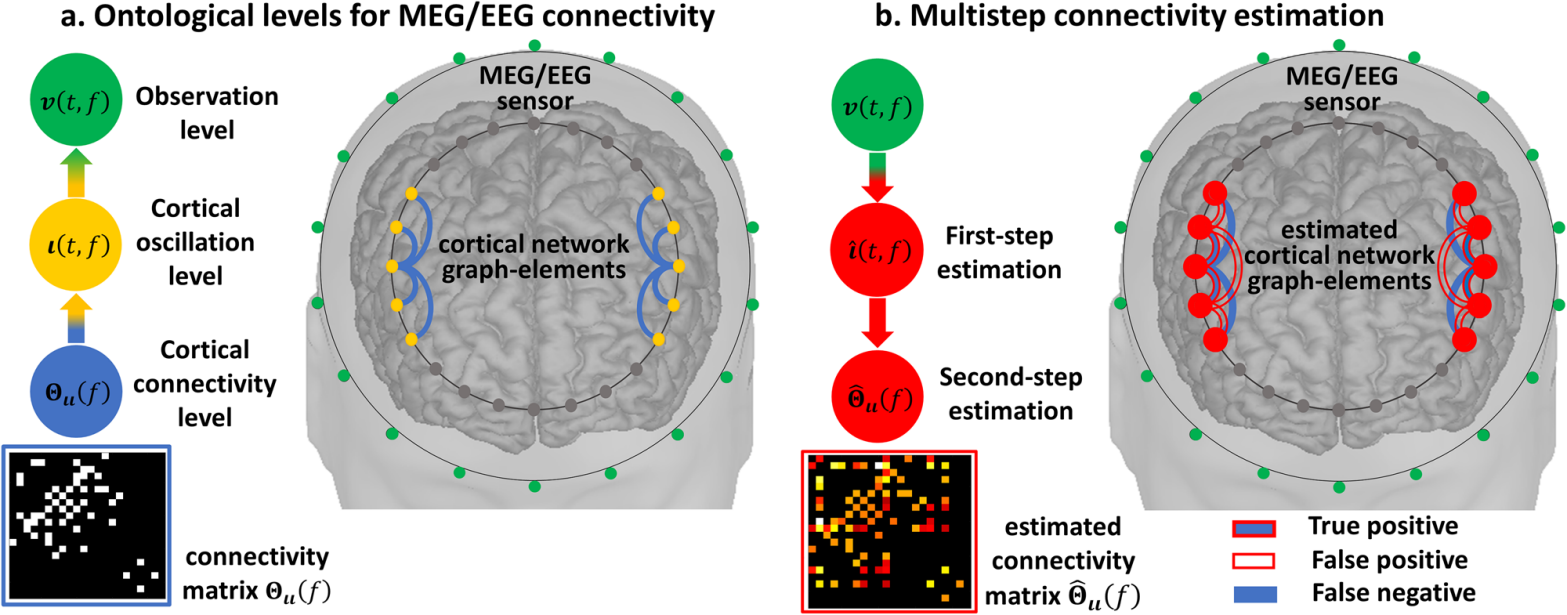
Illustration of the ontological levels involved and basic multistep identification of connectivity. (**a**) Generation by the electromagnetic forward model of the periodic components or oscillations in MEG/EEG observations ${\varvec{v}}\left(t,f\right)$ from brain oscillations ${\varvec{\iota}}\left(t,f\right)$ emerging from functional graph-elements or functional connectivity ${{\varvec{\Theta}}}_{{\varvec{\iota}}{\varvec{\iota}}}\left(f\right)$ defining a cortical oscillatory network at a given frequency $f$. (**b**) Functional connectivity distortions due to multistep inverse-solutions of the optimal cortical oscillations $\widehat{{\varvec{\iota}}}\left(t,f\right)$ explaining the observations and functional connectivity ${\widehat{{\varvec{\Theta}}}}_{{\varvec{\iota}}{\varvec{\iota}}}\left(f\right)$, from pseudodata $\widehat{{\varvec{\iota}}}\left(t,f\right)$. Even with with perfect spatial localization estimates $\widehat{{\varvec{\iota}}}\left(t,f\right)$ by any first step inverse-solution produce however false positives and false negatives functional graph-elements in the second-step inverse-solution ${\widehat{{\varvec{\Theta}}}}_{{\varvec{\iota}}{\varvec{\iota}}}\left(f\right)$.

Functional brain networks synchronized at specific frequencies or bands of frequency (Fig. 1a) are thought to underpin the periodic components or oscillations observed in the MEG/EEG^18^. These oscillations are a spectral vector process ${\varvec{v}}\left(t,f\right)$ at a frequency $f$ obtained from the narrow band filtered, or alternatively Hilbert transform of the narrow band filtered vector process ${\varvec{v}}\left(t\right)$. Oscillations ${\varvec{v}}\left(t,f\right)$ then reflect analogous latent brain oscillations ${\varvec{\iota}}\left(t,f\right)$^19–21^ according to the forward operator ${\mathbf{L}}_{{\varvec{v}}{\varvec{\iota}}}$ of the EFM spectral equivalent. These so-called oscillatory brain networks are described by frequency-specific functional connectivity ${{\varvec{\Theta}}}_{{\varvec{\iota}}{\varvec{\iota}}}\left(f\right)$, which produces brain oscillations ${\varvec{\iota}}\left(t,f\right)$ even within a millisecond timescale $t$^22^. Specific oscillatory network patterns exhibit a transient behavior which may be consider stationary in a timescale of approximately seconds^23–27^.

Important properties of oscillatory brain networks are characterized by *Gaussian Graphical Spectral* (GGS) models. A GGS model defines the multivariate probabilities governing such latent brain oscillations ${\varvec{\iota}}\left(t,f\right)$ under specific stationarity and mixing conditions in the frequency domain^28^. Details on such conditions and limits for the applicability of GGS models are provided in Materials and methods section "Gaussian graphical spectral (GGS) model and MAP1 inverse solution with Hermitian graphical LASSO (hgLASSO)". The GGS model probabilities $p\left(\left.{\varvec{\iota}}\left(t,f\right)\right\vert {{\varvec{\Theta}}}_{{\varvec{\iota}}{\varvec{\iota}}}\left(f\right)\right)$ are based upon a frequency-specific Hermitian precision-matrix ${{\varvec{\Theta}}}_{{\varvec{\iota}}{\varvec{\iota}}}\left(f\right)$ or inverse of the covariance-matrix denominated cross-spectrum ${{\varvec{\Sigma}}}_{{\varvec{\iota}}{\varvec{\iota}}}\left(f\right)$ (${{\varvec{\Theta}}}_{{\varvec{\iota}}{\varvec{\iota}}}\left(f\right)={{\varvec{\Sigma}}}_{{\varvec{\iota}}{\varvec{\iota}}}^{-1}\left(f\right)$): where the Hermitian precision-matrix ${{\varvec{\Theta}}}_{{\varvec{\iota}}{\varvec{\iota}}}\left(f\right)$ off-diagonal entries define functional connectivity parameters as Hermitian graph elements, known as undirected graph elements within similar real-valued functional network models^29–37^. Identifying these networks may then be regarded as equivalent to estimating the Hermitian precision matrix ${\widehat{{\varvec{\Theta}}}}_{{\varvec{\iota}}{\varvec{\iota}}}\left(f\right)$, which would in turn provide all functional connectivity proxies under the same GGS model assumption^21,26,38^.

The identification of oscillatory brain networks is potentially viable with **multistep** MEG/EEG inverse solutions (Fig. 1b), obtaining functional connectivity proxies ${\widehat{{\varvec{\Theta}}}}_{{\varvec{\iota}}{\varvec{\iota}}}\left(f\right)$ from brain-oscillation pseudodata $\widehat{{\varvec{\iota}}}\left(f,t\right)$ with strong correlation to cognition and behavior in normal or abnormal brain conditions^20,21,23–27,39–51^.

State-of-the-art practice targets the precision matrix ${\widehat{{\varvec{\Theta}}}}_{{\varvec{\iota}}{\varvec{\iota}}}\left(f\right)$ by a second-step inverse solution^41,52,53^. In addition to this second-step inverse solution, postprocessing may also target corrections to spatial distortions or leakage in ${\widehat{{\varvec{\Theta}}}}_{{\varvec{\iota}}{\varvec{\iota}}}\left(f\right)$^38,41,42,54^. Leakage emerges in brain-oscillation pseudodata $\widehat{{\varvec{\iota}}}\left(f,t\right)$ due to the first-step inverse solutions^55,56^, which is regarded as a major cause of distortions in precision-matrix estimates ${\widehat{{\varvec{\Theta}}}}_{{\varvec{\iota}}{\varvec{\iota}}}\left(f\right)$ or any functional connectivity proxy.

Nevertheless, either inverse solutions (first step for $\widehat{{\varvec{\iota}}}\left(f,t\right)$ and second step for ${\widehat{{\varvec{\Theta}}}}_{{\varvec{\iota}}{\varvec{\iota}}}\left(f\right)$) add estimation errors to these distortions in ${\widehat{{\varvec{\Theta}}}}_{{\varvec{\iota}}{\varvec{\iota}}}\left(f\right)$, which stems from miss specifying the actual GGS model $p\left(\left.{\varvec{\iota}}\left(t,f\right)\right\vert {{\varvec{\Theta}}}_{{\varvec{\iota}}{\varvec{\iota}}}\left(f\right)\right)$ governing the latent brain oscillations ${\varvec{\iota}}\left(t,f\right)$. It is therefore the purpose of this manuscript to explain such estimation errors and theoretically correct estimation in terms of Bayesian inverse solutions.

### Bayesian inverse solution as a maximum a posteriori probability (MAP)

An inverse solution (Eq. 1) estimating the latent variables (categories $\mathcal{X}$ and $\mathcal{Z}$) from their data (category $\mathcal{Y}$) is without loss of generality a Bayesian *maximum *a posteriori* probability* (MAP)^57^. A MAP $\widehat{\mathcal{X}}$ is the optimum value or estimate for an a posteriori probability $p\left(\left.\mathcal{X}\right\vert \mathcal{Y}\right)$ computed for a given likelihood $p\left(\left.\mathcal{Y}\right\vert \mathcal{X}\right)$ explaining $\mathcal{Y}$ upon $\mathcal{X}$ and positing an a priori probability $p\left(\mathcal{X}\right)$ upon $\mathcal{X}$. The nominal Bayesian procedure^58^ is a *first-type MAP* (MAP1) involving only a given first-type likelihood $p\left(\left.\mathcal{Y}\right\vert \mathcal{X}\right)$. The full Bayesian procedure^59^ is a *second-type MAP* (MAP2) involving a second-type likelihood $p\left(\left.\mathcal{Y}\right\vert \mathcal{X}\right)$, which must be determined by marginalizing a given first-type likelihood $p\left(\left.\mathcal{Y}\right\vert \mathcal{Z}\right)$ explaining $\mathcal{Y}$ upon (parameters) $\mathcal{Z}$. An a priori probability $p\left(\left.\mathcal{Z}\right\vert \mathcal{X}\right)$ explains these parameters upon (hyperparameters) $\mathcal{X}$.

1 $$\begin{array}{l}\widehat{\mathcal{X}}={argmax}_{\mathcal{X}}\left\{p\left(\left.{\mathcal{X}}\right\vert {\mathcal{Y}}\right)\right\} \\ p\left(\left.{\mathcal{X}}\right\vert {\mathcal{Y}} \right)=p\left(\left.{\mathcal{Y}}\right\vert {\mathcal{X}} \right)p\left({\mathcal{X}}\right)/p\left({\mathcal{Y}}\right)\\ p\left(\left.{\mathcal{Y}}\right\vert {\mathcal{X}} \right)=\int p\left(\left.{\mathcal{Y}} \right\vert {\mathcal{Z}} \right)p\left(\left.{\mathcal{Z}} \right\vert {\mathcal{X}} \right) d { \mathcal{Z}} \end{array}$$

where such likelihood model $p\left(\left.\mathcal{Y}\right\vert {\mathcal{X}} \right)$ (first-type or second-type) has an ill-conditioned nature and estimating $\widehat{\mathcal{X}}$ (Eq. 1) poses an inverse problem, standing for no unique entries for ${\mathcal{X}}$ explain a given data in ${\mathcal{Y}}$^60^. Then, an MAP posits an a priori probability $p\left({\mathcal{X}}\right)$ regularizing such ill conditioning and preferably pursuing sparse selection of those variables within ${\mathcal{X}}$, actually explaining the given data in ${\mathcal{Y}}$. The term regularization by sparse variable selection is valid for the type of ill conditioning, such as that dealt with inverse solutions (Eq. 1) in the first step for latent vectors ${\varvec{\iota}}\left(t,f\right)$^61,62^ and in the second step for latent precision matrices ${{\varvec{\Theta}}}_{{\varvec{\iota}}{\varvec{\iota}}}\left(f\right)$^35,63^.

### First step MAP1 inverse solution for brain oscillations and estimation errors

The first step MAP1 (Eq. 1) is for the brain oscillations (Fig. 1b) ${\varvec{\iota}}\left(f,t\right)$ upon the MEG/EEG oscillations (data) ${\varvec{v}}\left(t,f\right)$ (Eq. 2): where the EFM spectral equivalent yields a Gaussian first-type likelihood $p\left(\left.{\varvec{v}}\left(t,f\right)\right\vert {\varvec{\iota}}\left(f,t\right)\right)$. Here, likelihood ill-conditioning is most severe in the sense of Hadamard^64^, caused by a large number of variables describing latent brain oscillations ${\varvec{\iota}}\left(f,t\right)$ compared to the available data ${\varvec{v}}\left(t,f\right)$^10^.

An a priori probability $p\left(\left.{\varvec{\iota}}\left(f,t\right)\right\vert {\varvec{\Lambda}}\left(f\right)\right)$ (Eq. 2) regularizes this ill-conditioning also incorporating some information ${\varvec{\Lambda}}\left(f\right)$, preferably with a Gaussian model upon covariance-matrix ${\varvec{\Lambda}}\left(f\right)$ (sometimes a diagonal matrix) somehow specified from MEG/EEG data or structural information^62,65–71^.

State-of-the-art practice choses this Gaussian leading to the type of linear and stationary inverse solution to avoid introducing time-domain distortions in brain oscillations pseudodata $\widehat{{\varvec{\iota}}}\left(f,t\right)$, which may further affect functional connectivity estimates ${\widehat{{\varvec{\Theta}}}}_{{\varvec{\iota}}{\varvec{\iota}}}\left(f\right)$^20,38,41,72^. The most common examples are *Exact Low Resolution Electromagnetic Tomographic Analysis* (**eLORETA**)^56^ and Linearly Constrained Minimum Variance (**LCMV**)^55^. This MAP1 may include, in addition to ${\varvec{\Lambda}}\left(f\right)$, a sensor noise covariance $\mathbf{\rm B}\left(f\right)$ within a Gaussian likelihood $p\left(\left.{\varvec{v}}\left(t,f\right)\right\vert {\varvec{\iota}}\left(f,t\right),\mathbf{\rm B}\left(f\right)\right)$. Henceforth, it is not essential to the main exposition to specify likelihood or a priori probability model formulas for this MAP1 as well as ${\varvec{\Lambda}}\left(f\right)$ or $\mathbf{\rm B}\left(f\right)$, which are determined by other methods.

2 $$\begin{array}{l}\widehat{{\varvec{\iota}}}\left(f,t\right)={argmax}_{{\varvec{\iota}}\left(f,t\right)}\left\{p\left(\left.{\varvec{\iota}}\left(f,t\right)\right\vert {\varvec{v}}\left(t,f\right),{\varvec{\Lambda}}\left(f\right)\right)\right\}\\ p\left(\left.{\varvec{\iota}}\left(f,t\right)\right\vert {\varvec{v}}\left(t,f\right),{\varvec{\Lambda}}\left(f\right)\right)\propto p\left(\left.{\varvec{v}}\left(t,f\right)\right\vert {\varvec{\iota}}\left(f,t\right)\right)p\left(\left.{\varvec{\iota}}\left(f,t\right)\right\vert {\varvec{\Lambda}}\left(f\right)\right)\end{array}$$

This first-step MAP1 (Eq. 2) carries on estimation errors (Fig. 1b) when posits ad hoc any a priori model $p\left(\left.{\varvec{\iota}}\left(f,t\right)\right\vert {\varvec{\Lambda}}\left(f\right)\right)$ and with covariance-matrix ${\varvec{\Lambda}}\left(f\right)$ having no relation whatsoever with the functional connectivity, whereas a GGS model $p\left(\left.{\varvec{\iota}}\left(f,t\right)\right\vert {{\varvec{\Theta}}}_{{\varvec{\iota}}{\varvec{\iota}}}\left(f\right)\right)$ actually governs brain oscillations ${\varvec{\iota}}\left(f,t\right)$. Then, the **multistep** inverse solution causes errors from the first step MAP1 estimate $\widehat{{\varvec{\iota}}}\left(f,t\right)$ (Eq. 2) to blow up in any second-step estimate ${\widehat{{\varvec{\Theta}}}}_{{\varvec{\iota}}{\varvec{\iota}}}\left(f\right)$.

At the same time, a first step MAP1 faces an obvious circularity when posits the actual GGS model $p\left(\left.{\varvec{\iota}}\left(f,t\right)\right\vert {{\varvec{\Theta}}}_{{\varvec{\iota}}{\varvec{\iota}}}\left(f\right)\right)$, where the precision-matrix ${{\varvec{\Theta}}}_{{\varvec{\iota}}{\varvec{\iota}}}\left(f\right)$ is unknown and must be the target of the second step MAP1. A similar circularity is dealt with estimating a covariance-matrix in a *Covariance Component Model* (CCM)^73–76^ or autoregressive-coefficient in a *State Space Model* (SSM)^48,77,78^. Here, this circularity is caused by a *Hidden GGS* (HIGGS) model for latent brain oscillations ${\varvec{\iota}}\left(f,t\right)$, similar to a CCM, but upon precision matrices and with a graphical sparse a priori model. The inverse solution in **onestep** bypassing this circularity is a MAP2 (Eq. 1)^59^.

### Second step MAP1 inverse solution for functional connectivity of the GGS model and estimation errors

**Figure 2:**
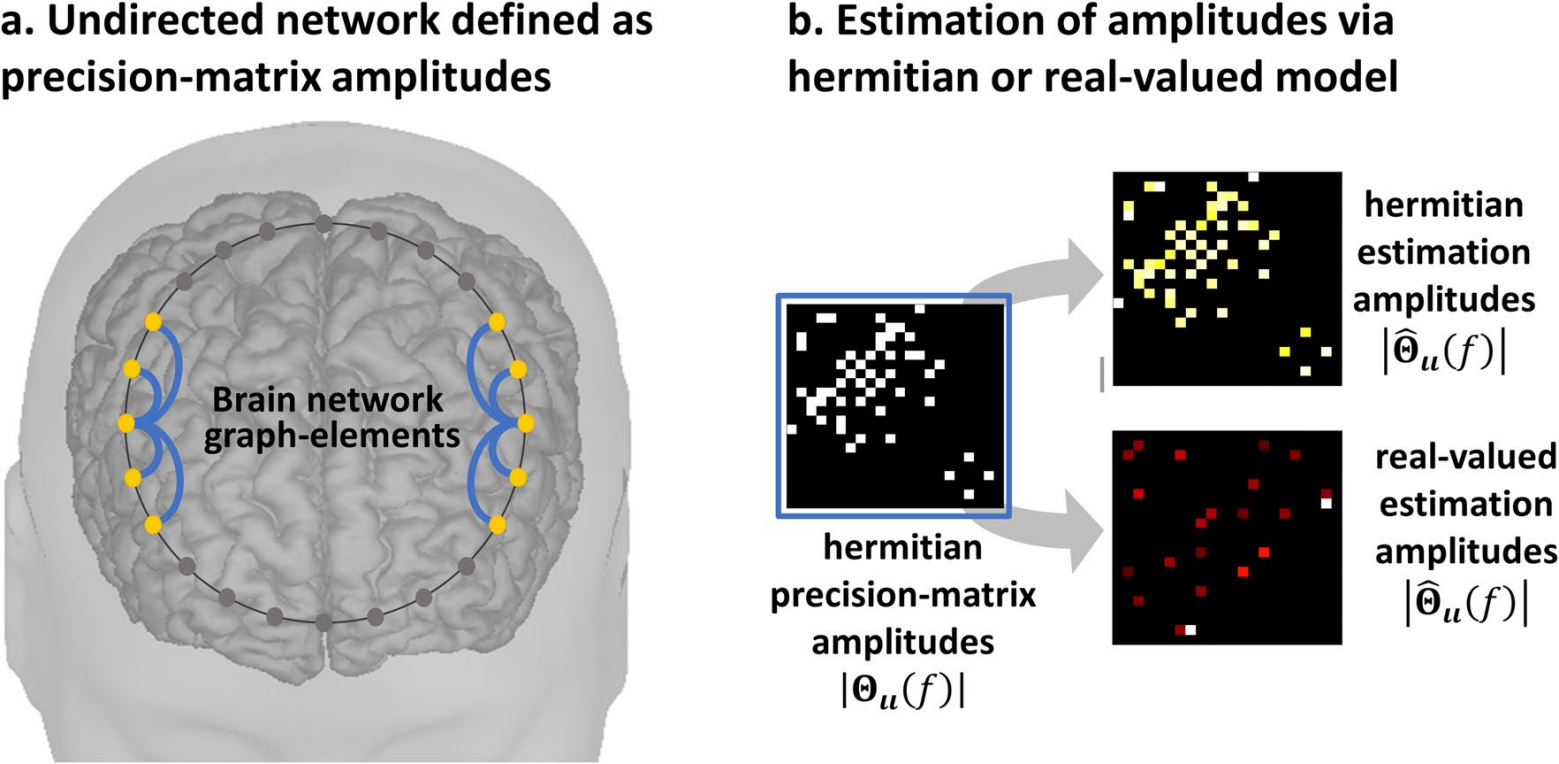
Connectivity distortions in the real-valued approximation of a Hermitian GGS model. (**a**) Undirected network as defined by the amplitude of Hermitian graph-elements or entries of the Hermitian GGS precision-matrix. (**b**) Binary GGS-precision-matrix amplitudes ${{\varvec{\Theta}}}_{{\varvec{\iota}}{\varvec{\iota}}}\left(f\right)$ are perfectly retrievd by estimation ${\widehat{{\varvec{\Theta}}}}_{{\varvec{\iota}}{\varvec{\iota}}}\left(f\right)$ based on a Hermitian GGS model with Hermitian graphical LASSO prior and distorted by estimation based on a real-valued GGS model with graphical LASSO prior.

The second step MAP1 is for the GGS precision matrix (Fig. 1b) ${{\varvec{\Theta}}}_{{\varvec{\iota}}{\varvec{\iota}}}\left(f\right)$ upon sampled covariance matrix (pseudodata) ${\widehat{{\varvec{\Sigma}}}}_{{\varvec{\iota}}{\varvec{\iota}}}\left(f\right)$ (Eq. 3) obtained from brain oscillation estimates $\widehat{{\varvec{\iota}}}\left(f,t\right)$ in the first step MAP1 (Eq. 2). The GGS model $p\left(\left.\widehat{{\varvec{\iota}}}\left(f,t\right)\right\vert {{\varvec{\Theta}}}_{{\varvec{\iota}}{\varvec{\iota}}}\left(f\right)\right)$ yields a Wishart first-type likelihood $p\left(\left.{\widehat{{\varvec{\Sigma}}}}_{{\varvec{\iota}}{\varvec{\iota}}}\left(f\right)\right\vert {{\varvec{\Theta}}}_{{\varvec{\iota}}{\varvec{\iota}}}\left(f\right)\right)$ for a sampled covariance-matrix ${\widehat{{\varvec{\Sigma}}}}_{{\varvec{\iota}}{\varvec{\iota}}}\left(f\right)$. Likelihood ill-conditioning is here in the sense of low condition order for any sampled covariance-matrix ${\widehat{{\varvec{\Sigma}}}}_{{\varvec{\iota}}{\varvec{\iota}}}\left(f\right)$ caused by a limited number of samples $\widehat{{\varvec{\iota}}}\left(f,t\right)$ in time-domain $t$^79^. A graphical a priori probability $p\left({{\varvec{\Theta}}}_{{\varvec{\iota}}{\varvec{\iota}}}\left(f\right)\right)$ regularizes this ill-conditioning with sparse selection of undirected graph elements and amplitude (Fig. 2a) of the Hermitian precision-matrix off-diagonal entries. The graphical a priori probability $p\left({{\varvec{\Theta}}}_{{\varvec{\iota}}{\varvec{\iota}}}\left(f\right)\right)$ base sparse selection upon an extension of the *Least Absolute Shrinkage and Selection Operator* (LASSO)^61^ denominated *graphical LASSO* (**gLASSO**) in real-valued multivariate statistics^35^.

3 $$\begin{array}{l}{\widehat{{\varvec{\Theta}}}}_{{\varvec{\iota}}{\varvec{\iota}}}\left(f\right)={argmax}_{{{\varvec{\Theta}}}_{{\varvec{\iota}}{\varvec{\iota}}}\left(f\right)}\left\{p\left(\left.{{\varvec{\Theta}}}_{{\varvec{\iota}}{\varvec{\iota}}}\left(f\right)\right\vert {\widehat{{\varvec{\Sigma}}}}_{{\varvec{\iota}}{\varvec{\iota}}}\left(f\right)\right)\right\}\\ p\left(\left.{{\varvec{\Theta}}}_{{\varvec{\iota}}{\varvec{\iota}}}\left(f\right)\right\vert {\widehat{{\varvec{\Sigma}}}}_{{\varvec{\iota}}{\varvec{\iota}}}\left(f\right)\right)\propto p\left(\left.{\widehat{{\varvec{\Sigma}}}}_{{\varvec{\iota}}{\varvec{\iota}}}\left(f\right)\right\vert {{\varvec{\Theta}}}_{{\varvec{\iota}}{\varvec{\iota}}}\left(f\right)\right)p\left({{\varvec{\Theta}}}_{{\varvec{\iota}}{\varvec{\iota}}}\left(f\right)\right)\end{array}$$

The second step MAP1 (Eq. 3) carries on estimation errors when posits a GGS model $p\left(\left.{\varvec{\iota}}\left(f,t\right)\right\vert {{\varvec{\Theta}}}_{{\varvec{\iota}}{\varvec{\iota}}}\left(f\right)\right)$ adhoc defined as real-valued (Fig. 2b), whereas a complex-valued Hermitian actually governs brain oscillations in the frequency domain^20,44–46^. Within such models functional connectivity parameters are Hermitian precision-matrix entries ${{\varvec{\Theta}}}_{{\varvec{\iota}}{\varvec{\iota}}}\left(f\right)$ or Hermitian graph-elements encoding phase and amplitude^30,80,81^. Indeed, estimation errors in the precision-matrix ${\widehat{{\varvec{\Theta}}}}_{{\varvec{\iota}}{\varvec{\iota}}}\left(f\right)$ even reflect wrong sparse selection of the amplitudes for the real-valued compared to the Hermitian one. Furthermore, this MAP1 in either case may also carry on estimation errors due to sparse bias^82^ or instability in large-scale dimensions^83^.

We present a novel MAP1 algorithm determining the precision matrix ${\widehat{{\varvec{\Theta}}}}_{{\varvec{\iota}}{\varvec{\iota}}}\left(f\right)$ (Eqs. 2, 5) with Bayesian hierarchical representation of the sparse Hermitian graphical a priori model or *Hermitian Graphical LASSO* (**hgLASSO**). We demonstrate in GGS model simulations with large-scale and Hermitian precision-matrices the unbiased performance of this **hgLASSO** algorithm, which provides estimates following the theoretical Rayleigh distribution^82^.

### Successive approximations to onestep MAP2 inverse solution for functional connectivity via multistep MAP1 inverse solution

**Figure 3:**
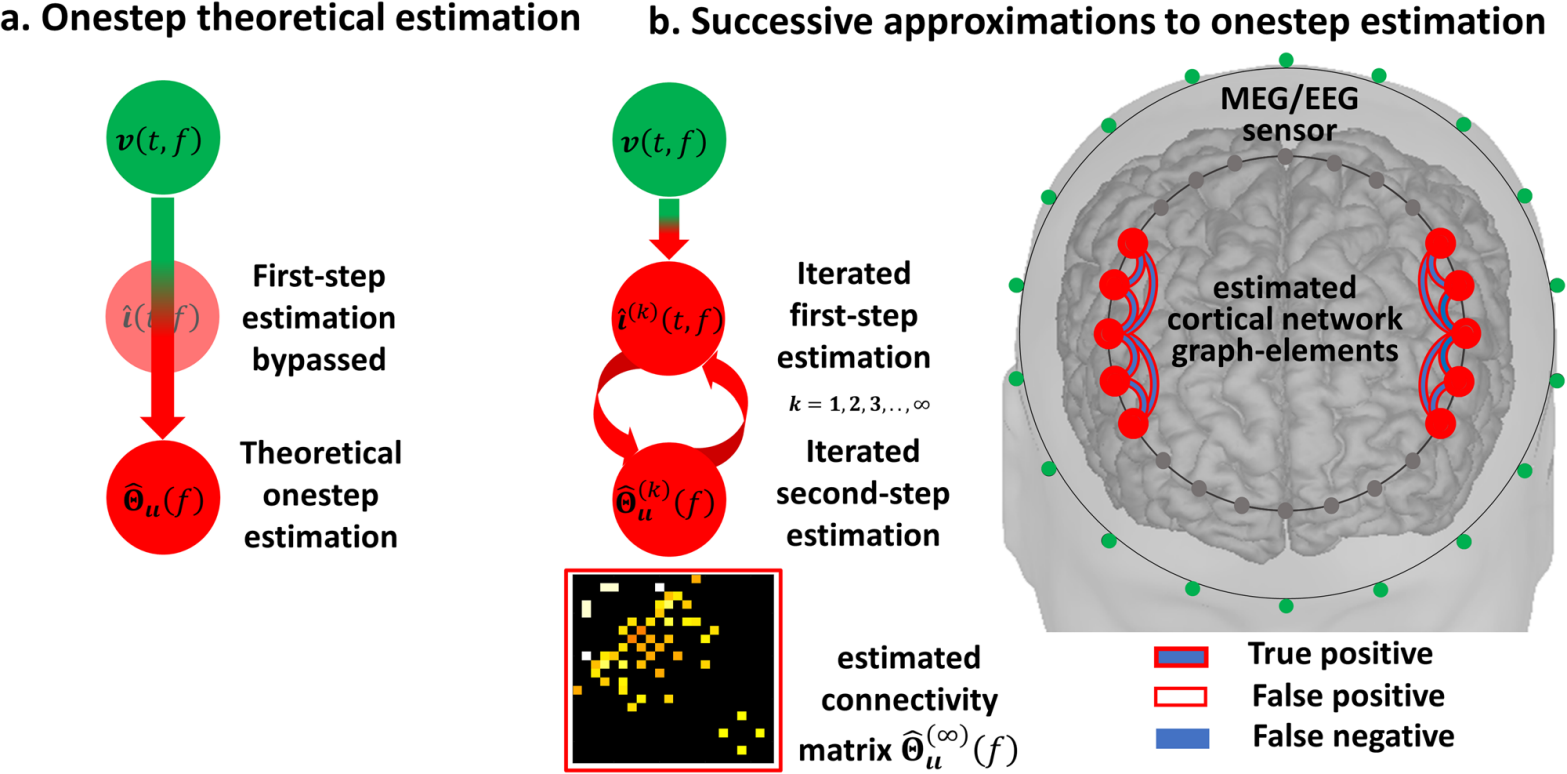
Theoretically correct procedure for the estimation of connectivity and to eliminate distortions. (**a**) The ideal but impractical solution is to bypass intermediate estimators $\widehat{{\varvec{\iota}}}\left(t,f\right)$ onestep search of optimal connectivity ${\widehat{{\varvec{\Theta}}}}_{{\varvec{\iota}}{\varvec{\iota}}}\left(f\right)$ explaining the observations. (**b**) Our implementation of efficient sequential approximations to the onestep procedure by means of Expectation Maximization iterations of ${\widehat{{\varvec{\iota}}}}^{\left(k\right)}\left(t,f\right)$ and ${\widehat{{\varvec{\Theta}}}}_{{\varvec{\iota}}{\varvec{\iota}}}^{\left(k\right)}\left(f\right)$ produces perfect functional graph-elements due to statistical guaranties.

A **onestep** MAP2 (Eq. 1) is for the HIGGS precision-matrix (Fig. 3a) ${{\varvec{\Theta}}}_{{\varvec{\iota}}{\varvec{\iota}}}\left(f\right)$ upon the MEG/EEG oscillations sampled covariance-matrix (data) ${\widehat{{\varvec{\Sigma}}}}_{{\varvec{v}}{\varvec{v}}}\left(f\right)$. The second-type likelihood $p\left(\left.{\widehat{{\varvec{\Sigma}}}}_{{\varvec{v}}{\varvec{v}}}\left(f\right)\right\vert {{\varvec{\Theta}}}_{{\varvec{\iota}}{\varvec{\iota}}}\left(f\right)\right)$ (Eq. 4) is obtained marginalizing ${\varvec{\iota}}\left(f,t\right)$ in a first-type likelihood $p\left(\left.{\varvec{v}}\left(t,f\right)\right\vert {\varvec{\iota}}\left(f,t\right)\right)$ under an a priori HIGGS model $p\left(\left.{\varvec{\iota}}\left(f,t\right)\right\vert {{\varvec{\Theta}}}_{{\varvec{\iota}}{\varvec{\iota}}}\left(f\right)\right)$. A MAP2 for similar models may be impractical and must commonly be approached by A*pproximated Bayesian Computation* (ABC)^84^. ABC employs successive approximations to the second-type likelihood $p\left(\left.{\widehat{{\varvec{\Sigma}}}}_{{\varvec{v}}{\varvec{v}}}\left(f\right)\right\vert {{\varvec{\Theta}}}_{{\varvec{\iota}}{\varvec{\iota}}}\left(f\right)\right)$ (Eq. 4)^85^ with Gibbs sampling of the a posteriori probability $p\left(\left.{{\varvec{\Theta}}}_{{\varvec{\iota}}{\varvec{\iota}}}\left(f\right)\right\vert {\widehat{{\varvec{\Sigma}}}}_{{\varvec{v}}{\varvec{v}}}\left(f\right)\right)$^86^. Gibbs sampling algorithms might be impractical even for an a posteriori probability of the second-step MAP1 $p\left(\left.{{\varvec{\Theta}}}_{{\varvec{\iota}}{\varvec{\iota}}}\left(f\right)\right\vert {\widehat{{\varvec{\Sigma}}}}_{{\varvec{\iota}}{\varvec{\iota}}}\left(f\right)\right)$ (Eq. 2)^63^ requiring alternative approaches^83,87^.

4 $$\begin{array}{l}{\widehat{{\varvec{\Theta}}}}_{{\varvec{\iota}}{\varvec{\iota}}}\left(f\right)={argmax}_{{{\varvec{\Theta}}}_{{\varvec{\iota}}{\varvec{\iota}}}\left(f\right)}\left\{p\left(\left.{{\varvec{\Theta}}}_{{\varvec{\iota}}{\varvec{\iota}}}\left(f\right)\right\vert {\widehat{{\varvec{\Sigma}}}}_{{\varvec{v}}{\varvec{v}}}\left(f\right)\right)\right\}\\ p\left(\left.{{\varvec{\Theta}}}_{{\varvec{\iota}}{\varvec{\iota}}}\left(f\right)\right\vert {\widehat{{\varvec{\Sigma}}}}_{{\varvec{v}}{\varvec{v}}}\left(f\right)\right)\propto p\left(\left.{\widehat{{\varvec{\Sigma}}}}_{{\varvec{v}}{\varvec{v}}}\left(f\right)\right\vert {{\varvec{\Theta}}}_{{\varvec{\iota}}{\varvec{\iota}}}\left(f\right)\right)p\left({{\varvec{\Theta}}}_{{\varvec{\iota}}{\varvec{\iota}}}\left(f\right)\right)\end{array}$$

These successive approximations to a second-type likelihood (Fig. 3b) may be implemented through the *Expectation Maximization* (EM) algorithm as in a CCM^73–76^. EM is interpretable as first-step (Eq. 2) and second-step (Eq. 3) MAP1 estimators obtaining a MAP2 local optima^88^: where the EM maximization stage is a second-step MAP1 in a loop $\left(k\right)$ with $p\left(\left.{{\varvec{\Theta}}}_{{\varvec{\iota}}{\varvec{\iota}}}\left(f\right)\right\vert {\widehat{{\varvec{\Psi}}}}_{{\varvec{\iota}}{\varvec{\iota}}}^{\left(k\right)}\left(f\right)\right)$ (Eq. 5) computed from an equivalent first-type likelihood $p\left(\left.{\widehat{{\varvec{\Psi}}}}_{{\varvec{\iota}}{\varvec{\iota}}}^{\left(k\right)}\left(f\right)\right\vert {{\varvec{\Theta}}}_{{\varvec{\iota}}{\varvec{\iota}}}\left(f\right)\right)$ (Eq. 3). This first-type likelihood is interpreted as a GGS model explaining an expected covariance-matrix ${\widehat{{\varvec{\Psi}}}}_{{\varvec{\iota}}{\varvec{\iota}}}^{\left(k\right)}\left(f\right)$ upon the latent precision-matrix ${{\varvec{\Theta}}}_{{\varvec{\iota}}{\varvec{\iota}}}\left(f\right)$ and the a priori probability $p\left({{\varvec{\Theta}}}_{{\varvec{\iota}}{\varvec{\iota}}}\left(f\right)\right)$.

5 $$\begin{array}{l}{\widehat{{\varvec{\Theta}}}}_{{\varvec{\iota}}{\varvec{\iota}}}^{\left(k+1\right)}\left(f\right)={argmax}_{{{\varvec{\Theta}}}_{{\varvec{\iota}}{\varvec{\iota}}}\left(f\right)}\left\{p\left(\left.{{\varvec{\Theta}}}_{{\varvec{\iota}}{\varvec{\iota}}}\left(f\right)\right\vert {\widehat{{\varvec{\Psi}}}}_{{\varvec{\iota}}{\varvec{\iota}}}^{\left(k\right)}\left(f\right)\right)\right\}\\ p\left(\left.{{\varvec{\Theta}}}_{{\varvec{\iota}}{\varvec{\iota}}}\left(f\right)\right\vert {\widehat{{\varvec{\Psi}}}}_{{\varvec{\iota}}{\varvec{\iota}}}^{\left(k\right)}\left(f\right)\right)\propto p\left(\left.{\widehat{{\varvec{\Psi}}}}_{{\varvec{\iota}}{\varvec{\iota}}}^{\left(k\right)}\left(f\right)\right\vert {{\varvec{\Theta}}}_{{\varvec{\iota}}{\varvec{\iota}}}\left(f\right)\right)p\left({{\varvec{\Theta}}}_{{\varvec{\iota}}{\varvec{\iota}}}\left(f\right)\right)\end{array}$$

The expected covariance matrix ${\widehat{{\varvec{\Psi}}}}_{{\varvec{\iota}}{\varvec{\iota}}}^{\left(k\right)}\left(f\right)$ (Eq. 5) is determined from an ensemble covariance matrix ${\widehat{{\varvec{\Pi}}}}_{{\varvec{\iota}}{\varvec{\iota}}}^{\left(k\right)}\left(f\right)$, and its sampled estimator ${\widehat{{\varvec{\Sigma}}}}_{{\varvec{\iota}}{\varvec{\iota}}}^{\left(k\right)}\left(f\right)$ in the expectation stage MAP1^89^ is also common for a first-step MAP1^62,65–71^.

6 $${\widehat{{\varvec{\Psi}}}}_{{\varvec{\iota}}{\varvec{\iota}}}^{\left(k\right)}\left(f\right)={\widehat{{\varvec{\Sigma}}}}_{{\varvec{\iota}}{\varvec{\iota}}}^{\left(k\right)}\left(f\right)+{\widehat{{\varvec{\Pi}}}}_{{\varvec{\iota}}{\varvec{\iota}}}^{\left(k\right)}\left(f\right)$$

The EM expectation stage obtaining ${\widehat{{\varvec{\Psi}}}}_{{\varvec{\iota}}{\varvec{\iota}}}^{\left(k\right)}\left(f\right)$ is a first-step MAP1 in a loop $\left(k\right)$ with $p\left(\left.{\varvec{\iota}}\left(f,t\right)\right\vert {\varvec{v}}\left(t,f\right),{\widehat{{\varvec{\Theta}}}}_{{\varvec{\iota}}{\varvec{\iota}}}^{\left(k\right)}\left(f\right)\right)$ (Eq. 7) computed from an equivalent first-type likelihood $p\left(\left.{\varvec{v}}\left(t,f\right)\right\vert {\varvec{\iota}}\left(f,t\right)\right)$ (Eq. 2) and positing an a priori GGS model $p\left(\left.{\varvec{\iota}}\left(f,t\right)\right\vert {\widehat{{\varvec{\Theta}}}}_{{\varvec{\iota}}{\varvec{\iota}}}^{\left(k\right)}\left(f\right)\right)$ upon the precision-matrix ${\widehat{{\varvec{\Theta}}}}_{{\varvec{\iota}}{\varvec{\iota}}}^{\left(k\right)}\left(f\right)$, which is determined in the previous maximization stage. The MAP2 circularity is then solved by successive approximations in a multistep loop $\left(k\right)$ with maximization (Eq. 5) and expectation (Eq. 6) specifying the actual HIGGS model.

7 $$\begin{array}{l}{\widehat{{\varvec{\iota}}}}^{\left(k\right)}\left(f,t\right)={argmax}_{{\varvec{\iota}}\left(f,t\right)}\left\{p\left(\left.{\varvec{\iota}}\left(f,t\right)\right\vert {\varvec{v}}\left(t,f\right),{\widehat{{\varvec{\Theta}}}}_{{\varvec{\iota}}{\varvec{\iota}}}^{\left(k\right)}\left(f\right)\right)\right\}\\ p\left(\left.{\varvec{\iota}}\left(f,t\right)\right\vert {\varvec{v}}\left(t,f\right),{\widehat{{\varvec{\Theta}}}}_{{\varvec{\iota}}{\varvec{\iota}}}^{\left(k\right)}\left(f\right)\right)\propto p\left(\left.{\varvec{v}}\left(t,f\right)\right\vert {\varvec{\iota}}\left(f,t\right)\right)p\left(\left.{\varvec{\iota}}\left(f,t\right)\right\vert {\widehat{{\varvec{\Theta}}}}_{{\varvec{\iota}}{\varvec{\iota}}}^{\left(k\right)}\left(f\right)\right)\end{array}$$

A proof of concept for the HIGGS model in EEG simulations reveals that **multistep** inverse solutions (Eqs. 2, 3) may produce a very distorted precision matrix ${\widehat{{\varvec{\Theta}}}}_{{\varvec{\iota}}{\varvec{\iota}}}\left(f\right)$ in a second step MAP1 (Eq. 3). When even no ill-conditioning holds (ruling out leakage) in a first step MAP1 (Eq. 2) determining the brain oscillations $\widehat{{\varvec{\iota}}}\left(f,t\right)$. In the same situation, the **onestep** inverse solution (Eqs. 5, 6, 7) determines ${\widehat{{\varvec{\Theta}}}}_{{\varvec{\iota}}{\varvec{\iota}}}\left(f\right)$ exactly. We employ the sparse Hermitian graphical a priori model to determine ${\widehat{{\varvec{\Theta}}}}_{{\varvec{\iota}}{\varvec{\iota}}}\left(f\right)$ via the unbiased **hgLASSO** algorithm for both **multistep** (Eq. 3) and **onestep** (Eq. 5) inverse solutions. Implementations of **onestep** inverse solutions that violate such unbiasedness conditions with other Hermitian graphical a priori models also outperform **multistep hgLASSO** inverse solutions. The **multistep** procedure is then the systemic cause of distortions irremediable even with an exact second-step inverse solution for ${\widehat{{\varvec{\Theta}}}}_{{\varvec{\iota}}{\varvec{\iota}}}\left(f\right)$. This proof of concept also reveals that **multistep** inverse solutions employing a sparse real-valued graphical a priori model or *Graphical LASSO* (**gLASSO**) that approximates the HIGGS model further distorts ${\widehat{{\varvec{\Theta}}}}_{{\varvec{\iota}}{\varvec{\iota}}}\left(f\right)$. We confirm this difference in performance between **multistep** and **onestep** procedures by comparing EEG inverse solutions against their fine-grained ECoG reference inverse solutions in macaque simultaneous recordings.

## Materials and methods

### Gaussian graphical spectral (GGS) model and MAP1 inverse solution with Hermitian graphical LASSO (hgLASSO)

#### GGS model and interpretations of functional connectivity

The PSP processes myriad fulfills some frequency-domain mixing conditions and consequently Gaussianity in the complex-valued follows for brain oscillations ${\varvec{\iota}}\left(t,f\right)$^4,28,90,91^. Then, without loss of generality, the *Gaussian graphical spectral* (GGS) model (Eq. 8) with complex-valued Hermitian precision matrix ${{\varvec{\Theta}}}_{{\varvec{\iota}}{\varvec{\iota}}}\left(f\right)$ is the functional network model for brain oscillations. This GGS model is valid at any frequency $f$ within the human spectrum ($f\in {\mathbb{F}}$) from 0.5 to 140 Hz^18,49,92^ and within a resting state or task transient ($\forall t\in {\mathbb{T}}$) from a block design of approximately 2 s^23–27^.

8 $$p\left({\varvec{\iota}}\left(t,f\right)\left\vert {{\varvec{\Theta}}}_{{\varvec{\iota}}{\varvec{\iota}}}\left(f\right)\right.\right)={N}^{\mathbb{C}}\left({\varvec{\iota}}\left(t,f\right)\vert 0,{{\varvec{\Theta}}}_{{\varvec{\iota}}{\varvec{\iota}}}^{-1}\left(f\right)\right)$$

where the complex-valued precision matrix ${{\varvec{\Theta}}}_{{\varvec{\iota}}{\varvec{\iota}}}\left(f\right)$, or inverse of the cross-spectrum ${{\varvec{\Sigma}}}_{{\varvec{\iota}}{\varvec{\iota}}}\left(f\right)$ (${{\varvec{\Theta}}}_{{\varvec{\iota}}{\varvec{\iota}}}\left(f\right)={{\varvec{\Sigma}}}_{{\varvec{\iota}}{\varvec{\iota}}}^{-1}\left(f\right)$), defines within off-diagonal functional connectivity parameters as Hermitian graph elements^30,80,81^. Functional connectivity proxies are then a function of the Hermitian tensor $\left\{{{\varvec{\Theta}}}_{{\varvec{\iota}}{\varvec{\iota}}}\left(f\right):\forall f\in {\mathbb{F}})\right\}$ at all frequencies^20,44–46,50^. This matrix ${{\varvec{\Theta}}}_{{\varvec{\iota}}{\varvec{\iota}}}\left(f\right)$ may also be representing a time–frequency connectivity behavior ${{\varvec{\Theta}}}_{{\varvec{\iota}}{\varvec{\iota}}}\left({t}_{0},f\right)$ for a transient ${\mathbb{T}}=\left({t}_{0},{t}_{0}+\Delta t\right)$ in a sliding in a slower timescale ${t}_{0}$. This timescale ${t}_{0}$ is the domain for evolving network configurations as represented across task or resting state transients.

A first interpretation of these Hermitian graph elements is amplitude $\left\{\left\vert {{\varvec{\Theta}}}_{{\varvec{\iota}}{\varvec{\iota}}}\left(g,{g}{\prime};f\right)\right\vert \right\}$ encoding undirected edges $\left\{g\leftrightarrow g{\prime}\right\}$ at a given frequency ($f\in {\mathbb{F}}$). This interpretation is also common for a real-valued and symmetric approximation for ${{\varvec{\Theta}}}_{{\varvec{\iota}}{\varvec{\iota}}}\left(f\right)$ due to a GGS model describing ${\varvec{\iota}}\left(t,f\right)$ as a narrow band filtered process.

A second interpretation is phase lag across frequencies in $\left\{{{\varvec{\Theta}}}_{{\varvec{\iota}}{\varvec{\iota}}}\left(g,{g}{\prime};f\right):\forall f\in {\mathbb{F}})\right\}$ encoding directed edges $\left\{\dots ,g\leftarrow g{\prime},\dots \right\}$, which are determined by other functional connectivity proxies such as coherences and the phase slope^93,94^. A real-valued and symmetric approximation for ${{\varvec{\Theta}}}_{{\varvec{\iota}}{\varvec{\iota}}}\left(f\right)$ misses this phase information and is limited interpreted to encode the degree of correlation or anticorrelation interpreted with the sign in $\left\{{{\varvec{\Theta}}}_{{\varvec{\iota}}{\varvec{\iota}}}\left(g,{g}{\prime};f\right)\right\}$^21,40,95^.

A third interpretation of the Hermitian graph elements (${{\varvec{\Theta}}}_{{\varvec{\iota}}{\varvec{\iota}}}\left(f\right)$;$\forall f\in {\mathbb{F}}$) employed here in GGS model simulations here follows from transient second-order stochastic stationarity due to time-domain mixing conditions. The time domain ${\varvec{\iota}}\left(t\right)$, as expressed by the stochastic integral (Eq. 9), is driven by the convolution kernel ${\mathbf{\rm K}}_{{\varvec{\iota}}{\varvec{\iota}}}\left(\tau \right)$ ($\forall \tau ,t\in {\mathbb{T}}$) and the perturbative process ${\varvec{\zeta}}\left(t\right)$.

9 $${\varvec{\iota}}\left(t\right)={\int }_{0}^{t}{\mathbf{\rm K}}_{{\varvec{\iota}}{\varvec{\iota}}}\left(\tau \right){\varvec{\iota}}\left(t-\tau \right)d\tau +{\varvec{\zeta}}\left(t\right)$$

where the convolution kernel ${\mathbf{\rm K}}_{{\varvec{\iota}}{\varvec{\iota}}}\left(\tau \right)$ encodes multiply time lagged causal relations ($\forall \tau \in {\mathbb{T}}$) that are physically interpreted as synaptic conductance values and axonal delays^46,51,78,96–99^.

The relation between the precision-matrix ${{\varvec{\Theta}}}_{{\varvec{\iota}}{\varvec{\iota}}}\left(f\right)$ in (Eq. 1) and the convolution kernel ${\mathbf{\rm K}}_{{\varvec{\iota}}{\varvec{\iota}}}\left(\tau \right)$ follows from analogous GGS model conditions held by the frequency-domain perturbative process ${\varvec{\zeta}}\left(t,f\right)$ with precision-matrix ${{\varvec{\Theta}}}_{{\varvec{\zeta}}{\varvec{\zeta}}}\left(f\right)$ in (Eq. 10).

10 $$\begin{array}{l}{\varvec{\iota}}\left(t,f\right)={\mathbf{\rm K}}_{{\varvec{\iota}}{\varvec{\iota}}}\left(f\right){\varvec{\iota}}\left(t,f\right)+{\varvec{\zeta}}\left(t,f\right)\\ p\left({\varvec{\zeta}}\left(t,f\right)\left\vert {{\varvec{\Theta}}}_{{\varvec{\zeta}}{\varvec{\zeta}}}\left(f\right)\right.\right)={N}^{\mathbb{C}}\left({\varvec{\zeta}}\left(t,f\right)\vert 0,{{\varvec{\Theta}}}_{{\varvec{\zeta}}{\varvec{\zeta}}}^{-1}\left(f\right)\right)\end{array}$$

where the frequency-domain kernel ${\mathbf{\rm K}}_{{\varvec{\iota}}{\varvec{\iota}}}\left(f\right)$ and precision-matrix ${{\varvec{\Theta}}}_{{\varvec{\zeta}}{\varvec{\zeta}}}\left(f\right)$ are spectral factors for ${{\varvec{\Theta}}}_{{\varvec{\iota}}{\varvec{\iota}}}\left(f\right)$ (Eq. 11)^100–103^. Then, from the kernel or directed transfer function ${\mathbf{\rm K}}_{{\varvec{\iota}}{\varvec{\iota}}}\left(f\right)$ and precision-matrix ${{\varvec{\Theta}}}_{{\varvec{\zeta}}{\varvec{\zeta}}}\left(f\right)$, other functional connectivity proxies such as directed partial coherence or granger causality index encode directed edges $\left\{\dots ,g\leftarrow g{\prime},\dots \right\}$^13,48,104^.

11 $$\begin{array}{l}p\left({\varvec{\iota}}\left(t,f\right)\left\vert {{\varvec{\Theta}}}_{{\varvec{\iota}}{\varvec{\iota}}}\left(f\right)\right.\right)={N}^{\mathbb{C}}\left({\varvec{\iota}}\left(t,f\right)\vert 0,{{\varvec{\Theta}}}_{{\varvec{\iota}}{\varvec{\iota}}}^{-1}\left(f\right)\right)\\ {{\varvec{\Theta}}}_{{\varvec{\iota}}{\varvec{\iota}}}\left(f\right)={\left(\mathbf{I}-{\mathbf{\rm K}}_{{\varvec{\iota}}{\varvec{\iota}}}\left(f\right)\right)}^{\dagger}{{\varvec{\Theta}}}_{{\varvec{\zeta}}{\varvec{\zeta}}}\left(f\right)\left(\mathbf{I}-{\mathbf{\rm K}}_{{\varvec{\iota}}{\varvec{\iota}}}\left(f\right)\right)\end{array}$$

#### GGS model MAP1 inverse solution for functional connectivity and Hermitian graphical a priori probabilities

Assume, for the moment, directly observed cortical oscillations ${\varvec{\iota}}\left(t,f\right)$ explained by the GGS model (Eq. 8). Estimating the precision-matrix ${{\varvec{\Theta}}}_{{\varvec{\iota}}{\varvec{\iota}}}\left(f\right)$ in this GGS model (Eq. 8) may be regarded as a pseudoinverse for the sampled covariance-matrix ${\widehat{{\varvec{\Sigma}}}}_{{\varvec{\iota}}{\varvec{\iota}}}\left(f\right)$ (Eq. 12) for several samples ($\forall t\in {\mathbb{T}}$), where the sample size $T=\left\vert {\mathbb{T}}\right\vert$.

12 $${\widehat{{\varvec{\Sigma}}}}_{{\varvec{\iota}}{\varvec{\iota}}}\left(f\right)=\frac{1}{T}{\sum }_{t\in {\mathbb{T}}}{\varvec{\iota}}\left(t,f\right){{\varvec{\iota}}\left(t,f\right)}^{\dagger}$$

The first-type likelihood explaining the sampled covariance-matrix ${\widehat{{\varvec{\Sigma}}}}_{{\varvec{\iota}}{\varvec{\iota}}}\left(f\right)$ (Eq. 10) is a complex-valued Wishart (Eq. 13), with degree of freedom $T$ and scale matrix ${T}^{-1}{{\varvec{\Theta}}}_{{\varvec{\iota}}{\varvec{\iota}}}^{-1}\left(f\right)$. The a priori probability^35^ is a Gibbs form (Eq. 13) upon a given scalar penalty function $\mathrm{P}$ for ${{\varvec{\Theta}}}_{{\varvec{\iota}}{\varvec{\iota}}}\left(f\right)$, a scale parameter ${\alpha }_{{\varvec{\iota}}}$ and a selection matrix ${\mathbf{A}}_{{\varvec{\iota}}{\varvec{\iota}}}$.

13 $$\begin{array}{l} {p\left( {\left. {{{\widehat \Sigma }_{\iota \iota }}\left( f \right)} \right|{\Theta _{\iota \iota }}\left( f \right)} \right) = {W^{\mathbb{C}}}\left( {{{\widehat \Sigma }_{\iota \iota }}\left( f \right)|{T^{ - 1}}\Theta _{\iota \iota }^{ - 1}\left( f \right),T} \right)} \hfill \\ {p\left( {{\Theta _{\iota \iota }}\left( f \right)} \right) = exp\left( {{\text{P}}\left( {{{\bf{A}}_{\iota \iota }} \odot {\Theta _{\iota \iota }}\left( f \right)} \right)|{\alpha _{\iota \iota }}T} \right)} \hfill \\ \end{array}$$

where ${\mathbf{A}}_{{\varvec{\iota}}{\varvec{\iota}}}$ shall be a matrix of ones that places priors only the off-diagonal elements ${{\varvec{\Theta}}}_{{\varvec{\iota}}{\varvec{\iota}}}\left(\nu \right)$, i.e., ${\mathbf{A}}_{{\varvec{\iota}}{\varvec{\iota}}}\left(i,i\right)=0$ for all $i$ and ${\mathbf{A}}_{{\varvec{\iota}}{\varvec{\iota}}}\left(i,j\right)=1$ for $i\ne j$. In subsequent work, this matrix will be used to reflect a priori connectivity information, for example, from anatomical data^53^. Then, with ${\widehat{{\varvec{\Sigma}}}}_{{\varvec{\iota}}{\varvec{\iota}}}\left(f\right)$ directly observed or determined from a first step (Eq. 2), MAP1 ${\widehat{{\varvec{\Theta}}}}_{{\varvec{\iota}}{\varvec{\iota}}}\left(f\right)$ (Eq. 3) based on a posteriori probability $p\left(\left.{{\varvec{\Theta}}}_{{\varvec{\iota}}{\varvec{\iota}}}\left(f\right)\right\vert {\widehat{{\varvec{\Sigma}}}}_{{\varvec{\iota}}{\varvec{\iota}}}\left(f\right)\right)$ from the GGS model likelihood and a priori probability (Eq. 13).

14 $$\begin{array}{l} {{{\widehat {\boldsymbol{\Theta }}}_{{\boldsymbol{\iota \iota }}}}\left( f \right) = argma{x_{{{\boldsymbol{\Theta }}_{{\boldsymbol{\iota \iota }}}}\left( f \right)}}\left\{ {p\left( {\left. {{{\boldsymbol{\Theta }}_{{\boldsymbol{\iota \iota }}}}\left( f \right)} \right|{{\widehat {\boldsymbol{\Sigma }}}_{{\boldsymbol{\iota \iota }}}}\left( f \right)} \right)} \right\}} \hfill \\ {p\left( {\left. {{{\boldsymbol{\Theta }}_{{\boldsymbol{\iota \iota }}}}\left( f \right)} \right|{{\widehat {\boldsymbol{\Sigma }}}_{{\boldsymbol{\iota \iota }}}}\left( f \right)} \right) \propto {W^{\mathbb C}}\left( {{{\widehat {\boldsymbol{\Sigma }}}_{{\boldsymbol{\iota \iota }}}}\left( f \right)|{T^{ - 1}}{\boldsymbol{\Theta }}_{{\boldsymbol{\iota \iota }}}^{ - 1}\left( f \right),T} \right)exp\left( {{\text{P}}\left( {{{\boldsymbol{A}}_{{\boldsymbol{\iota \iota }}}} \odot {{\boldsymbol{\Theta }}_{{\boldsymbol{\iota \iota }}}}\left( f \right)} \right)|{\alpha _{\boldsymbol{\iota }}}T} \right)} \hfill \\ \end{array}$$

The equivalent problem of maximizing this a posteriori probability in MAP1 (Eq. 14) is commonly expressed in the literature as minimizing the $-log$ transformation (Eq. 15) as a penalized cost function $\mathcal{L}\left({\varvec{\Theta}}\left(f\right)\right)=-log p\left({{\varvec{\Theta}}}_{{\varvec{\iota}}{\varvec{\iota}}}\left(f\right)\vert {\widehat{{\varvec{\Sigma}}}}_{{\varvec{\iota}}{\varvec{\iota}}}\left(f\right)\right)$.

15 $$\begin{array}{l}{\widehat{{\varvec{\Theta}}}}_{{\varvec{\iota}}{\varvec{\iota}}}\left(f\right)={argmin}_{{{\varvec{\Theta}}}_{{\varvec{\iota}}{\varvec{\iota}}}\left(f\right)}\left\{\mathcal{L}\left({{\varvec{\Theta}}}_{{\varvec{\iota}}{\varvec{\iota}}}\left(f\right)\right)\right\}\\ \mathcal{L}\left({{\varvec{\Theta}}}_{{\varvec{\iota}}{\varvec{\iota}}}\left(f\right)\right)=-log\left\vert {{\varvec{\Theta}}}_{{\varvec{\iota}}{\varvec{\iota}}}\left(f\right)\right\vert +tr\left({\widehat{{\varvec{\Sigma}}}}_{{\varvec{\iota}}{\varvec{\iota}}}\left(f\right){{\varvec{\Theta}}}_{{\varvec{\iota}}{\varvec{\iota}}}\left(f\right)\right)+{\alpha }_{{\varvec{\iota}}}\mathrm{P}\left({\mathbf{A}}_{{\varvec{\iota}}{\varvec{\iota}}}\odot {{\varvec{\Theta}}}_{{\varvec{\iota}}{\varvec{\iota}}}\left(f\right)\right)\end{array}$$

The *Hermitian Graphical Naïve* (**hgNaïve**) estimator (a priori free $\mathrm{P}=0$ in Eq. 15) is the inverse of the sample cross-spectral matrix ${\widehat{{\varvec{\Theta}}}}_{{\varvec{\iota}}{\varvec{\iota}}}\left(f\right)\leftarrow {\widehat{{\varvec{\Sigma}}}}_{{\varvec{\iota}}{\varvec{\iota}}}^{-1}\left(f\right)$ and usually yields a quite dense matrix with many spurious connectivities, which suggests the use of different penalizations. Then, we introduce (Eq. 15) the L2 norm $\mathrm{P}={\Vert \bullet \Vert }_{2}^{2}$, the *Hermitian Graphical Ridge* (**hgRidge**)^105,106^, the L1 norm $\mathrm{P}={\Vert \bullet \Vert }_{1}$, the *Hermitian Graphical LASSO* (**hgLASSO**)^81^, known as *Graphical LASSO* (**gLASSO**), in the real variable^35^.

In this paper, we shall emphasize that **hgLASSO** is the a priori model proposed to produce unbiased sparse estimates with the optimum of the target function (Eq. 12), where **hgNaïve** and **hgRidge** as well as **gLASSO** are hereinafter model violations. The critical issues are stable and scalable **hgLASSO** calculations in ultrahigh matrix dimensions preserving unbiasedness. We introduce a novel algorithm reformulating MAP1 (Eq. 14) with Bayesian hierarchical **hgLASSO**. This algorithm is plugged into multistep, and **onestep** methods are deferred to avoid interrupting the flow of exposition 2.3 Hermitian graphical LASSO (hgLASSO**)**.

### Hidden Gaussian graphical spectral (HIGGS) model and inverse solutions via multistep MAP1 and onestep MAP2

#### HIGGS model underneath the Gaussian EFM spectral equivalent.

As with latent brain activity ${\varvec{\iota}}\left(t\right)$, the conversion of brain oscillations ${\varvec{\iota}}\left(t,f\right)$ is given according to the same Electromagnetic Forward Model (EFM)^7–9^. The EFM forward operator ${\mathbf{L}}_{{\varvec{v}}{\varvec{\iota}}}$ (Eq. 18) describes in the time domain ($\forall t\in {\mathbb{T}}$) and spectral domain ($\forall f\in {\mathbb{F}}$) a purely linear and stationary measurement process. These measurements possess no relation whatsoever with any other biological mechanism and are only perturbed by a spectral noise process ${\varvec{\xi}}\left(t,f\right)$ at the sensors. Note the alternative, fMRI observations, suffer from temporal/spectral (also spatial) deformations due to the nonlinear metabolic-hemodynamic forward model of the Blood Oxygenation Level Depend (BOLD) signal which is acquired with a very poor temporal resolution.

16 $${\varvec{v}}\left(t,f\right)={\mathbf{L}}_{{\varvec{v}}{\varvec{\iota}}}{\varvec{\iota}}\left(t,f\right)+{\varvec{\xi}}\left(t,f\right)$$

Hereinafter, EFM represents cortical activity due to the high sensitivity of MEG/EEG to the activity of pyramidal layers within the cortical columnar organization^6,107^. Estimating activity for noncortical structures encounters another problem^108^ that must be addressed by some compensation measures for their bias to zero^109^.

Due to similar mixing conditions, the spectral noise process ${\varvec{\xi}}\left(t,f\right)$ is asymptotically Gaussian with precision matrix ${{\varvec{\Theta}}}_{{\varvec{\xi}}{\varvec{\xi}}}\left(f\right)$, which is valid at any frequency ($f\in {\mathbb{F}}$) and time-domain ($\forall t\in {\mathbb{T}}$)^20,28^. This is not accurate for the time-domain noise process ${\varvec{\xi}}\left(t\right)$ commonly assumed in an equivalent EFM of MEG/EEG data ${\varvec{v}}\left(t\right)$ (Eq. 16).

Then, this EFM Gaussian spectral equivalent represents the first-type likelihood explaining MEG/EEG oscillations (data) ${\varvec{v}}\left(t,f\right)$ upon the latent brain oscillation parameters ${\varvec{\iota}}\left(t,f\right)$ (Eq. 17). The a priori probability for parameters ${\varvec{\iota}}\left(t,f\right)$ (Eq. 8) specifies the Hidden GGS (HIGGS) explaining ${\varvec{\iota}}\left(t,f\right)$ upon the precision-matrix ${{\varvec{\Theta}}}_{{\varvec{\iota}}{\varvec{\iota}}}\left(f\right)$. In addition, the hyperparameters ${\varvec{\Omega}}\left(f\right)$ specified as precision matrices ${\varvec{\Omega}}\left(f\right)=\left\{{{\varvec{\Theta}}}_{{\varvec{\iota}}{\varvec{\iota}}}\left(f\right),{{\varvec{\Theta}}}_{{\varvec{\xi}}{\varvec{\xi}}}\left(f\right)\right\}$ within this HIGGS model require some a priori probability model $p\left({\varvec{\Omega}}\left(f\right)\right)$.

17 $$\begin{array}{l}p\left(\left.{\varvec{v}}\left(t,f\right)\right\vert {\varvec{\iota}}\left(t,f\right),{{\varvec{\Theta}}}_{{\varvec{\xi}}{\varvec{\xi}}}\left(f\right)\right)={N}^{\mathbb{C}}\left({\varvec{v}}\left(t,f\right)\vert {\mathbf{L}}_{{\varvec{v}}{\varvec{\iota}}}{\varvec{\iota}}\left(t,f\right),{{\varvec{\Theta}}}_{{\varvec{\xi}}{\varvec{\xi}}}^{-1}\left(f\right)\right)\\ p\left(\left.{\varvec{\iota}}\left(t,f\right)\right\vert {{\varvec{\Theta}}}_{{\varvec{\iota}}{\varvec{\iota}}}\left(f\right)\right)={N}^{\mathbb{C}}\left({\varvec{\iota}}\left(t,f\right)\vert 0,{{\varvec{\Theta}}}_{{\varvec{\iota}}{\varvec{\iota}}}^{-1}\left(f\right)\right)\\ p\left({\varvec{\Omega}}\left(f\right)\right)=p\left({{\varvec{\Theta}}}_{{\varvec{\iota}}{\varvec{\iota}}}\left(f\right)\right)p\left({{\varvec{\Theta}}}_{{\varvec{\xi}}{\varvec{\xi}}}\left(f\right)\right)\end{array}$$

#### HIGGS model MAP1 inverse solution for brain oscillations

We introduce the first step MAP1 based on the HIGGS model (Eq. 17), with first-type likelihood ${N}^{\mathbb{C}}\left({\varvec{v}}\left(t,f\right)\vert {\mathbf{L}}_{{\varvec{v}}{\varvec{\iota}}}{\varvec{\iota}}\left(t,f\right),{{\varvec{\Theta}}}_{{\varvec{\xi}}{\varvec{\xi}}}^{-1}\left(f\right)\right)$ and a priori probability ${N}^{\mathbb{C}}\left({\varvec{\iota}}\left(t,f\right)\vert 0,{{\varvec{\Theta}}}_{{\varvec{\iota}}{\varvec{\iota}}}^{-1}\left(f\right)\right)$, upon hyperparameters ${\varvec{\Omega}}\left(f\right)=\left\{{{\varvec{\Theta}}}_{{\varvec{\iota}}{\varvec{\iota}}}\left(f\right),{{\varvec{\Theta}}}_{{\varvec{\xi}}{\varvec{\xi}}}\left(f\right)\right\}$ (Eq. 18). Here, ${\varvec{\Omega}}\left(f\right)$ incorporates in ${{\varvec{\Theta}}}_{{\varvec{\iota}}{\varvec{\iota}}}^{-1}\left(f\right)$ similar information to the covariance-matrix ${\varvec{\Lambda}}\left(f\right)$ in a first step MAP1 (Eq. 2). In addition, ${\varvec{\Omega}}\left(f\right)$ is incorporated into ${{\varvec{\Theta}}}_{{\varvec{\xi}}{\varvec{\xi}}}^{-1}\left(f\right)$ information about the sensor noise covariance-matrix $\mathbf{\rm B}\left(f\right)$. This MAP1 represents a large class of inverse solutions distinguished by the methods determining ${\varvec{\Lambda}}\left(f\right)$ and $\mathbf{\rm B}\left(f\right)$ in the Bayesian literature^62,65–71^.

18 $$\begin{array}{l}\widehat{{\varvec{\iota}}}\left(f,t\right)={argmax}_{{\varvec{\iota}}\left(f,t\right)}\left\{p\left(\left.{\varvec{\iota}}\left(f,t\right)\right\vert {\varvec{v}}\left(t,f\right),{\varvec{\Omega}}\left(f\right)\right)\right\}\\ p\left(\left.{\varvec{\iota}}\left(f,t\right)\right\vert {\varvec{v}}\left(t,f\right),{\varvec{\Omega}}\left(f\right)\right)\propto {N}^{\mathbb{C}}\left({\varvec{v}}\left(t,f\right)\vert {\mathbf{L}}_{{\varvec{v}}{\varvec{\iota}}}{\varvec{\iota}}\left(t,f\right),{{\varvec{\Theta}}}_{{\varvec{\xi}}{\varvec{\xi}}}^{-1}\left(f\right)\right){N}^{\mathbb{C}}\left({\varvec{\iota}}\left(t,f\right)\vert 0,{{\varvec{\Theta}}}_{{\varvec{\iota}}{\varvec{\iota}}}^{-1}\left(f\right)\right)\end{array}$$

A HIGGS MAP1 estimate $\widehat{{\varvec{\iota}}}\left(f,t\right)$ (Eq. 18) is from a Gaussian a posteriori probability for ${\varvec{\iota}}\left(t,f\right)$ with mean value $\widehat{{\varvec{\iota}}}\left(f,t\right)$ and covariance-matrix ${{\varvec{\Pi}}}_{{\varvec{\iota}}{\varvec{\iota}}}\left(f\right)$ (Eq. 19). This MAP1 is a consequence of conjugated Gaussian relations between likelihood and a priori probability (Eq. 18), where estimate or mean value $\widehat{{\varvec{\iota}}}\left(f,t\right)$ is obtained through a quasilinear inverse operator ${\mathbf{T}}_{{\varvec{\iota}}{\varvec{v}}}\left(f\right)$ from data ${\varvec{v}}\left(t,f\right)$.

19 $$\begin{array}{l}\widehat{{\varvec{\iota}}}\left(f,t\right)={\mathbf{T}}_{{\varvec{\iota}}{\varvec{v}}}\left(f\right){\varvec{v}}\left(t,f\right)\\ p\left(\left.{\varvec{\iota}}\left(f,t\right)\right\vert {\varvec{v}}\left(t,f\right),{\varvec{\Omega}}\left(f\right)\right)={N}^{\mathbb{C}}\left({\varvec{\iota}}\left(t,f\right)\vert {\mathbf{T}}_{{\varvec{\iota}}{\varvec{v}}}\left(f\right){\varvec{v}}\left(t,f\right),{{\varvec{\Pi}}}_{{\varvec{\iota}}{\varvec{\iota}}}\left(f\right)\right)\end{array}$$

A quasilinear inverse operator ${\mathbf{T}}_{{\varvec{\iota}}{\varvec{v}}}\left(f\right)$ (Eq. 17) is the pseudoinverse for ${\mathbf{L}}_{{\varvec{v}}{\varvec{\iota}}}$ (forward-operator), as expressed in relation to a well-conditioned inverse or covariance-matrix ${{\varvec{\Pi}}}_{{\varvec{\iota}}{\varvec{\iota}}}\left(f\right)$. This pseudoinverse ${\mathbf{T}}_{{\varvec{\iota}}{\varvec{v}}}\left(f\right)$ is then a compact and efficient representation for estimating $\widehat{{\varvec{\iota}}}\left(f,t\right)$ (Eq. 16) from the data ${\varvec{v}}\left(t,f\right)$, which requires only a definition for parameters ${\varvec{\Omega}}\left(f\right)$.

20 $$\begin{array}{l}{\mathbf{T}}_{{\varvec{\iota}}{\varvec{v}}}\left(f\right)={{\varvec{\Pi}}}_{{\varvec{\iota}}{\varvec{\iota}}}\left(f\right){\mathbf{L}}_{{\varvec{\iota}}{\varvec{v}}}{{\varvec{\Theta}}}_{{\varvec{\xi}}{\varvec{\xi}}}\left(f\right)\\ {{\varvec{\Pi}}}_{{\varvec{\iota}}{\varvec{\iota}}}\left(f\right)={\left({\mathbf{L}}_{{\varvec{\iota}}{\varvec{v}}}{{\varvec{\Theta}}}_{{\varvec{\xi}}{\varvec{\xi}}}\left(f\right){\mathbf{L}}_{{\varvec{v}}{\varvec{\iota}}}+{{\varvec{\Theta}}}_{{\varvec{\iota}}{\varvec{\iota}}}\left(f\right)\right)}^{-1}\end{array}$$

This practice based on ${\mathbf{T}}_{{\varvec{\iota}}{\varvec{v}}}\left(f\right)$ (Eqs. 19 and 20) renders linear and stationary a whole process targeting brain oscillations ${\varvec{\iota}}\left(f,t\right)$, which avoids time-domain distortions^20,38,41,72^. While a theoretical ${\varvec{\iota}}\left(f,t\right)$ is converted by the forward-operator ${\mathbf{L}}_{{\varvec{v}}{\varvec{\iota}}}$ into data ${\varvec{v}}\left(t,f\right)$, these data are in turn converted by the pseudoinverse ${\mathbf{T}}_{{\varvec{\iota}}{\varvec{v}}}\left(f\right)$ into estimates $\widehat{{\varvec{\iota}}}\left(f,t\right)$. In other words, a MAP1 estimate (Eq. 19) is equivalent to estimating the sampled covariance matrix of brain oscillations ${\widehat{{\varvec{\Sigma}}}}_{{\varvec{\iota}}{\varvec{\iota}}}\left(f\right)$ directly by a linear matrix transformation ${\mathbf{T}}_{{\varvec{\iota}}{\varvec{v}}}\left(f\right)$ (Eq. 21) upon the data sampled covariance matrix ${\widehat{{\varvec{\Sigma}}}}_{{\varvec{v}}{\varvec{v}}}\left(f\right)$: where ${\widehat{{\varvec{\Sigma}}}}_{{\varvec{v}}{\varvec{v}}}\left(f\right)$ is determined for the oscillation data ${\varvec{v}}\left(t,f\right)$ at a specific frequency $f$ and within a transient $\forall t\in {\mathbb{T}}$ with sample size $T=\left\vert {\mathbb{T}}\right\vert$.

21 $$\begin{array}{l}{\widehat{{\varvec{\Sigma}}}}_{{\varvec{\iota}}{\varvec{\iota}}}\left(f\right)={\mathbf{T}}_{{\varvec{\iota}}{\varvec{v}}}\left(f\right){\widehat{{\varvec{\Sigma}}}}_{{\varvec{v}}{\varvec{v}}}\left(f\right){\mathbf{T}}_{{\varvec{v}}{\varvec{\iota}}}\left(f\right)\\ {\widehat{{\varvec{\Sigma}}}}_{{\varvec{v}}{\varvec{v}}}\left(f\right)=\frac{1}{T}{\sum }_{t\in {\mathbb{T}}}{\varvec{v}}\left(t,f\right){{\varvec{v}}\left(t,f\right)}^{\dagger}\end{array}$$

In state-of-the-art practice, such a definition for ${\varvec{\Omega}}\left(f\right)$ is most commonly through two methods: *Exact Low Resolution Electromagnetic Tomographic Analysis* (**ELORETA**)^56^ and *Linearly Constrained Minimum Variance* (**LCMV**)^55^. These methods then determine ${\varvec{\Omega}}\left(f\right)$ by some optimal criteria for brain oscillations $\widehat{{\varvec{\iota}}}\left(f,t\right)$ explaining the sampled covariance matrix ${\widehat{{\varvec{\Sigma}}}}_{{\varvec{v}}{\varvec{v}}}\left(f\right)$ (Eq. 21)^20,44^.

Elsewhere, this type of inverse operator ${\mathbf{T}}_{{\varvec{v}}{\varvec{\iota}}}\left(f\right)$ may also impose sparsity to identify nonzero components in the vector ${\varvec{\iota}}\left(t,f\right)$ and be obtained iteratively along with ${\varvec{\Omega}}\left(f\right)$ through Bayesian hierarchical methods^62,65–71^ or proximal projection methods^109–116^. Additionally, elsewhere, an inverse operator that is nonlinear ${\mathbf{T}}_{{\varvec{\iota}}{\varvec{v}}}\left({\varvec{v}}\left(t,f\right)\right)$, quasilinear nonstationary ${\mathbf{T}}_{{\varvec{\iota}}{\varvec{v}}}\left(t,f\right)$, or both nonlinear and nonstationary ${\mathbf{T}}_{{\varvec{\iota}}{\varvec{v}}}\left(t,f,{\varvec{v}}\left(t,f\right)\right)$ is preferable to identify the type of latent brain activity causing evoked potentials in MEG/EEG^117,118^.

#### HIGGS model MAP2 inverse solution for functional connectivity via successive approximations of MAP1 inverse solution

Assume a HIGGS model (Eq. 17) explaining the MEG/EEG oscillation data ${\varvec{v}}\left(t,f\right)$ upon ${\varvec{\iota}}\left(t,f\right)$. Then, marginalizing brain-oscillations ${\varvec{\iota}}\left(t,f\right)$ for the likelihood $p\left(\left.{\varvec{v}}\left(t,f\right)\right\vert {\varvec{\iota}}\left(t,f\right),{{\varvec{\Theta}}}_{{\varvec{\xi}}{\varvec{\xi}}}\left(f\right)\right)$ and under the a priori GGS model $p\left(\left.{\varvec{\iota}}\left(t,f\right)\right\vert {{\varvec{\Theta}}}_{{\varvec{\iota}}{\varvec{\iota}}}\left(f\right)\right)$ translates into another GGS model (Eq. 22). This GGS model explains oscillations data ${\varvec{v}}\left(t,f\right)$ upon a precision-matrix ${{\varvec{\Theta}}}_{{\varvec{v}}{\varvec{v}}}$ dependent on hyperparameters ${\varvec{\Omega}}\left(f\right)=\left\{{{\varvec{\Theta}}}_{{\varvec{\iota}}{\varvec{\iota}}}\left(f\right),{{\varvec{\Theta}}}_{{\varvec{\xi}}{\varvec{\xi}}}\left(f\right)\right\}$.

22 $$\begin{array}{l}p\left(\left.{\varvec{v}}\left(t,f\right)\right\vert {\varvec{\Omega}}\left(f\right)\right)={N}^{\mathbb{C}}\left({\varvec{v}}\left(t,f\right)\vert 0,{{\varvec{\Theta}}}_{{\varvec{v}}{\varvec{v}}}^{-1}\left(f\right)\right)\\ {{\varvec{\Theta}}}_{{\varvec{v}}{\varvec{v}}}^{-1}\left(f\right)={\mathbf{L}}_{{\varvec{v}}{\varvec{\iota}}}{{\varvec{\Theta}}}_{{\varvec{\iota}}{\varvec{\iota}}}^{-1}\left(f\right){\mathbf{L}}_{{\varvec{\iota}}{\varvec{v}}}+{{\varvec{\Theta}}}_{{\varvec{\xi}}{\varvec{\xi}}}^{-1}\left(f\right)\end{array}$$

where estimating a precision matrix ${{\varvec{\Theta}}}_{{\varvec{v}}{\varvec{v}}}\left(f\right)$ in this GGS model (Eq. 22) may also be regarded as a pseudoinverse for the sampled covariance matrix ${\widehat{{\varvec{\Sigma}}}}_{{\varvec{v}}{\varvec{v}}}\left(f\right)$ (Eq. 23) for several samples ($\forall t\in {\mathbb{T}}$), where the sample size $T=\left\vert {\mathbb{T}}\right\vert$.

23 $${\widehat{{\varvec{\Sigma}}}}_{{\varvec{v}}{\varvec{v}}}\left(f\right)=\frac{1}{T}{\sum }_{t\in {\mathbb{T}}}{\varvec{v}}\left(t,f\right){{\varvec{v}}\left(t,f\right)}^{\dagger}$$

However, estimating the precision matrix ${{\varvec{\Theta}}}_{{\varvec{\iota}}{\varvec{\iota}}}\left(f\right)$ in this GGS model is twice a pseudoinverse, first for the sampled covariance matrix ${\widehat{{\varvec{\Sigma}}}}_{{\varvec{v}}{\varvec{v}}}\left(f\right)$ and second the forward operator ${\mathbf{L}}_{{\varvec{v}}{\varvec{\iota}}}$ (Eq. 22), also involving estimation of the precision matrix ${{\varvec{\Theta}}}_{{\varvec{\xi}}{\varvec{\xi}}}\left(f\right)$. The second-type likelihood explaining the sampled covariance-matrix ${\widehat{{\varvec{\Sigma}}}}_{{\varvec{v}}{\varvec{v}}}\left(f\right)$ (Eq. 23) is a complex-valued Wishart (Eq. 24), with degree of freedom $T$ and scale matrix ${T}^{-1}{{\varvec{\Theta}}}_{{\varvec{v}}{\varvec{v}}}^{-1}\left(f\right)$. With this second-type likelihood (Eq. 24), direct estimation of the precision-matrices or hyperparameters ${\varvec{\Omega}}\left(f\right)=\left\{{{\varvec{\Theta}}}_{{\varvec{\iota}}{\varvec{\iota}}}\left(f\right),{{\varvec{\Theta}}}_{{\varvec{\xi}}{\varvec{\xi}}}\left(f\right)\right\}$ is impractical, requiring variational Bayes approximations or Gibbs sampling not extensible to high dimensions.

24 $$\begin{array}{l}p\left(\left.{\widehat{{\varvec{\Sigma}}}}_{{\varvec{v}}{\varvec{v}}}\left(f\right)\right\vert {\varvec{\Omega}}\left(f\right)\right)={W}^{\mathbb{C}}\left({\widehat{{\varvec{\Sigma}}}}_{{\varvec{\iota}}{\varvec{\iota}}}\left(f\right)\vert {T}^{-1}\left({\mathbf{L}}_{{\varvec{v}}{\varvec{\iota}}}{{\varvec{\Theta}}}_{{\varvec{\iota}}{\varvec{\iota}}}^{-1}\left(f\right){\mathbf{L}}_{{\varvec{\iota}}{\varvec{v}}}+{{\varvec{\Theta}}}_{{\varvec{\xi}}{\varvec{\xi}}}^{-1}\left(f\right)\right),T\right)\\ p\left({\varvec{\Omega}}\left(f\right)\right)=p\left({{\varvec{\Theta}}}_{{\varvec{\iota}}{\varvec{\iota}}}\left(f\right)\right)p\left({{\varvec{\Theta}}}_{{\varvec{\xi}}{\varvec{\xi}}}\left(f\right)\right)\end{array}$$

Therefore, we use the successive approximations to the second-type likelihood $p\left(\left.{\widehat{{\varvec{\Sigma}}}}_{{\varvec{v}}{\varvec{v}}}\left(f\right)\right\vert {\varvec{\Omega}}\left(f\right)\right)$ (Eq. 24) by the Expectation Maximization (EM) algorithm^66,73,88,89,119,120^. These successive approximations at an EM $k$-th iteration are expressed in Gibbs exponential form ${p}^{\left(k\right)}\left({\widehat{{\varvec{\Sigma}}}}_{{\varvec{v}}{\varvec{v}}}\left(f\right)\vert {\varvec{\Omega}}\left(f\right)\right)$ of an expected $-log$ second-type likelihood of the data sampled covariance-matrix ${\widehat{{\varvec{\Sigma}}}}_{{\varvec{v}}{\varvec{v}}}\left(f\right)$ implicit in the cost function $Q\left({\varvec{\Omega}}\left(f\right),{\widehat{{\varvec{\Omega}}}}^{\left(k\right)}\left(f\right)\right)$ (Eq. 25).

25 $${p}^{\left(k\right)}\left({\widehat{{\varvec{\Sigma}}}}_{{\varvec{v}}{\varvec{v}}}\left(f\right)\vert {\varvec{\Omega}}\left(f\right)\right)=exp\left(\left.Q\left({\varvec{\Omega}}\left(f\right),{\widehat{{\varvec{\Omega}}}}^{\left(k\right)}\left(f\right)\right)\right\vert 1\right)$$

where the cost function $Q\left({\varvec{\Omega}}\left(f\right),{\widehat{{\varvec{\Omega}}}}^{\left(k\right)}\left(f\right)\right)$ is obtained from the expected $-log$ second-type likelihood (Eq. 26) of the data ${\varvec{v}}\left(t,f\right)$ for several samples ($\forall t\in {\mathbb{T}}$) and upon hyperparameters ${\varvec{\Omega}}\left(f\right)$ and hyperparameters from the previous EM iteration ${\widehat{{\varvec{\Omega}}}}^{\left(k\right)}\left(f\right)$.

26 $$Q\left({\varvec{\Omega}}\left(f\right),{\widehat{{\varvec{\Omega}}}}^{\left(k\right)}\left(f\right)\right)=-{\sum }_{t\in {\mathbb{T}}}E\left\{\left.log\left(p\left({\varvec{v}}\left(t,f\right),{\varvec{\iota}}\left(t,f\right)\vert {\varvec{\Omega}}\left(f\right)\right)\right)\right\vert {\widehat{{\varvec{\Omega}}}}^{\left(k\right)}\left(f\right)\right\}$$

This likelihood is obtained from expectation applied to the $-log$ joint probability $p\left({\varvec{v}}\left(t,f\right),{\varvec{\iota}}\left(t,f\right)\vert {\varvec{\Omega}}\left(f\right)\right)$ over parameters (brain oscillations) ${\varvec{\iota}}\left(t,f\right)$ (Eq. 27). The joint probability is composed of the HIGGS model first-type likelihood and a priori probability (Eq. 27), and the expectation is based on the a posteriori probability of the HIGGS MAP1 (Eq. 19).

27 $$\begin{array}{l}E\left\{\left.log\left(p\left({\varvec{v}}\left(t,f\right),{\varvec{\iota}}\left(t,f\right)\vert {\varvec{\Omega}}\left(f\right)\right)\right)\right\vert {\widehat{{\varvec{\Omega}}}}^{\left(k\right)}\left(f\right)\right\}=\int log\left(p\left({\varvec{v}}\left(t,f\right),{\varvec{\iota}}\left(t,f\right)\vert {\varvec{\Omega}}\left(f\right)\right)\right)p\left({\varvec{\iota}}\left(t,f\right)\vert {\varvec{v}}\left(t,f\right),{\widehat{{\varvec{\Omega}}}}^{\left(k\right)}\left(f\right)\right)d{\varvec{\iota}}\left(t,f\right)\\ \begin{array}{l}p\left({\varvec{v}}\left(t,f\right),{\varvec{\iota}}\left(t,f\right)\vert {\varvec{\Omega}}\left(f\right)\right)={N}^{\mathbb{C}}\left({\varvec{v}}\left(t,f\right)\vert {\mathbf{L}}_{{\varvec{v}}{\varvec{\iota}}}{\varvec{\iota}}\left(t,f\right),{{\varvec{\Theta}}}_{{\varvec{\xi}}{\varvec{\xi}}}^{-1}\left(f\right)\right){N}^{\mathbb{C}}\left({\varvec{\iota}}\left(t,f\right)\vert 0,{{\varvec{\Theta}}}_{{\varvec{\iota}}{\varvec{\iota}}}^{-1}\left(f\right)\right)\\ p\left(\left.{\varvec{\iota}}\left(f,t\right)\right\vert {\varvec{v}}\left(t,f\right),{\widehat{{\varvec{\Omega}}}}^{\left(k\right)}\left(f\right)\right)={N}^{\mathbb{C}}\left({\varvec{\iota}}\left(t,f\right)\vert {\mathbf{T}}_{{\varvec{\iota}}{\varvec{v}}}^{\left(k\right)}\left(f\right){\varvec{v}}\left(t,f\right),{{\varvec{\Pi}}}_{{\varvec{\iota}}{\varvec{\iota}}}^{\left(k\right)}\left(f\right)\right)\end{array}\end{array}$$

The function $Q\left({\varvec{\Omega}}\left(f\right),{\widehat{{\varvec{\Omega}}}}^{\left(k\right)}\left(f\right)\right)$ possesses an additive form (Eq. 28) upon expected covariance-matrices for parameters (brain-oscillations) ${\widehat{{\varvec{\Psi}}}}_{{\varvec{\iota}}{\varvec{\iota}}}^{\left(k\right)}\left(f\right)$ and residuals (spectral sensor noise process) ${\widehat{{\varvec{\Psi}}}}_{{\varvec{\xi}}{\varvec{\xi}}}^{\left(k\right)}\left(f\right)$. These expected covariance matrices are dependent on the data sampled covariance ${\widehat{{\varvec{\Sigma}}}}_{{\varvec{v}}{\varvec{v}}}\left(f\right)$ hyperparameters ${\widehat{{\varvec{\Omega}}}}^{\left(k\right)}\left(f\right)$ at the $k$-th EM expectation.

28 $$\begin{array}{l}Q\left({\varvec{\Omega}}\left(f\right),{\widehat{{\varvec{\Omega}}}}^{\left(k\right)}\left(f\right)\right)=Q\left({{\varvec{\Theta}}}_{{\varvec{\iota}}{\varvec{\iota}}}\left(f\right),{\widehat{{\varvec{\Psi}}}}_{{\varvec{\iota}}{\varvec{\iota}}}^{\left(k\right)}\left(f\right)\right)+Q\left({{\varvec{\Theta}}}_{{\varvec{\xi}}{\varvec{\xi}}}\left(f\right),{\widehat{{\varvec{\Psi}}}}_{{\varvec{\xi}}{\varvec{\xi}}}^{\left(k\right)}\left(f\right)\right)\\ \begin{array}{l}Q\left({{\varvec{\Theta}}}_{{\varvec{\iota}}{\varvec{\iota}}}\left(f\right),{\widehat{{\varvec{\Psi}}}}_{{\varvec{\iota}}{\varvec{\iota}}}^{\left(k\right)}\left(f\right)\right)=-Tlog\left\vert {{\varvec{\Theta}}}_{{\varvec{\iota}}{\varvec{\iota}}}\left(f\right)\right\vert +Ttr\left({\stackrel{\smile}{{\varvec{\Psi}}}}_{{\varvec{\iota}}{\varvec{\iota}}}^{\left(k\right)}\left(f\right){{\varvec{\Theta}}}_{{\varvec{\iota}}{\varvec{\iota}}}\left(f\right)\right)\\ Q\left({{\varvec{\Theta}}}_{{\varvec{\xi}}{\varvec{\xi}}}\left(f\right),{\widehat{{\varvec{\Psi}}}}_{{\varvec{\xi}}{\varvec{\xi}}}^{\left(k\right)}\left(f\right)\right)=-Tlog\left\vert {{\varvec{\Theta}}}_{{\varvec{\xi}}{\varvec{\xi}}}\left(f\right)\right\vert +Ttr\left({\widehat{{\varvec{\Psi}}}}_{{\varvec{\xi}}{\varvec{\xi}}}^{\left(k\right)}\left(f\right){{\varvec{\Theta}}}_{{\varvec{\xi}}{\varvec{\xi}}}\left(f\right)\right)\end{array}\end{array}$$

The expected covariance-matrix ${\widehat{{\varvec{\Psi}}}}_{{\varvec{\iota}}{\varvec{\iota}}}^{\left(k\right)}\left(f\right)$ (Eq. 29) is determined from an ensemble covariance-matrix ${\widehat{{\varvec{\Pi}}}}_{{\varvec{\iota}}{\varvec{\iota}}}^{\left(k\right)}\left(f\right)$ and its sampled estimator ${\widehat{{\varvec{\Sigma}}}}_{{\varvec{\iota}}{\varvec{\iota}}}^{\left(k\right)}\left(f\right)$ due to the a posteriori probability $p\left(\left.{\varvec{\iota}}\left(f,t\right)\right\vert {\varvec{v}}\left(t,f\right),{\widehat{{\varvec{\Omega}}}}^{\left(k\right)}\left(f\right)\right)$ (Eq. 27)^89^.

29 $$\begin{array}{l}{\widehat{{\varvec{\Psi}}}}_{{\varvec{\iota}}{\varvec{\iota}}}^{\left(k\right)}\left(f\right)={\widehat{{\varvec{\Sigma}}}}_{{\varvec{\iota}}{\varvec{\iota}}}^{\left(k\right)}\left(f\right)+{\widehat{{\varvec{\Pi}}}}_{{\varvec{\iota}}{\varvec{\iota}}}^{\left(k\right)}\left(f\right)\\ {\widehat{{\varvec{\Sigma}}}}_{{\varvec{\iota}}{\varvec{\iota}}}^{\left(k\right)}\left(f\right)={\mathbf{T}}_{{\varvec{\iota}}{\varvec{v}}}^{\left(k\right)}\left(f\right){\widehat{{\varvec{\Sigma}}}}_{{\varvec{v}}{\varvec{v}}}\left(f\right){\mathbf{T}}_{{\varvec{v}}{\varvec{\iota}}}^{\left(k\right)}\left(f\right)\\ \begin{array}{l}{\mathbf{T}}_{{\varvec{\iota}}{\varvec{v}}}^{\left(k\right)}\left(f\right)={\widehat{{\varvec{\Pi}}}}_{{\varvec{\iota}}{\varvec{\iota}}}^{\left(k\right)}\left(f\right){\mathbf{L}}_{{\varvec{\iota}}{\varvec{v}}}{\widehat{{\varvec{\Theta}}}}_{{\varvec{\xi}}{\varvec{\xi}}}^{\left(k\right)}\left(f\right)\\ {\widehat{{\varvec{\Pi}}}}_{{\varvec{\iota}}{\varvec{\iota}}}^{\left(k\right)}\left(f\right)={\left({\mathbf{L}}_{{\varvec{\iota}}{\varvec{v}}}{\widehat{{\varvec{\Theta}}}}_{{\varvec{\xi}}{\varvec{\xi}}}^{\left(k\right)}{\mathbf{L}}_{{\varvec{v}}{\varvec{\iota}}}+{\widehat{{\varvec{\Theta}}}}_{{\varvec{\iota}}{\varvec{\iota}}}^{\left(k\right)}\right)}^{-1}\end{array}\end{array}$$

Analogous formulas are obtained for an expected covariance-matrix ${\widehat{{\varvec{\Psi}}}}_{{\varvec{\xi}}{\varvec{\xi}}}^{\left(k\right)}\left(f\right)$ (Eq. 30) determined from an ensemble covariance-matrix ${\widehat{{\varvec{\Pi}}}}_{{\varvec{\xi}}{\varvec{\xi}}}^{\left(k\right)}\left(f\right)$ and its sampled estimator ${\widehat{{\varvec{\Sigma}}}}_{{\varvec{\iota}}{\varvec{\iota}}}^{\left(k\right)}\left(f\right)$ in this case due to the a posteriori probability for the sensor noise process $p\left(\left.{\varvec{\xi}}\left(f,t\right)\right\vert {\varvec{v}}\left(t,f\right),{\widehat{{\varvec{\Omega}}}}^{\left(k\right)}\left(f\right)\right)$^89^.

30 $$\begin{array}{l}{\widehat{{\varvec{\Psi}}}}_{{\varvec{\xi}}{\varvec{\xi}}}^{\left(k\right)}\left(f\right)={\widehat{{\varvec{\Sigma}}}}_{{\varvec{\xi}}{\varvec{\xi}}}^{\left(k\right)}\left(f\right)+{\widehat{{\varvec{\Pi}}}}_{{\varvec{\xi}}{\varvec{\xi}}}^{\left(k\right)}\left(f\right)\\ {\widehat{{\varvec{\Sigma}}}}_{{\varvec{\xi}}{\varvec{\xi}}}^{\left(k\right)}\left(f\right)={\mathbf{T}}_{{\varvec{\xi}}{\varvec{v}}}^{\left(k\right)}\left(f\right){\widehat{{\varvec{\Sigma}}}}_{{\varvec{v}}{\varvec{v}}}\left(f\right){\mathbf{T}}_{{\varvec{\xi}}{\varvec{\iota}}}^{\left(k\right)}\left(f\right)\\ \begin{array}{l}{\mathbf{T}}_{{\varvec{\xi}}{\varvec{v}}}^{\left(k\right)}\left(f\right)=\mathbf{I}-{\widehat{{\varvec{\Pi}}}}_{{\varvec{\xi}}{\varvec{\xi}}}^{\left(k\right)}\left(f\right){\widehat{{\varvec{\Theta}}}}_{{\varvec{\xi}}{\varvec{\xi}}}^{\left(k\right)}\left(f\right)\\ {\widehat{{\varvec{\Pi}}}}_{{\varvec{\xi}}{\varvec{\xi}}}^{\left(k\right)}\left(f\right)={\mathbf{L}}_{{\varvec{v}}{\varvec{\iota}}}{\widehat{{\varvec{\Pi}}}}_{{\varvec{\iota}}{\varvec{\iota}}}^{\left(k\right)}\left(f\right){\mathbf{L}}_{{\varvec{\iota}}{\varvec{v}}}\end{array}\end{array}$$

Then, due to the cost function additive form (Eq. 28), the Gibbs exponential form ${p}^{\left(k\right)}\left({\widehat{{\varvec{\Sigma}}}}_{{\varvec{v}}{\varvec{v}}}\left(f\right)\vert {\varvec{\Omega}}\left(f\right)\right)$ (Eq. 25) admits a factorization into two marginal second-type Wishart (${W}^{\mathbb{C}}$) likelihood models (Eq. 31). Se details the derivation for these successive approximations to the HIGGS second-type likelihood in SI III. Bimodal Wishart form of the HIGGS expected second-type likelihood (Lemma 1).

31 $$\begin{array}{l}{p}^{\left(k\right)}\left({\widehat{{\varvec{\Sigma}}}}_{{\varvec{v}}{\varvec{v}}}\left(f\right)\vert {\varvec{\Omega}}\left(f\right)\right)={p}^{\left(k\right)}\left(\left.{\widehat{{\varvec{\Sigma}}}}_{{\varvec{v}}{\varvec{v}}}\left(f\right)\right\vert {{\varvec{\Theta}}}_{{\varvec{\iota}}{\varvec{\iota}}}\left(f\right)\right){p}^{\left(k\right)}\left(\left.{\widehat{{\varvec{\Sigma}}}}_{{\varvec{v}}{\varvec{v}}}\left(f\right)\right\vert {{\varvec{\Theta}}}_{{\varvec{\xi}}{\varvec{\xi}}}\left(f\right)\right)\\ {p}^{\left(k\right)}\left(\left.{\widehat{{\varvec{\Sigma}}}}_{{\varvec{v}}{\varvec{v}}}\left(f\right)\right\vert {{\varvec{\Theta}}}_{{\varvec{\iota}}{\varvec{\iota}}}\left(f\right)\right)={W}^{\mathbb{C}}\left({\widehat{{\varvec{\Psi}}}}_{{\varvec{\iota}}{\varvec{\iota}}}^{\left(k\right)}\left(f\right)\vert {T}^{-1}{{\varvec{\Theta}}}_{{\varvec{\iota}}{\varvec{\iota}}}^{-1}\left(f\right),T\right)\\ {p}^{\left(k\right)}\left(\left.{\widehat{{\varvec{\Sigma}}}}_{{\varvec{v}}{\varvec{v}}}\left(f\right)\right\vert {{\varvec{\Theta}}}_{{\varvec{\xi}}{\varvec{\xi}}}\left(f\right)\right)={W}^{\mathbb{C}}\left({\widehat{{\varvec{\Psi}}}}_{{\varvec{\xi}}{\varvec{\xi}}}^{\left(k\right)}\left(f\right)\vert {T}^{-1}{{\varvec{\Theta}}}_{{\varvec{\xi}}{\varvec{\xi}}}^{-1}\left(f\right),T\right)\end{array}$$

The EM approximated MAP2 (maximization stage) ${\widehat{{\varvec{\Theta}}}}_{{\varvec{\iota}}{\varvec{\iota}}}^{\left(k+1\right)}\left(f\right)$ and ${\widehat{{\varvec{\Theta}}}}_{{\varvec{\xi}}{\varvec{\xi}}}^{\left(k+1\right)}\left(f\right)$ (Eq. 32) may be trapped at local optima but is explicitly Wishart and scalable to high dimensions combined with regularization priors $p\left({{\varvec{\Theta}}}_{{\varvec{\iota}}{\varvec{\iota}}}\left(f\right)\right)$ and $p\left({{\varvec{\Theta}}}_{{\varvec{\xi}}{\varvec{\xi}}}\left(f\right)\right)$ that may facilitate the search closest to the global optimal^88,120^. As with the previous first-type likelihood for ${{\varvec{\Theta}}}_{{\varvec{\iota}}{\varvec{\iota}}}\left(f\right)$ (Eqs. 13, 14, 15), any of the penalizations discussed in the previous section may be used here to regularize the EM procedure. See Lemma 1 in SI IV. HIGGS second-type maximum a posteriori and priors (Corollary to Lemma 1).

32 $$\begin{array}{l}\begin{array}{l}{\widehat{{\varvec{\Theta}}}}_{{\varvec{\iota}}{\varvec{\iota}}}^{\left(k+1\right)}\left(f\right)={argmax}_{{{\varvec{\Theta}}}_{{\varvec{\iota}}{\varvec{\iota}}}\left(f\right)}\left\{{p}^{\left(k\right)}\left(\left.{{\varvec{\Theta}}}_{{\varvec{\iota}}{\varvec{\iota}}}\left(f\right)\right\vert {\widehat{{\varvec{\Sigma}}}}_{{\varvec{v}}{\varvec{v}}}\left(f\right)\right)\right\}\\ {\widehat{{\varvec{\Theta}}}}_{{\varvec{\xi}}{\varvec{\xi}}}^{\left(k+1\right)}\left(f\right)={argmax}_{{{\varvec{\Theta}}}_{{\varvec{\xi}}{\varvec{\xi}}}\left(f\right)}\left\{{p}^{\left(k\right)}\left(\left.{{\varvec{\Theta}}}_{{\varvec{\xi}}{\varvec{\xi}}}\left(f\right)\right\vert {\widehat{{\varvec{\Sigma}}}}_{{\varvec{v}}{\varvec{v}}}\left(f\right)\right)\right\}\end{array}\\ {p}^{\left(k\right)}\left(\left.{{\varvec{\Theta}}}_{{\varvec{\iota}}{\varvec{\iota}}}\left(f\right)\right\vert {\widehat{{\varvec{\Sigma}}}}_{{\varvec{v}}{\varvec{v}}}\left(f\right)\right)={W}^{\mathbb{C}}\left({\widehat{{\varvec{\Psi}}}}_{{\varvec{\iota}}{\varvec{\iota}}}^{\left(k\right)}\left(f\right)\vert {T}^{-1}{{\varvec{\Theta}}}_{{\varvec{\iota}}{\varvec{\iota}}}^{-1}\left(f\right),T\right)p\left({{\varvec{\Theta}}}_{{\varvec{\iota}}{\varvec{\iota}}}\left(f\right)\right)\\ {p}^{\left(k\right)}\left(\left.{{\varvec{\Theta}}}_{{\varvec{\xi}}{\varvec{\xi}}}\left(f\right)\right\vert {\widehat{{\varvec{\Sigma}}}}_{{\varvec{v}}{\varvec{v}}}\left(f\right)\right)={W}^{\mathbb{C}}\left({\widehat{{\varvec{\Psi}}}}_{{\varvec{\xi}}{\varvec{\xi}}}^{\left(k\right)}\left(f\right)\vert {T}^{-1}{{\varvec{\Theta}}}_{{\varvec{\xi}}{\varvec{\xi}}}^{-1}\left(f\right),T\right)p\left({{\varvec{\Theta}}}_{{\varvec{\xi}}{\varvec{\xi}}}\left(f\right)\right)\end{array}$$

The EM maximization stage (Eq. 32) poses the same formalism as for a pair of observed GGS models at every iteration based on the $-log$ transformation of this posterior distribution, an additive penalized cost function ${\mathcal{L}}^{\left(k\right)}\left({\varvec{\Omega}}\left(f\right)\right)={\mathcal{L}}^{\left(k\right)}\left({{\varvec{\Theta}}}_{{\varvec{\iota}}{\varvec{\iota}}}\left(f\right)\right)+{\mathcal{L}}^{\left(k\right)}\left({{\varvec{\Theta}}}_{{\varvec{\xi}}{\varvec{\xi}}}\left(f\right)\right)$. Note that for expected covariance-matrix ${\widehat{{\varvec{\Psi}}}}_{{\varvec{\iota}}{\varvec{\iota}}}^{\left(k\right)}\left(f\right)$ (Eq. 33), which is not directly observable, this is the same problem as for the observed ${\widehat{{\varvec{\Sigma}}}}_{{\varvec{\iota}}{\varvec{\iota}}}\left(f\right)$ (Eqs 13, 14, 15), employing the graphical a priori models $\mathrm{P}$. A novel algorithm exposed here computes this solution based on the Bayesian hierarchical **hgLASSO** a priori model 2.3 GGS model MAP1 via Hermitian Graphical LASSO (hgLASSO).

33 $$\begin{array}{l}{\widehat{{\varvec{\Theta}}}}_{{\varvec{\iota}}{\varvec{\iota}}}^{\left(k+1\right)}\left(f\right)={argmin}_{{{\varvec{\Theta}}}_{{\varvec{\iota}}{\varvec{\iota}}}\left(f\right)}\left\{{\mathcal{L}}^{\left(k\right)}\left({{\varvec{\Theta}}}_{{\varvec{\iota}}{\varvec{\iota}}}\left(f\right)\right)\right\}\\ {\mathcal{L}}^{\left(k\right)}\left({{\varvec{\Theta}}}_{{\varvec{\iota}}{\varvec{\iota}}}\left(f\right)\right)=-log\left\vert {{\varvec{\Theta}}}_{{\varvec{\iota}}{\varvec{\iota}}}\left(f\right)\right\vert +tr\left({\stackrel{\smile}{{\varvec{\Psi}}}}_{{\varvec{\iota}}{\varvec{\iota}}}^{\left(k\right)}\left(f\right){{\varvec{\Theta}}}_{{\varvec{\iota}}{\varvec{\iota}}}\left(f\right)\right)+{\alpha }_{{\varvec{\iota}}}\mathrm{P}\left({\mathbf{A}}_{{\varvec{\iota}}{\varvec{\iota}}}\odot {{\varvec{\Theta}}}_{{\varvec{\iota}}{\varvec{\iota}}}\left(f\right)\right)\end{array}$$

For the spectral sensor noise process, we assume that ${{\varvec{\Theta}}}_{{\varvec{\xi}}{\varvec{\xi}}}\left(f\right)$ is known but a scalar factor ${\theta }_{{\varvec{\xi}}}^{2}\left(f\right)$ to be estimated: ${{\varvec{\Theta}}}_{{\varvec{\xi}}{\varvec{\xi}}}\left(f\right)={\theta }_{{\varvec{\xi}}}^{2}\left(f\right){\mathbf{A}}_{{\varvec{\xi}}{\varvec{\xi}}}$, for which we use an exponential prior with scale parameter ${\alpha }_{{\varvec{\xi}}}$ (Eq. 34), with $\mathrm{p}$ being the number of MEG/EEG sensors and $T$ being the sample number.

34 $$p\left( {\theta _\xi ^2\left( f \right)\left| {{\alpha _\xi }} \right.} \right) = exp\left( {\theta _\xi ^2\left( f \right)|{\alpha _\xi }{\text{p}}T} \right)$$

In this paper, we define ${\mathbf{A}}_{{\varvec{\xi}}{\varvec{\xi}}}$ as the identity matrix but note that it might be used to encode spurious EEG sensor connectivity due to scalp leakage currents. ${\alpha }_{{\varvec{\xi}}}$ (Eq. 34) could be used to encode the instrumental noise inferior threshold that we define as 10% of the EEG signal (Eq. 35).

35 $$\begin{array}{l} {\hat \theta {{_\xi ^2}^{(k + 1)}}\left( f \right) = 1/\left( {tr\left( {{\stackrel{\smile}{{\varvec{\Psi}}}} _{\xi \xi }^{\left( k \right)}\left( f \right){{\bf{A}}_{\xi \xi }}} \right)/{\text{p}} + {\alpha _\xi }} \right)} \hfill \\ {{{\mathcal L}^{\left( k \right)}}\left( {\theta _\xi ^2\left( f \right)} \right) = - {\text{p}}log\left( {\theta _\xi ^2\left( f \right)} \right) + \theta _\xi ^2\left( f \right)tr\left( {{\stackrel{\smile}{{\varvec{\Psi}}}} _{\xi \xi }^{\left( k \right)}\left( f \right){{\bf{A}}_{\xi \xi }}} \right) + {\alpha _\xi }{\text{p}}\theta _\xi ^2} \hfill \\ \end{array}$$

### GGS model MAP1 inverse solution via Hermitian Graphical LASSO (hgLASSO) algorithm

#### Unbiasedness of the MAP1 inverse solution with hgLASSO a priori probability

We leverage recent results on the distribution of high-dimensional estimators of precision matrices of Jankova and Van de Geer (JVDG)^82^ to provide statistical guarantees for the HIGGS inverse solution. First, we reduce the bias of ${\widehat{{\varvec{\Theta}}}}_{{\varvec{\iota}}{\varvec{\iota}}}\left(f\right)$ at each iteration of EM maximization in the **onestep hgLASSO** inverse solution (Eq. 18) or second step in the multistep **hgLASSO** inverse solution (Eq. 13) by substituting ${\stackrel{\smile}{{\varvec{\Psi}}}}_{{\varvec{\iota}}{\varvec{\iota}}}^{\left(k\right)}\left(f\right)$ in ${\widehat{{\varvec{\Sigma}}}}_{{\varvec{\iota}}{\varvec{\iota}}}\left(f\right)$, an unbiased estimator via “desparsification” (Eq. 21). This unbiased estimator is the complex-valued extension for the graphical LASSO (gLASSO)^35^ exposed in the JVDG theory^82^.

36 $$unb\left({\widehat{{\varvec{\Theta}}}}_{{\varvec{\iota}}{\varvec{\iota}}}\left(f\right)\right)=2{\widehat{{\varvec{\Theta}}}}_{{\varvec{\iota}}{\varvec{\iota}}}\left(f\right)-{\widehat{{\varvec{\Theta}}}}_{{\varvec{\iota}}{\varvec{\iota}}}\left(f\right){\widehat{{\varvec{\Sigma}}}}_{{\varvec{\iota}}{\varvec{\iota}}}\left(f\right){\widehat{{\varvec{\Theta}}}}_{{\varvec{\iota}}{\varvec{\iota}}}\left(f\right)$$

Debiasing ${\widehat{{\varvec{\Theta}}}}_{{\varvec{\iota}}{\varvec{\iota}}}\left(f\right)$ is particularly relevant to increase the reliability of functional connectivity estimates and reduce distortions. This unbiased estimator $unb\left({\widehat{{\varvec{\Theta}}}}_{{\varvec{\iota}}{\varvec{\iota}}}\left(f\right)\right)$ follows the Hermitian Gaussian distribution (Eq. 22), allowing us to carry the thresholding of the EM maximization in the **onestep hgLASSO** inverse solution (Eq. 18) or the second step in the multistep **hgLASSO** inverse solution (Eq. 13).

37 $$\begin{array}{l}p\left(unb\left({\widehat{{\varvec{\Theta}}}}_{{\varvec{\iota}}{\varvec{\iota}}}\left(f;i,j\right)\right)\right)={N}_{1}^{\mathbb{C}}\left(unb\left({\widehat{{\varvec{\Theta}}}}_{{\varvec{\iota}}{\varvec{\iota}}}\left(f;i,j\right)\right)\vert {{\varvec{\Theta}}}_{{\varvec{\iota}}{\varvec{\iota}}}\left(f;i,j\right),{\widehat{{\varvec{\Sigma}}}}_{{\varvec{\Theta}}}\left(f;i,j\right)/\sqrt{T}\right)\\ {\widehat{{\varvec{\Sigma}}}}_{{\varvec{\Theta}}}\left(f;i,j\right)={\widehat{{\varvec{\Theta}}}}_{{\varvec{\iota}}{\varvec{\iota}}}\left(f;i,i\right){\widehat{{\varvec{\Theta}}}}_{{\varvec{\iota}}{\varvec{\iota}}}\left(f;j,j\right)+{\widehat{{\varvec{\Theta}}}}_{{\varvec{\iota}}{\varvec{\iota}}}\left(f;i,j\right)\end{array}$$

With the fixed value of the regularization parameter ${\alpha}_{{\boldsymbol{\iota}}}=\sqrt{T \, log\, \left({\rm q}\right)}$ and $T\gg \mathrm{q}$, whose z-statistic (Eq. 23) possesses a Rayleigh distribution with variance $1/\sqrt{2}$. We zero all values of ${\widehat{{\varvec{\Theta}}}}_{{\varvec{\iota}}{\varvec{\iota}}}\left(f;j,j\right)$ with ${\varvec{Z}}\left(f;i,j\right)$ lower than a threshold to ensure a familywise error of type I. It should be noted that this debiasing and thresholding yields every iteration of the EM maximization in the **onestep hgLASSO** inverse solution (Eq. 18) or the second step in the multistep **hgLASSO** inverse solution (Eq. 13) by substituting ${\widehat{{\varvec{\Sigma}}}}_{{\varvec{\iota}}{\varvec{\iota}}}\left(f\right)$ in ${\widehat{{\varvec{\Psi}}}}_{{\varvec{\iota}}{\varvec{\iota}}}\left(f\right)$, a statistically guaranteed thresholded connectivity matrix.

38 $$\begin{array}{l}p\left({\varvec{Z}}\left(f;i,j\right)\right)=2{e}^{-{{\varvec{Z}}\left(f;i,j\right)}^{2}}{\varvec{Z}}\left(f;i,j\right)\\ {\varvec{Z}}\left(f;i,j\right)=\sqrt{\left\vert unb\left({\widehat{{\varvec{\Theta}}}}_{{\varvec{\iota}}{\varvec{\iota}}}\left(f;i,j\right)\right)\sqrt{T}/{\widehat{{\varvec{\Sigma}}}}_{{\varvec{\Theta}}}\left(f;i,j\right)\right\vert }\end{array}$$

#### Scalable and stable MAP1 inverse solution with the hgLASSO algorithm

The solution for the *Hermitian Graphical LASSO* (**hgLASSO**) that we present here is a transformation of its prior (Eq. 10) by means of the extension to the complex variable of the scaled Gaussian mixture procedure (Eq. 24)^121^; see Lemma 2 in SI V. Complex-valued Andrews and Mallows Lemma: Local Quadratic Approximation (LQA) of the Hermitian graphical LASSO (hgLASSO) prior (Lemma 2).

39 $$\begin{array}{l}p\left(\left.{{\varvec{\Theta}}}_{{\varvec{\iota}}{\varvec{\iota}}}\left(f\right)\right\vert {\varvec{\Gamma}}\left(f\right)\right)={\prod }_{i,j=1}^{\mathrm{q}}{N}_{1}^{T}\left(\left\vert {{\varvec{\Theta}}}_{{\varvec{\iota}}{\varvec{\iota}}}\left(f;i,j\right)\right\vert \vert 0,{{\varvec{\Gamma}}}^{2}\left(f;i,j\right)\right)\\ p\left({\varvec{\Gamma}}\left(f\right)\right)={\prod }_{i,j}^{\mathrm{q}}{Ga}^{T}\left({{\varvec{\Gamma}}}^{2}\left(f;i,j\right)\vert 1,{\alpha }_{{\varvec{\iota}}}^{2}{\mathbf{A}}_{{\varvec{\iota}}{\varvec{\iota}}}^{2}\left(i,j\right)/2\right)\end{array}$$

The scaled Gaussian mixture (Eq. 24) is a type of representation of the hgLASSO prior (Eq. 10) by a Gaussian distribution of ${{\varvec{\Theta}}}_{{\varvec{\iota}}{\varvec{\iota}}}\left(f\right)$ conditioned to other hyperparameters (variances ${\varvec{\Gamma}}\left(f\right)$) with a gamma distribution. As a result, we obtain a *Local Quadratic Approximation* (LQA) of the target function used in (Eqs. 13 or 18) $\mathcal{L}\left({\varvec{\Theta}}\left(f\right),{\varvec{\Gamma}}\left(f\right)\right)$ in terms of the weighted Hermitian graphical Ridge (hgRidge) due to the modified LASSO prior (Eq. 25). The estimation derived from the LQA poses a unique solution, given by Lemma 3 in SI VI. Concavity of the first-type maximum a posteriori with hgLASSO LQA prior (Lemma 3).

40 $$\begin{array}{l} {\left( {{{\widehat {\boldsymbol{\Theta }}}_{{\boldsymbol{\iota \iota }}}}\left( f \right),\widehat {\boldsymbol{\Gamma }}\left( f \right)} \right) = argmi{n_{{\boldsymbol{\Theta }}\left( f \right),{\boldsymbol{\Gamma }}\left( f \right)}}\left\{ {{\mathcal L}\left( {{{\boldsymbol{\Theta }}_{{\boldsymbol{\iota \iota }}}}\left( f \right),{\boldsymbol{\Gamma }}\left( f \right)} \right)} \right\}} \hfill \\ {{\mathcal L}\left( {{{\boldsymbol{\Theta }}_{{\boldsymbol{\iota \iota }}}}\left( f \right),{\boldsymbol{\Gamma }}\left( f \right)} \right) = - Tlog\left| {{{\boldsymbol{\Theta }}_{{\boldsymbol{\iota \iota }}}}\left( f \right)} \right| + Ttr\left( {{{\widehat {\boldsymbol{\Sigma }}}_{{\boldsymbol{\iota \iota }}}}\left( f \right){{\boldsymbol{\Theta }}_{{\boldsymbol{\iota \iota }}}}\left( f \right)} \right) + \frac{T}{2}{{\boldsymbol{\Theta }}_{{\boldsymbol{\iota \iota }}}}\left( f \right) \oslash {\boldsymbol{\Gamma }}\left( f \right)_2^2} \hfill \\ { + \frac{1}{2}\sum _{i,j = 1}^{\text{q}}log{{\boldsymbol{\Gamma }}^2}\left( {f;i,j} \right) + \frac{{T{\alpha ^2}}}{2}{\boldsymbol{\Gamma }}\left( f \right) \odot {{\boldsymbol{A}}_{{\boldsymbol{\iota \iota }}}}_2^2} \hfill \\ \end{array}$$

We can easily estimate the entries of the variance matrix ${\varvec{\Gamma}}\left(f\right)$ (Eq. 26) iteratively upon any estimated precision-matrix an inner cycle iteration (say the $l$-th) ${\widehat{{\varvec{\Theta}}}}_{{\varvec{\iota}}{\varvec{\iota}}}^{\left(l\right)}\left(\nu \right)$.

41 $$\begin{array}{l}{\widehat{{\varvec{\Gamma}}}}^{\left(l+1\right)}\left(f\right)={argmin}_{{\varvec{\Gamma}}\left(f\right)}\left\{\mathcal{L}\left({\widehat{{\varvec{\Theta}}}}_{{\varvec{\iota}}{\varvec{\iota}}}^{\left(l\right)}\left(f\right),{\varvec{\Gamma}}\left(f\right)\right)\right\}\\ {\widehat{{\varvec{\Gamma}}}}^{\left(l+1\right)}\left(f\right)={\left(\left(-{1}_{\rm{q}}+{\left({1}_{\rm{q}}+4{\alpha }_{{\varvec{\iota}}}^{2}{\mathbf{A}}_{{\varvec{\iota}}{\varvec{\iota}}}^{.2}\odot {\left\vert {\widehat{{\varvec{\Theta}}}}_{{\varvec{\iota}}{\varvec{\iota}}}^{\left(l\right)}\left(f\right)\right\vert }^{.2}\right)}^{.\frac{1}{2}}\right)\oslash \left(2{\alpha }_{{\varvec{\iota}}}{\mathbf{A}}_{{\varvec{\iota}}{\varvec{\iota}}}^{.2}\right)\right)}^{.\frac{1}{2}}\end{array}$$

Unfortunately, there is no explicit solution for ${\widehat{{\varvec{\Theta}}}}_{{\varvec{\iota}}{\varvec{\iota}}}^{\left(l\right)}\left(f\right)$ through (Eq. 25), something we solve by redefining it into standard precision-matrix ${\widetilde{{\varvec{\Theta}}}}_{{\varvec{\iota}}{\varvec{\iota}}}\left(\nu \right)$ (Eq. 27), which are weighted locally by the estimated variance matrix ${\varvec{\Gamma}}\left(f\right)$ from the previous iteration. This precision matrix also requires transforming the Wishart distribution associated with the GGS of (Eq. 9) and the HIGGS (Eq. 17) in terms of the standard sampled covariance matrix ${\widetilde{\widehat{{\varvec{\Sigma}}}}}_{{\varvec{\iota}}{\varvec{\iota}}}\left(f\right)$ due to Lemma 4 in SI VII. Standardization of the first-type likelihood with hgLASSO LQA prior (Lemma 4).

42 $$\begin{array}{l}{\widetilde{{\varvec{\Theta}}}}_{{\varvec{\iota}}{\varvec{\iota}}}\left(f\right)={{\varvec{\Theta}}}_{{\varvec{\iota}}{\varvec{\iota}}}\left(f\right)\oslash{\varvec{\Gamma}}\left(f\right)\\ {\widetilde{\widehat{{\varvec{\Sigma}}}}}_{{\varvec{\iota}}{\varvec{\iota}}}\left(f\right)={\left({\widehat{{\varvec{\Sigma}}}}_{{\varvec{\iota}}{\varvec{\iota}}}^{-1}\left(f\right)\oslash{\varvec{\Gamma}}\left(f\right)\right)}^{-1}\end{array}$$

This standardization poses the typical Hermitian graphical Ridge (hgRidge) problem^106^, without weights (Eq. 28).

43 $$\begin{array}{l} {p\left( {\left. {{{\widehat {\varvec{\Sigma }}}_{{\varvec{\iota \iota }}}}\left( f \right)} \right|{{\varvec{\Theta }}_{{\varvec{\iota \iota }}}}\left( f \right)} \right) = W_{\text{q}}^{\mathbb C}\left( {{{\widehat {\varvec{\Sigma }}}_{{\varvec{\iota \iota }}}}\left( f \right)|{T^{ - 1}}{\varvec{\Theta }}_{{\varvec{\iota \iota }}}^{ - 1}\left( \nu \right),T} \right)} \hfill \\ {{{\varvec{\Theta }}_{{\varvec{\iota \iota }}}}\left( f \right)\sim exp\left( {{{\varvec{\Theta }}_{{\varvec{\iota \iota }}}}\left( f \right)_2^2|T/2} \right)} \hfill \\ \end{array}$$

The solution to the hgRidge with standard empirical covariance ${\widetilde{\widehat{{\varvec{\Sigma}}}}}_{{\varvec{\iota}}{\varvec{\iota}}}\left(f\right)$ (Eq. 27) allows obtaining the standard precision-matrix ${\widehat{\widetilde{{\varvec{\Theta}}}}}_{{\varvec{\iota}}{\varvec{\iota}}}^{\left(l\right)}\left(f\right)$ (Eq. 28) and thus retrieving the actual precision-matrix ${{\varvec{\Theta}}}_{{\varvec{\iota}}{\varvec{\iota}}}\left(f\right)$ (Eq. 25). See Lemma 5 in SI VIII. Hermitian graphical Ridge (hgRidge) and hgLASSO LQA estimator (Lemma 5). This solution is in terms of the matrix square root operation (Eq. 29) that bounds the computational cost of this procedure, which requires a single cycle for the mutual estimation of the quantities ${{\varvec{\Theta}}}_{{\varvec{\iota}}{\varvec{\iota}}}\left(f\right)$ and ${\varvec{\Gamma}}\left(f\right)$.

44 $$\begin{array}{l}{\widehat{\widetilde{{\varvec{\Theta}}}}}_{{\varvec{\iota}}{\varvec{\iota}}}^{\left(l\right)}\left(f\right)=-\frac{1}{2}{\widetilde{\widehat{{\varvec{\Sigma}}}}}_{{\varvec{\iota}}{\varvec{\iota}}}^{\left(l\right)}\left(f\right)+\frac{1}{2}\sqrt{{\left({\widetilde{\widehat{{\varvec{\Sigma}}}}}_{{\varvec{\iota}}{\varvec{\iota}}}^{\left(l\right)}\left(f\right)\right)}^{2}+4{\mathbf{I}}_{\rm{q}}}\\ {\widetilde{\widehat{{\varvec{\Sigma}}}}}_{{\varvec{\iota}}{\varvec{\iota}}}^{\left(l\right)}\left(f\right)={\left({\widehat{{\varvec{\Sigma}}}}_{{\varvec{\iota}}{\varvec{\iota}}}^{-1}\left(f\right)\oslash {\widehat{{\varvec{\Gamma}}}}^{\left(l\right)}\left(f\right)\right)}^{-1}\\ {\widehat{{\varvec{\Theta}}}}_{{\varvec{\iota}}{\varvec{\iota}}}^{\left(l\right)}\left(f\right)={\widehat{\widetilde{{\varvec{\Theta}}}}}_{{\varvec{\iota}}{\varvec{\iota}}}^{\left(l\right)}\left(f\right) \odot {\widehat{{\varvec{\Gamma}}}}^{\left(l\right)}\left(f\right)\end{array}$$

### Validating performance for HIGGS inverse solutions

#### Simulations

**Figure 4:**
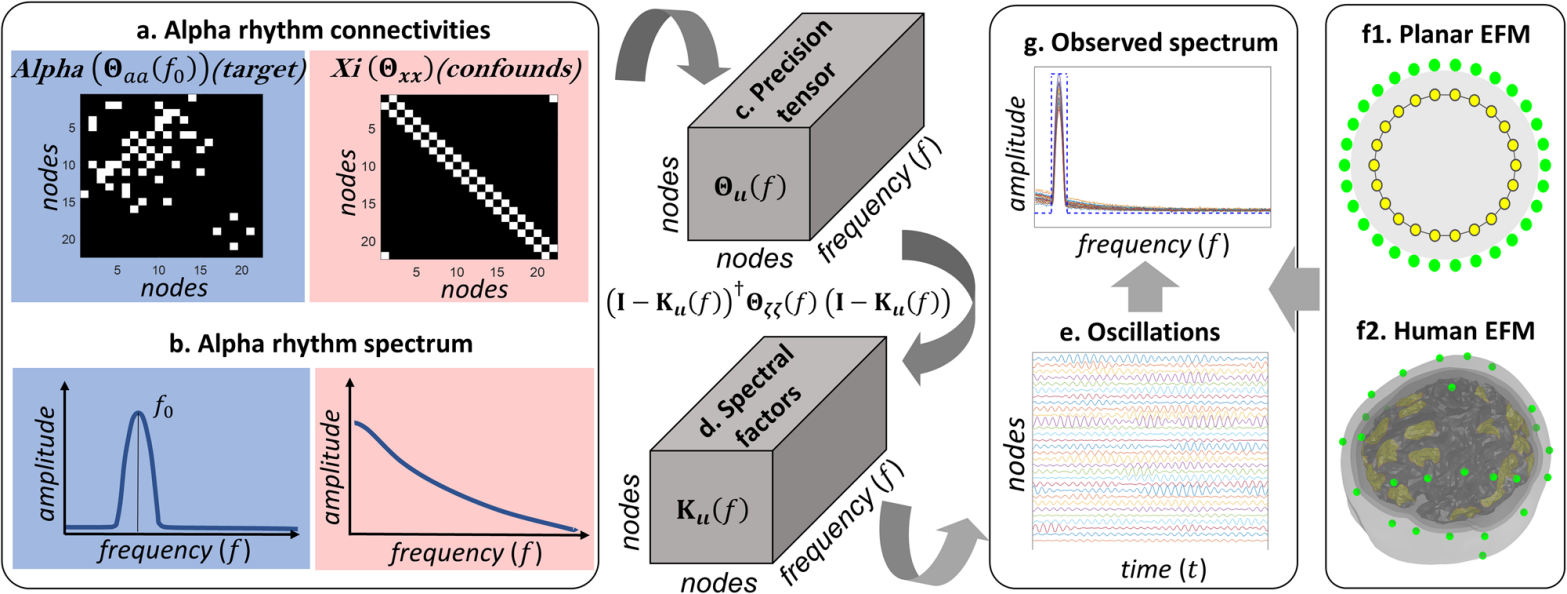
Specification of a linear autoregressive dynamics in the frequency domain based on the Xi and Alpha model (**a**). Alpha connectivity is the ground true to be mixed later with that of the Xi process. The starting point are these Alpha and Xi precision-matrices at 10 Hz for an oscillatory network process represented in a binary map that corresponds to edges in such network graph-elements. (**b**) This is extended for all frequencies via an amplitude-phase transformation to recreate (**c**). The precision-tensor employing (**d**) directed transfer function tensor for the Alpha process by means of its composition in spectral factors with the Xi innovations The factors are derived from the central slice (10 Hz) by means of the eigen-decomposition. The process cross-spectrum tensor is obtained by slice-inverting the precision tensor producing e. brain-oscillations by means of a Hermitian random Gaussian generator. (**f**) Two types of forward models, (**f1**) planar and (**f2**) human project these oscillations to the sensor space producing g. the Xi Alpha observations.

We created simulations (Fig. 4) based on second-order stochastic stationary dynamics (Eq. 9) following from an underlying *Gaussian Graphical Spectral* (GGS) model. This Hermitian precision matrix (Fig. 4a) defining this GGS produces brain oscillations that resemble the so-called EEG Xi-Alpha model in the spectrum (Fig. 4b)^122^. In other words, this autoregressive dynamic ${\varvec{\iota}}\left(t\right)$ is defined from ${\varvec{\iota}}\left(t,f\right)$ (Eq. 11), produced by a Hermitian precision tensor (connectivity) ${{\varvec{\Theta}}}_{{\varvec{\iota}}{\varvec{\iota}}}\left(f\right)$ at all frequencies which also causes the oscillations in the alpha peak (Fig. 4c). This Hermitian precision tensor originates from spectral factors ${\mathbf{\rm K}}_{{\varvec{\iota}}{\varvec{\iota}}}\left(f\right)$ (Fig. 4d) or the Hilbert transform of multiply lagged connectivity ${\mathbf{\rm K}}_{{\varvec{\iota}}{\varvec{\iota}}}\left(\tau \right)$ producing broad band dynamics (Fig. 4e).

This connectivity ${{\varvec{\Theta}}}_{{\varvec{\iota}}{\varvec{\iota}}}\left(f\right)$ (Fig. 4a) is employed as a baseline to compare the results and is more in correspondence with the actual target for state-of-the-art methods. The simulation pipeline that was repeated 100 times to produce the same amount of possible ground truth and observations is as follows.

1. Definition of random Hermitian precision-matrix ${{\varvec{\Theta}}}_{aa}\left({f}_{0}\right)$ (Fig. 4a) (ground truth functional connectivity) given for a referential frequency component ${f}_{0}$ meant to be the center of the alpha peak (Fig. 4b) (${f}_{0}=10\mathrm{ Hz}$) in the cortical network subspace. The elements of this matrix must lie in the unitary circle of the complex plane for the reliability of binary classification measures used in this validation.

2. Construction of a synthetic spectral factorization for ${{\varvec{\Theta}}}_{aa}\left({f}_{0}\right)$ (Fig. 4c) or spectral directed model used to design a precision tensor at all frequencies ($f\in {\mathbb{F}}$). This factorization was performed assuming that the reference spectral factors at (${f}_{0}=10\mathrm{Hz}$) are derived from a Hermitian eigen decomposition ${{\varvec{\Theta}}}_{aa}\left({f}_{0}\right)=\mathbf{U}\mathbf{D}{\mathbf{U}}^{\dagger}$ (Eq. 45), where ${\mathbf{\rm K}}_{{\varvec{\iota}}{\varvec{\iota}}}\left({f}_{0}\right)$ is the directed transfer function at ${f}_{0}$ extracted from the $\mathbf{U}\mathbf{D}{\mathbf{U}}^{\dagger}$ elements.

45 $$\begin{array}{l}{{\varvec{\Theta}}}_{aa}\left({f}_{0}\right)={\left(\mathbf{I}-{\mathbf{\rm K}}_{{\varvec{\iota}}{\varvec{\iota}}}\left({f}_{0}\right)\right)}^{\dagger}\left(\mathbf{I}-{\mathbf{\rm K}}_{{\varvec{\iota}}{\varvec{\iota}}}\left({f}_{0}\right)\right)\\ {\mathbf{\rm K}}_{{\varvec{\iota}}{\varvec{\iota}}}\left({f}_{0}\right)=\mathbf{I}-{\mathbf{U}}^{\dagger}\sqrt{\mathbf{D}}\end{array}$$

3. Considering discrete-time lags in ${\mathbf{\rm K}}_{{\varvec{\iota}}{\varvec{\iota}}}\left(\tau \right)$ as in equation (Eq. 9) encoded in a matrix ${\mathbf{\rm T}}_{aa}$, where ${\mathbf{\rm T}}_{aa}\left(i,j\right)$ is the lag for directed interactions directed $\left\{\dots ,{\varvec{\iota}}\left(t;i\right)\leftarrow{\varvec{\iota}}\left(t;j\right),\dots \right\}$, we represent ${\mathbf{\rm K}}_{{\varvec{\iota}}{\varvec{\iota}}}\left(\tau \right)$ (Eq. 46) as proportional to a constant connectivity matrix ${\mathbf{\rm K}}_{aa}$ and the Dirac delta matrix function of the lags ${\varvec{\Delta}}\left(\tau -{\mathbf{\rm T}}_{aa}\right)$.

46 $$\begin{array}{l}{\mathbf{\rm K}}_{{\varvec{\iota}}{\varvec{\iota}}}\left(\tau \right)\propto {\mathbf{\rm K}}_{aa}{\varvec{\Delta}}\left(\tau -{\mathbf{\rm T}}_{aa}\right)\\ {\mathbf{\rm T}}_{aa}=phase\left({\mathbf{\rm K}}_{{\varvec{\iota}}{\varvec{\iota}}}\left({f}_{0}\right)\right)/{f}_{0}\end{array}$$

These lags are associated with the spectral ${\mathbf{\rm K}}_{{\varvec{\iota}}{\varvec{\iota}}}\left({f}_{0}\right)$ at ${f}_{0}$ given the phase relation (Eq. 47), and the directed transfer function tensor ${\mathbf{\rm K}}_{{\varvec{\iota}}{\varvec{\iota}}}\left(f\right)$ at all frequencies ($f\in {\mathbb{F}}$) (Fig. 4d) as in (Eq. 10) is then obtained from the definition for ${\mathbf{\rm K}}_{{\varvec{\iota}}{\varvec{\iota}}}\left(\tau \right)$ (Eq. 46) modulated by a Gaussian spectral form with center in the alpha peak (${f}_{0}=10\mathrm{Hz}$).

47 $$\begin{array}{l}{\mathbf{\rm K}}_{{\varvec{\iota}}{\varvec{\iota}}}\left({f}_{0}\right)={\mathbf{\rm K}}_{aa}exp\left(\mathbf{j}{\mathbf{\rm T}}_{aa}{f}_{0}\right)\\ {\mathbf{\rm K}}_{{\varvec{\iota}}{\varvec{\iota}}}\left(f\right)={\mathbf{\rm K}}_{aa}exp\left(\mathbf{j}{\mathbf{\rm T}}_{aa}f\right)exp\left(\left.{\left(f-{f}_{0}\right)}^{2}\right\vert {\theta }_{aa}\right)\end{array}$$

4. The precision tensor ${{\varvec{\Theta}}}_{{\varvec{\iota}}{\varvec{\iota}}}\left(f\right)$ (Fig. 4c) is then recomposed from the spectral factors (Eq. 48) assuming directed alpha transfer function tensor ${\mathbf{\rm K}}_{{\varvec{\iota}}{\varvec{\iota}}}\left(f\right)$ (Eq. 47) and Xi innovations precision tensor ${{\varvec{\Theta}}}_{{\varvec{\zeta}}{\varvec{\zeta}}}\left(f\right)$ as in (Eq. 11). The Xi factor was construed by following steps similar to alpha but modulated by a right-sided Gaussian spectral form with central frequency (${f}_{0}=0\mathrm{Hz}$). The precision matrix ${{\varvec{\Theta}}}_{xx}$ was the inverse of the surface Laplacian in the whole cortical space, and the lags for the precision tensor construction were set to zero, corresponding to the properties of this process.

48 $$\begin{array}{l}{{\varvec{\Theta}}}_{{\varvec{\iota}}{\varvec{\iota}}}\left(f\right)={\left(\mathbf{I}-{\mathbf{\rm K}}_{{\varvec{\iota}}{\varvec{\iota}}}\left(f\right)\right)}^{\dagger}{{\varvec{\Theta}}}_{{\varvec{\zeta}}{\varvec{\zeta}}}\left(f\right)\left(\mathbf{I}-{\mathbf{\rm K}}_{{\varvec{\iota}}{\varvec{\iota}}}\left(f\right)\right)\\ {{\varvec{\Theta}}}_{{\varvec{\zeta}}{\varvec{\zeta}}}\left(f\right)={{\varvec{\Theta}}}_{xx}exp\left(\left.{f}^{2}\right\vert {\theta }_{xx}\right)\end{array}$$

5. The precision tensor slicewise inverted ${{\varvec{\Sigma}}}_{{\varvec{\iota}}{\varvec{\iota}}}\left(f\right)={{\varvec{\Theta}}}_{{\varvec{\iota}}{\varvec{\iota}}}^{-1}\left(f\right)$ (Eq. 48) (this is the ground truth covariance or cross-spectrum ${{\varvec{\Sigma}}}_{{\varvec{\iota}}{\varvec{\iota}}}\left(f\right)$) is used to obtain complex-valued vectors of the Fourier transform (Eq. 49). Equivalent in this case to the Hilbert transform ${\varvec{\iota}}\left(t,f\right)$ ($t\in {\mathbb{T}}$) by means of a Hermitian Gaussian random generator with sample number $T=600$ at every frequency ($f\in {\mathbb{F}}$). Brain oscillation time series ${\varvec{\iota}}\left(t\right)$ (Fig. 4e) are obtained by means of the inverse Fourier transform (${\mathcal{F}}^{-1}$) across frequencies ($f\in {\mathbb{F}}$) of these samples ${\varvec{\iota}}\left(t,f\right)$ to obtain the same number of time instances ($t\in {\mathbb{T}}$).

49 $$\begin{array}{l}p\left({\varvec{\iota}}\left(t,f\right)\left\vert {{\varvec{\Theta}}}_{{\varvec{\iota}}{\varvec{\iota}}}\left(f\right)\right.\right)={N}^{\mathbb{C}}\left({\varvec{\iota}}\left(t,f\right)\vert 0,{{\varvec{\Theta}}}_{{\varvec{\iota}}{\varvec{\iota}}}^{-1}\left(f\right)\right)\\{\varvec{\iota}}\left(t\right)={\mathcal{F}}^{-1}\left({\varvec{\iota}}\left(t,f\right)\right)\end{array}$$

6. The Xi-Alpha process ${\varvec{\iota}}\left(t,f\right)$ in the cortical network subspace (Eq. 49) was projected to obtain observations (Fig. 4g) at the sensor space ${\varvec{v}}\left(t\right)$ (Eq 50), time-domain equivalent of (Eq. 16) with corresponding forward-operator ${\mathbf{L}}_{{\varvec{v}}{\varvec{\iota}}}$ (Fig. 4f), adding the Xi process and a white noise process in the whole cortical space. A white noise process ${\varvec{\xi}}\left(t\right)$ in the sensor space ${\mathbb{E}}$ was also added to the projected source space process. The composition of the confounding processes (Xi process defined by ${{\varvec{\Theta}}}_{xx}$ and source and white sensor noises) with the alpha process defined by ${{\varvec{\Theta}}}_{aa}\left({f}_{0}\right)$ was adjusted to keep spectral energy in the alpha band (8–12 Hz) of 10% of the alpha process energy.

50 $${\varvec{v}}\left(t\right)={\mathbf{L}}_{{\varvec{v}}{\varvec{\iota}}}{\varvec{\iota}}\left(t\right)+{\varvec{\xi}}\left(t\right)$$

Two different simulations based on the *Electromagnetic Forward Model* (EFM) with EFM forward-operator ${\mathbf{L}}_{{\varvec{v}}{\varvec{\iota}}}$ (Eq. 50) were defined: For an idealized “planar EFM” head (Fig. 4f1) and realistic “human EFM” head (Fig. 4f2) to produce the Xi-Alpha observations (Fig. 4g). The planar EFM (Fig. 4f1) was computed based on the bidimensional geometry of two concentric circles defining a planar cortex and scalp and a layout of equidistant planar scalp sensors (30 sensors). The human EFM (Fig. 4f2) was computed for the cortical surface extracted from a healthy subject T1 image, with the *Boundary Element Method* (BEM) implemented in SPM and a layout of the extended 10–20 system (30 sensors). A cortical network was defined on a subspace of 22 points for both the planar and human EFMs. For the planar cortex, these were equidistantly located, and for the human cortex, they were randomly located across different areas of the left (L) and right (R) hemispheres: Occipital (LO and RO), Temporal (LT and RT), Parietal (LP and RP) and Frontal (LF and RF).

#### Experimental confirmation

**Figure 5:**
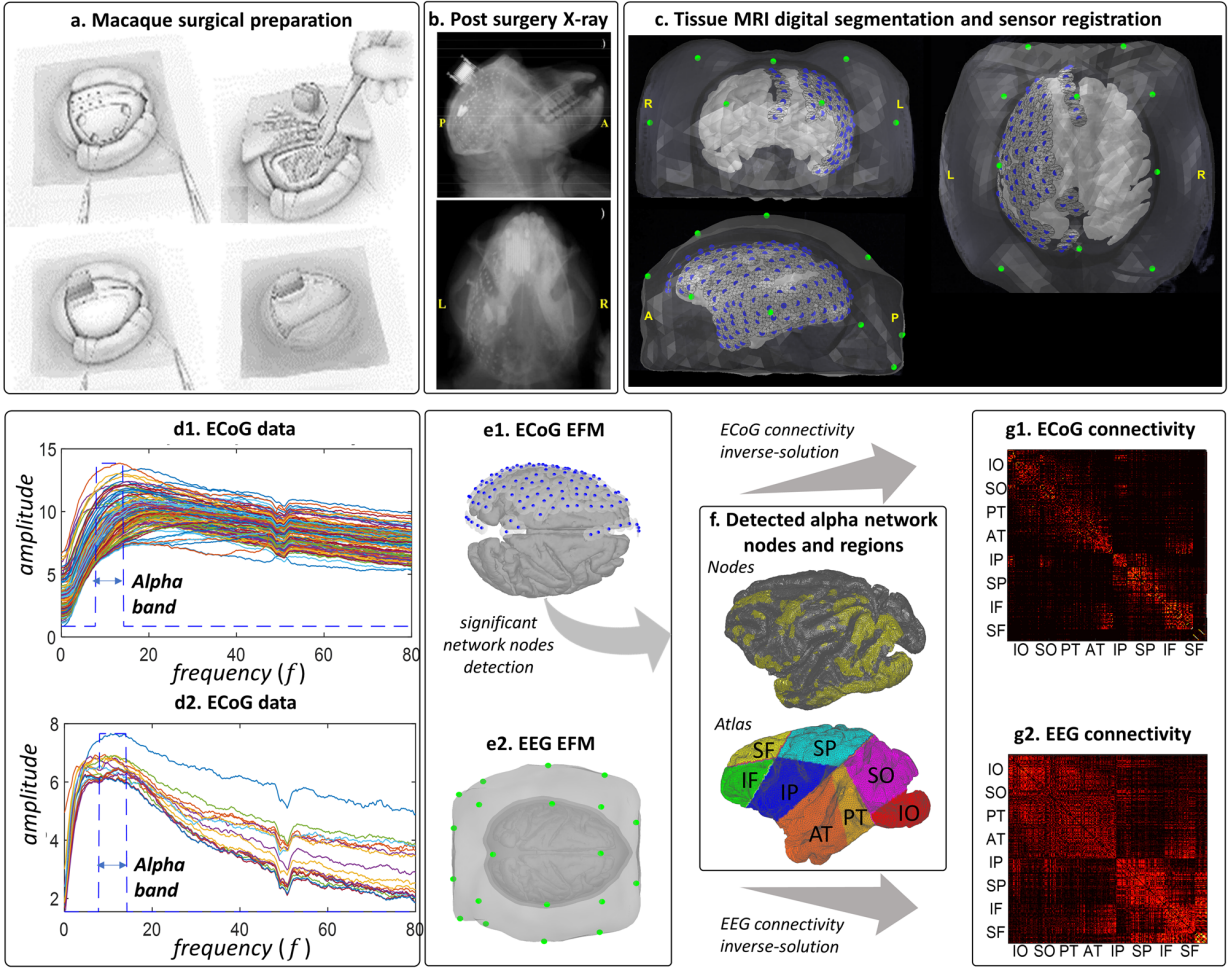
Confirmation of HIGGS connectivity based in EEG that is recorded simultaneously with ECoG implanted in the macaque. (**a**) Surgical preparation for implantation onto the macaque cortex macaque of a high-density ECoG array embedded in a silicon layer. (**b**) A post-surgical X-ray image showing this implantation. (**c**) Digital preparation based on the macaque MRI segmentation of the cortex, inner skull, outer skull and scalp, and conductance model for this segmentation based on registration with the MRI of the high-density ECoG implantation (128 blue sensors) and low-density EEG layout (20 green sensors). (**d**) Power spectral density of the simultaneous recordings for ECoG (**d1**) and EEG (**d2**) highlighting the alpha band within 8–14 Hz employed later as data for the identification of network connectivity. (**e**) Electromagnetic Forward Model (EFM) employed to compute the connectivity inverse-solutions from the ECoG (**e1**), with lead field dependent on two conductance layers (cortex and silicon), and EEG (**e2**), with Lead Field dependent on four conductance layers (cortex, inner skull, outer skull and scalp). ECoG recordings and their Lead Fields provide a more fine-grained reference for confirming connectivity estimators and measures of distortions for the EEG (**f**). Significant cortical subspace based on the ECoG alpha band data to facilitate the comparison in terms of connectivity due to low-density of the EEG. The detected subspace is revealing a large-scale alpha cortical network that extended over the inferior occipital (IO), superior occipital (SO), posterior temporal (PT), anterior temporal (AT), inferior parietal (IP), superior parietal (SP), inferior frontal (IF) and superior frontal (SF) areas. (**g**) For the subspace covered by this network the HIGGS connectivity inverse-solutions were computed from the ECoG (**g1**) and EEG (**g2**).

For the confirmation of HIGGS connectivity, we compared EEG against a more direct technique: electrocorticography (ECoG) (Fig. 5) leveraging the unique experimental setup that offers the advantage of large brain coverage ECoG recordings on a healthy macaque^123^. This comparison scenario is particularly interpretable in terms of the effect of volume conduction heterogeneities since the EEG is hidden under several tissue layers (Fig. 5e2), and the ECoG is instead hidden under only one (Fig.  5e1). See the macaque preparation for the surgical implantation of this ECoG in (Fig. 5a) that, which is shown in the postsurgical X-ray (Fig. 5b). The macaque sensor layouts for the simultaneous ECoG/EEG recordings consisted of 128 ECoG sensors placed upon the cortical surface in the left hemisphere and a low-density array of 20 EEG scalp sensors. The relative distribution of the macaque cortical surface segmented from the MRI is shown in (Fig. 5c).

The raw EEG/ECoG data is freely available as part of the Neurotycho project http://www.www.neurotycho.org/. The EEG ${{\varvec{v}}}^{EEG}\left(t\right)$ and ECoG ${{\varvec{v}}}^{ECoG}\left(t\right)$ were recorded simultaneously in the resting state condition. During the experimental session, the monkey was awake, blindfolded, and constrained to a sitting position. Henceforth, our study only refers to the processing of this data and not experimental manipulation, ethics are deferred to the previous publications about the same data.

Both EEG and ECoG were synchronized to the trigger signal and downsampled to 1000 Hz, keeping 2 min of recordings. The artifact removal procedure included linear detrending with the L1TF package, average DC subtraction, and 50 Hz notch filtering. The spectral analysis (Fig. 5d) of both ECoG (Fig. 5d1) and EEG (Fig. 5d2) signals reveals a larger power spectral density within the band (8–14 Hz) associated with the macaque alpha oscillations. The forward operators were obtained from a head conductivity model (Fig. 5e) for the ECoG ${\mathbf{L}}_{{\varvec{v}}{\varvec{\iota}}}^{ECoG}$ (Fig. 5e1) and EEG ${\mathbf{L}}_{{\varvec{v}}{\varvec{\iota}}}^{EEG}$ (Fig. 5e2) through FEM computations in SimBio using the macaque individual T1 MRI segmentation. The head model included five conductivity compartments: cortex, ECoG silicon layer, inner skull, outer skull, and scalp.

We work at a subspace of cortical sources detected from ECoG, and in a similar fashion to the simulation study (Fig. 5f), extracting the connectivity (Fig. 5g) with all methods from both ECoG (Fig. 5g1) and EEG (Fig. 5g2) observations. We screen out the significant cortical network nodes (Fig. 5f) from the ECoG by means of implementation for spectral analysis of the *Structured Sparse Bayesian Learning* (SSBL)^69^. This implementation uses assumptions similar to those of HIGGS but with a diagonal covariance structure. We offer technical details in^124^.

The detection (Fig. 5f) corresponding to the alpha band oscillations (Fig. 5d1) yielded a large-scale network of distributed nodes, which strongly correlates with the findings for these ECoG cortical patterns in the left hemisphere^125^. The more extended Occipital (O) oscillations were accompanied by secondary cortical oscillations extended over the Temporal (T), Parietal (P), and Frontal (F) areas. This large-scale analysis differs from previously reported studies, which were limited to P <—> F interactions at ECoG sensor space as in^126^. We consider eight regions of interest (ROI) extracted manually from the left hemisphere cortical segmentation. These are Inferior Occipital (IO), Superior Occipital (SO), Posterior Temporal (PT), Anterior Temporal (AT), Inferior Parietal (IP), Superior Parietal (SP), Inferior Frontal (IF), and Superior Frontal (SF).

## Results

### Unbiased functional connectivity via hgLASSO inverse solution of the GGS model

**Figure 6:**
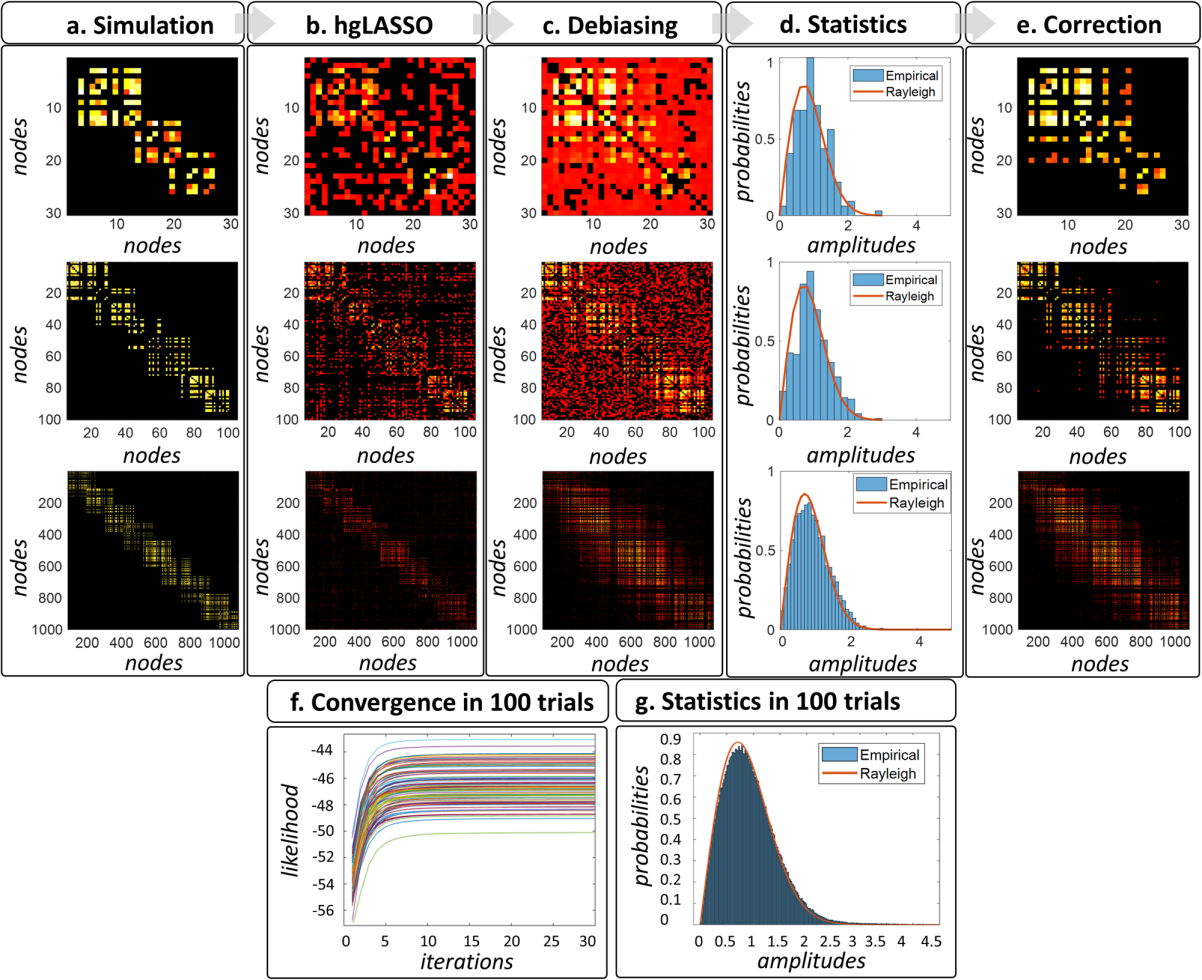
Experiment to evaluate unbiasedness conditions of the Hermitian Graphical LASSO (hgLASSO) algorithm in different scales at the top: low (first row), high (second row) and ultrahigh (third row). The hgLASSO algorithm performs the MAP1 for the presicion-matrix as described in Materials and methods sections "Gaussian graphical spectral (GGS) model and MAP1 inverse solution with Hermitian graphical LASSO (hgLASSO)" and "GGS model MAP1 inverse solution via Hermitian graphical LASSO (hgLASSO) algorithm". From left to right in hot colormap (**a**) the typical simulated precision-matrix, (**b**) the hgLASSO estimation, (**c**) the debiased estimator, with (**d**) the empirical distribution, compared of the theoretical Rayleigh distribution, and (**e**) the corrections due to thresholds of this distribution. At the bottom for the ultra-high dimensionality the robust convergence pattern in 30 iterations of the hgLASSO likelihood in 100 trials (**f**) and the seemingly perfect Rayleigh statistics in these 100 trials (**g**).

As an essential step (Fig. 6), we verify the properties of the MAP1 estimator for the precision matrix ${{\varvec{\Theta}}}_{{\varvec{\iota}}{\varvec{\iota}}}\left(f\right)$ representing functional connectivity as described by a Gaussian graphical spectral (GGS) model of oscillatory brain networks at a generic frequency $f$. See Materials and Methods section "Gaussian graphical spectral (GGS) model and MAP1 inverse solution with Hermitian graphical LASSO (hgLASSO)" for the interpretation of such a functional network model and MAP1 inverse solution ${\widehat{{\varvec{\Theta}}}}_{{\varvec{\iota}}{\varvec{\iota}}}\left(f\right)$ for the functional connectivity. We remind the reader that the algorithm revindicated for this MAP1 inverse solution places the *Hermitian graphical LASSO* (**hgLASSO**) a priori model upon the Hermitian graph elements in ${{\varvec{\Theta}}}_{{\varvec{\iota}}{\varvec{\iota}}}\left(f\right)$, which explains brain-oscillations ${\varvec{\iota}}\left(t,f\right)$ in the GGS model. In Materials and Methods section "GGS model MAP1 inverse solution via Hermitian graphical LASSO (hgLASSO) algorithm", we describe the implementation of the **hgLASSO** algorithm that we demonstrate here for the first time fulfilling stable and scalable unbiasedness conditions. Here, amplitudes $\left\vert {{\varvec{\Theta}}}_{{\varvec{\iota}}{\varvec{\iota}}}\left(f\right)\right\vert$ of the Hermitian graph elements are employed as the proxy for functional connectivity and to illustrate the algorithmic properties. The **hgLASSO** a priori model is incorporated into both multistep MAP1 and **onestep** MAP2 inverse solutions, as described in Materials and Methods section "Hidden Gaussian graphical spectral (HIGGS) model and inverse solutions via multistep MAP1 and onestep MAP2" for the *Hidden GGS* (HIGGS) model explaining oscillations ${\varvec{v}}\left(t,f\right)$.

We illustrate the properties of our algorithm with an example at different dimensionalities, 30, 100, and 1000, yielding 900, 10,000, and 1000,000 functional connectivities to be estimated, respectively. Our validation strategy (Fig. 6) is to compare the empirical distribution of the empirical distribution of the unbiased statistic for the estimated precision matrix ${\widehat{{\varvec{\Theta}}}}_{{\varvec{\iota}}{\varvec{\iota}}}\left(f\right)$ against the Rayleigh distribution for the theoretical ${{\varvec{\Theta}}}_{{\varvec{\iota}}{\varvec{\iota}}}\left(f\right)$. A Rayleigh distribution is derived from the complex-valued extension of^82^ for the GGS model described in Materials and Methods section "GGS model MAP1 inverse solution via Hermitian graphical LASSO (hgLASSO) algorithm".

First, we employ a blockwise chained (blocks were mutually dependent) simulated precision-matrix structure ${{\varvec{\Theta}}}_{{\varvec{\iota}}{\varvec{\iota}}}\left(f\right)$ as in^81^, illustrated here with hot colormaps of a typical trial (Fig. 6a). These matrices were integrated into a Hermitian Gaussian generator generating the sampled covariance matrix from sampled instances (a sample number of 100 times the matrix dimensions). Second, ${\widehat{{\varvec{\Theta}}}}_{{\varvec{\iota}}{\varvec{\iota}}}\left(f\right)$, a precision matrix, is estimated with our **hgLASSO** algorithm (Fig. 6b), and third, from this estimate, the unbiased precision matrix $unb\left({\widehat{{\varvec{\Theta}}}}_{{\varvec{\iota}}{\varvec{\iota}}}\left(f\right)\right)$ (Fig. 6c) is computed as proposed by the theory. Fourth, we obtain the histogram of a z-statistic ${\varvec{Z}}\left(f\right)$ (Fig. 6d) for this unbiased estimate at the null hypothesis matrix entries (zero precision-matrix entries ${{\varvec{\Theta}}}_{{\varvec{\iota}}{\varvec{\iota}}}\left(f;i,j\right)=0$), and this histogram accurately resembles the theoretical Rayleigh distribution. Computing the z-statistic was performed by scaling the unbiased precision matrix using the theoretical variances ${\widehat{{\varvec{\Sigma}}}}_{{\varvec{\Theta}}}\left(f;i,j\right)$.

Fifth, we remove the values under the Rayleigh threshold (Rayleigh corrected precision-matrix) obtained from the 0.05 p value of the theoretical Rayleigh distribution. With the Rayleigh threshold, from the originally dense unbiased precision matrix (Fig. 6c), we obtain an improved corrected result (Fig. 6e) in comparison to the sparse biased hgLASSO precision matrix (Fig. 6b). The **hgLASSO** (base of all estimated maps), as we illustrate in the ultrahigh dimensionality, a robust convergence pattern (Fig. 6f) for multiple repetitions of the experiment (100 trials). The z-statistic histograms for these 100 trials reflect the high coincidence with the theoretical Rayleigh distribution (Fig. 6g).

### Erroneous multistep and exact onestep functional connectivity for the HIGGS model in simulations

**Figure 7:**
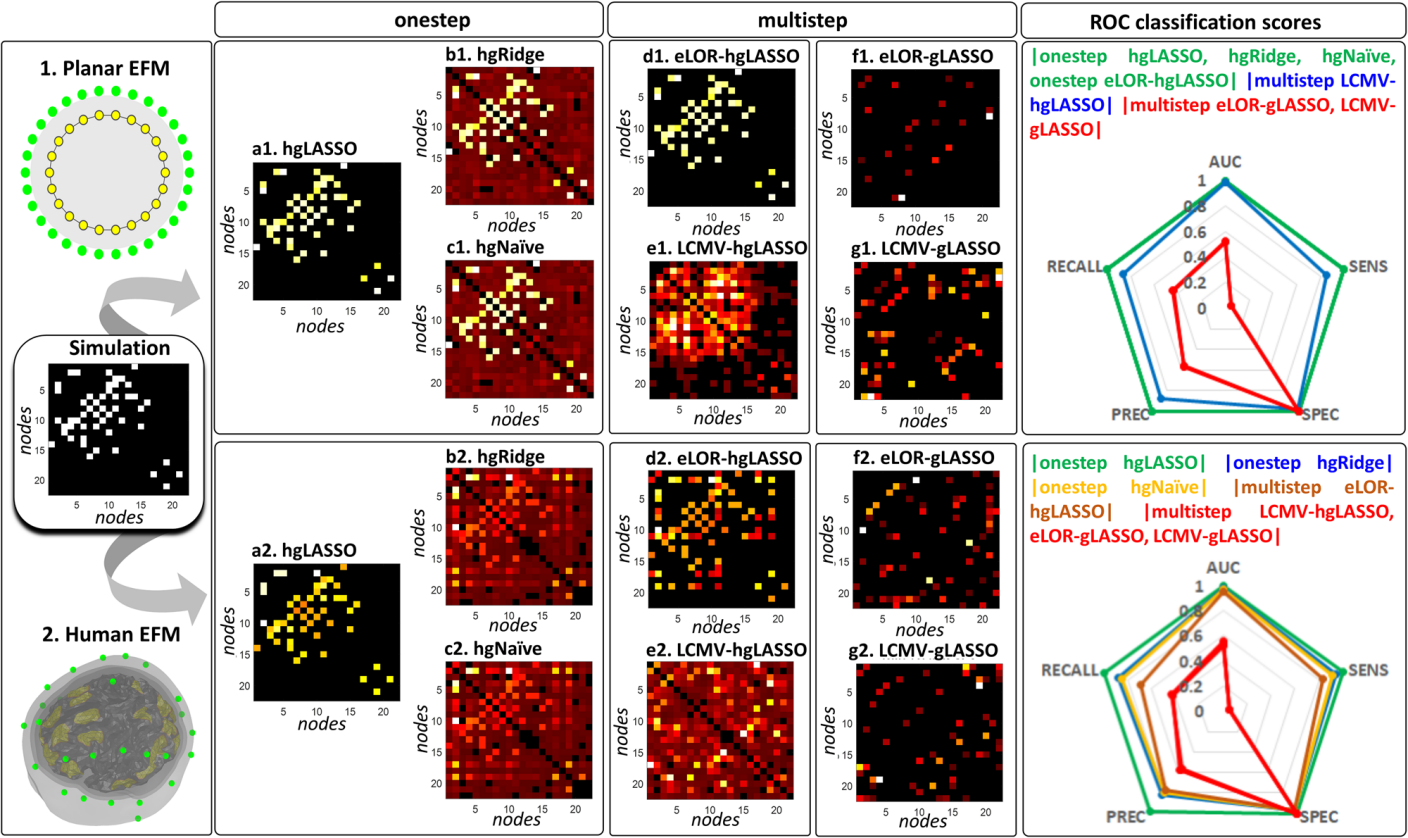
Simulated experiment of a relatively simple oscillatory network HIGGS model (left-center) producing observations through (1) an idealized “planar” (left-top) or (2) average “human” (left-bottom) EFM. Estimation errors regarding a ground-true Hermitian precision-matrix with binary amplitudes (left-center) may be judged in the colormaps of the estimation under the planar (**a1–g1**) and human (**a2–g2**) EFM. These estimation are characteristic of multistep methods (based on first-step ELORETA and LCMV) in an average EEG situation (**d2,e2**) even employing the hgLASSO unbiased estimator. With same multistep methods the estimation errors of the state-of-the-art gLASSO approximating the Hermitian graph-elements in this HIGGS (**f1,g1;f2,g2**) fall beyond even in ideal circumstances. In 100 trials of a binary connectivity ROC measures show these estimation errors and improvement with our direct solution with hgLASSO (**a1,a2**) which may not be possible with hgRidge or hgNaïve estimation (**b1,c1,b2,c2**).

The proof of concept for the HIGGS model (Fig. 7) employs random precision-matrices instances ${{\varvec{\Theta}}}_{{\varvec{\iota}}{\varvec{\iota}}}\left(f\right)$ similar to the previous results in section "Unbiased functional connectivity via hgLASSO inverse solution of the GGS model" but yielding binary functional connectivity as defined with the amplitude of Hermitian graph-elements $\left\vert {{\varvec{\Theta}}}_{{\varvec{\iota}}{\varvec{\iota}}}\left(f\right)\right\vert$. Our binary “ground-truth” functional connectivity $\left\vert {{\varvec{\Theta}}}_{{\varvec{\iota}}{\varvec{\iota}}}\left(f\right)\right\vert$ (Fig. 7 left-center) is a much more plausible target of estimation errors via classification scores (Fig. 7 right).

Simulations of the HIGGS model producing EEG observations ${\varvec{v}}\left(t,f\right)$ are due to brain oscillations ${\varvec{\iota}}\left(t,f\right)$ steaming from an alpha rhythm peaking at 10 Herts, which follows from an interpretation of the GGS model in terms of second-order stochastic stationary dynamics. See the description of these simulations in Materials and Methods section "Validating performance for HIGGS inverse solutions". The brain oscillations ${\varvec{\iota}}\left(t,f\right)$ are projected through forward operators ${\mathbf{L}}_{{\varvec{v}}{\varvec{\iota}}}$ that follow two types of EFM geometrical designs: (1) Planar EFM (Fig. 7 left-top), recreating an ideal circumstance where concentric circles recreate the cortical network, head and EEG sensor geometries, and EEG fields lead through homogeneous conductance. (2) Human EFM (Fig. 7 left-bottom), recreating an average human EEG circumstance. Then, the GGS model precision matrix ${{\varvec{\Theta}}}_{{\varvec{\iota}}{\varvec{\iota}}}\left(f\right)$ (Fig. 7 left-center), which describes brain oscillations ${\varvec{\iota}}\left(t,f\right)$ at the alpha peak, is the target of the estimation ${\widehat{{\varvec{\Theta}}}}_{{\varvec{\iota}}{\varvec{\iota}}}\left(f\right)$ with all types of inverse solutions exposed here (Fig. 7 a1-g1, a2-g2). See these inverse solutions in Materials and Methods section "Hidden Gaussian Graphical Spectral (HIGGS) model and inverse solutions via multistep MAP1 and onestep MAP2".

First, three **onestep** MAP2 inverse solutions are implemented as successive approximations due to MAP1 inverse solutions within an Expectation–Maximization (EM) loop for 1) brain oscillations $\widehat{{\varvec{\iota}}}\left(f,t\right)$ in the EM expectation stage and 2) functional connectivity ${\widehat{{\varvec{\Theta}}}}_{{\varvec{\iota}}{\varvec{\iota}}}\left(f\right)$ in the EM maximization stage. The functional connectivity MAP1 (maximization stage) determines a GGS model precision matrix ${\widehat{{\varvec{\Theta}}}}_{{\varvec{\iota}}{\varvec{\iota}}}\left(f\right)$ from the expected covariance matrix ${\widehat{{\varvec{\Psi}}}}_{{\varvec{\iota}}{\varvec{\iota}}}\left(f\right)$ via the unbiased *Hermitian Graphical LASSO* (**hgLASSO**) algorithm (Fig. 7a1, a2). See this algorithm in Materials and Methods sections "Gaussian Graphical Spectral (GGS) model and MAP1 inverse solution with Hermitian Graphical LASSO (hgLASSO)" and "GGS model MAP1 inverse solution via Hermitian Graphical LASSO (hgLASSO) algorithm". Here, we also introduce violations to the **hgLASSO** unbiasedness conditions in the functional connectivity MAP1 via the *Hermitian graphical Ridge* (**hgRidge**) (Fig. 7b1, b2) and *Hermitian graphical Naïve* (**hgNaïve**) (Fig. 7c1, c2).

Second, the **multistep** MAP1 inverse solutions are implemented for 1) brain-oscillations $\widehat{{\varvec{\iota}}}\left(f,t\right)$ via **ELORETA** (Fig. 7d1, d2)^56^ and **LCMV** (Fig. 7e1, e2)^55^ in the first step and 2) functional connectivity ${\widehat{{\varvec{\Theta}}}}_{{\varvec{\iota}}{\varvec{\iota}}}\left(f\right)$ in the second step and from the sampled covariance-matrix ${\widehat{{\varvec{\Sigma}}}}_{{\varvec{\iota}}{\varvec{\iota}}}\left(f\right)$ via the unbiased *Hermitian Graphical LASSO* (**hgLASSO**) algorithm. Here, we also introduce a violation to the **hgLASSO** Hermitian graph elements in the functional connectivity MAP1 via the approximation of *Graphical LASSO* (**gLASSO**)^35,41^. This violation is incorporated in the second step for either **multistep** MAP1 inverse solutions with the first step via **ELORETA** (Fig. 7f1, f2) or **LCMV** (Fig. 7g1, g2). The sampled covariance matrix ${\widehat{{\varvec{\Sigma}}}}_{{\varvec{\iota}}{\varvec{\iota}}}\left(f\right)$ is determined from the first-step brain oscillations $\widehat{{\varvec{\iota}}}\left(f,t\right)$ defined as a filtered process^41^ and not its Hilbert transform^53^. To achieve a fair comparison, we employed the optimal regularization parameter for the initial inverse solutions **eLORETA** and **LCMV** selected by generalized cross-validation of the sampled covariance matrix for the data ${\widehat{{\varvec{\Sigma}}}}_{{\varvec{v}}{\varvec{v}}}\left(f\right)$.

The accuracy of the functional connectivity estimates was assessed by the classification scores of a binary ground true $\left\vert {{\varvec{\Theta}}}_{{\varvec{\iota}}{\varvec{\iota}}}\left(f\right)\right\vert$ (Fig. 7 left-center) by measures of the Receiver Operator Characteristic (ROC) (Fig. 7 right). These measures are given for inverse solutions $\left\vert {\widehat{{\varvec{\Theta}}}}_{{\varvec{\iota}}{\varvec{\iota}}}\left(f\right)\right\vert$ due to the planar (Fig. 7right-top) and human (Fig. 7right-bottom) EFMs. The measures used in the radar graphs were the global AUC (area under the curve) and partial SENS (sensitivity), SPEC (specificity), PREC (precision), and RECALL (F1 or Fisher scores) measured at the optimal operating point of the ROC curve.

### Confirmation of the performance of the functional connectivity for the HIGGS model in macaque EEG/ECoG experiments

**Figure 8:**
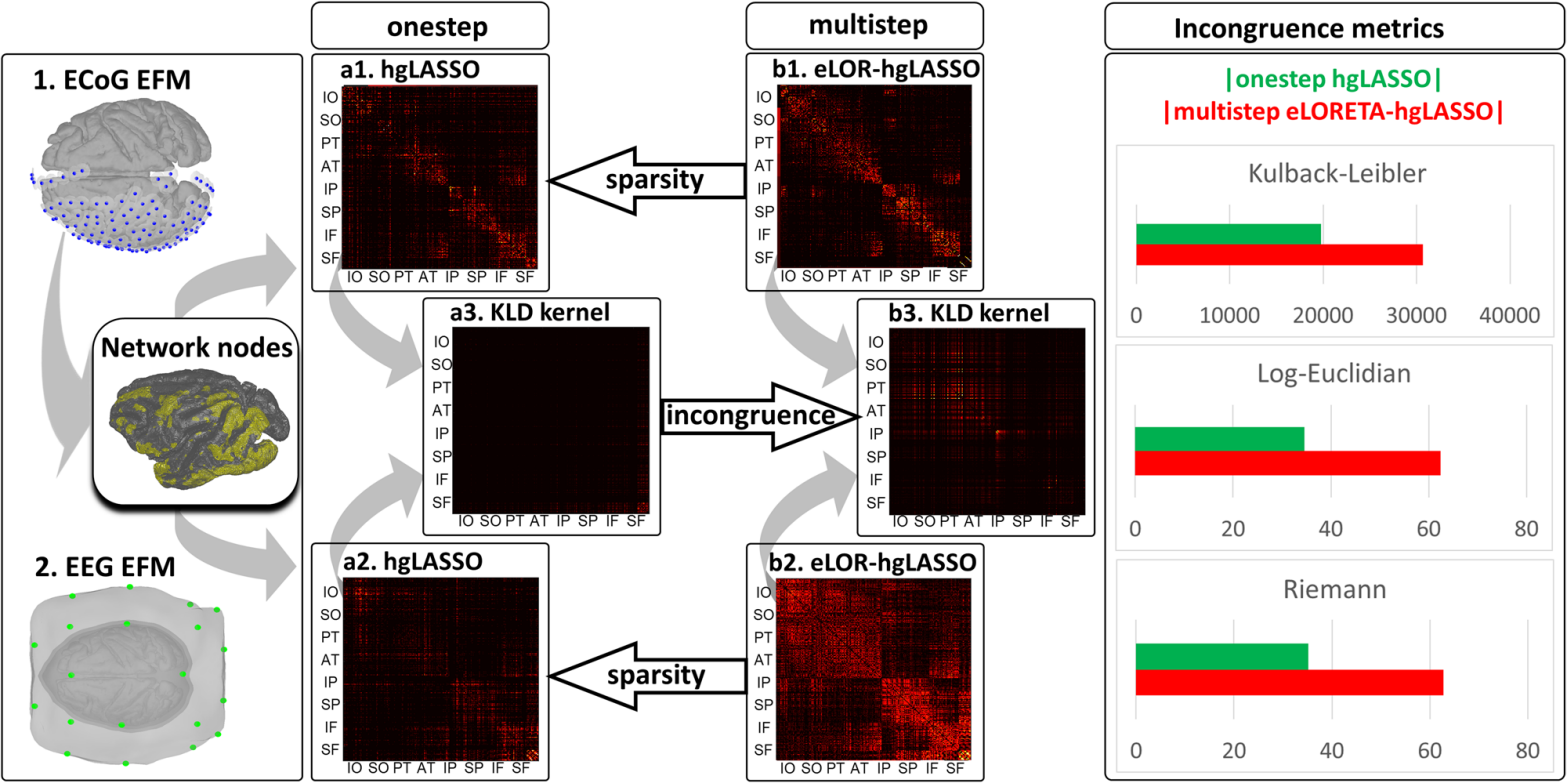
Experimental confirmation of performance in macaque simultaneous EEG/ECoG recordings (left). A large-scale alpha oscillatory network (left-center) screened from ECoG with certain method is the target of HIGGS inverse-solutions. Incongruence base on comparing these solutions under the ECOG (left-top) and EEG (left-bottom) EFM. Employing as reference the fine-grained ECOG solutions (**a1,b1**), incongruence of the EEG solutions are measured with Kulback–Leibler, LogEuclidean and Remaniean metrics (right) confirming the performance in Fig. 2. This incongruence due to leakage and localization errors is represented with the Kulback–Leibler divergence (KLD) kernel “EEG/ECoG+ EEG\ECoG– 2I” (**a3,b3**), for EEG and ECoG precision-matrices.

We leverage an experimental setup (Fig. 8) offering for macaque (1) large-scale coverage resting-state ECoG observations ${{\varvec{v}}}^{ECoG}\left(t,f\right)$ onto the left cortical hemisphere and (2) simultaneous EEG observations ${{\varvec{v}}}^{EEG}\left(t,f\right)$^123^. The ECoG observations ${{\varvec{v}}}^{ECoG}\left(t,f\right)$ are from a high-density subdural sensor array that was placed surgically onto the macaque cortex and provides (Fig. 8 left-top). See the experimental preparation and inverse solutions from ECoG ${\widehat{{\varvec{\Theta}}}}_{{\varvec{\iota}}{\varvec{\iota}}}^{ECoG}\left(f\right)$ and EEG ${\widehat{{\varvec{\Theta}}}}_{{\varvec{\iota}}{\varvec{\iota}}}^{EEG}\left(f\right)$ in Materials and Methods section "Validating performance for HIGGS inverse solutions", in this case targeting a functional network underlying the resting-state alpha rhythm.

On the one hand, a fine-grained EFM forward-operator ${\mathbf{L}}_{{\varvec{v}}{\varvec{\iota}}}^{ECoG}$ for the ECoG observations provides accurate functional network detection (Fig. 8 left-center), as well as functional connectivity by any **onestep** (Fig. 8a1) or **multistep** (Fig. 8b1) inverse solutions ${\widehat{{\varvec{\Theta}}}}_{{\varvec{\iota}}{\varvec{\iota}}}^{ECoG}\left(f\right)$. On the other hand, sensor low density and several tissue layers yield a coarse-grained EFM forward operator ${\mathbf{L}}_{{\varvec{v}}{\varvec{\iota}}}^{EEG}$ for the EEG observations (Fig. 8 left-bottom) and therefore as expected incongruent inverse solutions ${\widehat{{\varvec{\Theta}}}}_{{\varvec{\iota}}{\varvec{\iota}}}^{EEG}\left(f\right)$ (Fig. 8a2, b2) regarding an ECoG reference ${\widehat{{\varvec{\Theta}}}}_{{\varvec{\iota}}{\varvec{\iota}}}^{ECoG}\left(f\right)$ (Fig. 8a1, b1) as proposed in^125^.

We employ the *Kullback–Leibler Divergence* (KLD)^127^ as the proxy to measure this incongruence. The KLD distance measures the relative entropy between pairs of multivariate probability distributions. In this case, the distributions are *Gaussian Graphical Spectral* (GGS) models $p\left(\left.{\varvec{\iota}}\left(t,f\right)\right\vert {\widehat{{\varvec{\Theta}}}}_{{\varvec{\iota}}{\varvec{\iota}}}^{EEG}\left(f\right)\right)$ and $p\left(\left.{\varvec{\iota}}\left(t,f\right)\right\vert {\widehat{{\varvec{\Theta}}}}_{{\varvec{\iota}}{\varvec{\iota}}}^{ECoG}\left(f\right)\right)$ expressed as a function of the estimated precision matrices ${\widehat{{\varvec{\Theta}}}}_{{\varvec{\iota}}{\varvec{\iota}}}^{EEG}\left(f\right)$ and ${\widehat{{\varvec{\Theta}}}}_{{\varvec{\iota}}{\varvec{\iota}}}^{ECoG}\left(f\right)$ summarizing the GGS properties. A multivariate effect of distortions locally (for each graph element) for EEG ${\widehat{{\varvec{\Theta}}}}_{{\varvec{\iota}}{\varvec{\iota}}}^{EEG}\left(f\right)$ and regarding ECoG ${\widehat{{\varvec{\Theta}}}}_{{\varvec{\iota}}{\varvec{\iota}}}^{ECoG}\left(f\right)$ may be measured by means of the KLD kernel for **onestep** (Fig. 8a3) and **multistep** (Fig. 8b3) inverse solutions. For a quantitative analysis of the performance (Fig. 8 right), we report distances based on the KLD metric and others (Riemannian and Log-Euclid).

## Discussion

### Theory

The state-of-the-art connectivity inverse solution MEG-ROI-nets *Graphical LASSO* (**gLASSO**)^41^, which is employed here as a baseline here for comparison, was completely limited to only real-valued operations. The flaw is twofold due to instability in large scale and the lack of MEG-ROI-nets **gLASSO** algorithms capable of dealing with models that dwell in the complex-variable space of the Fourier or Hilbert transform under the GGS assumptions^30,45,80,81,83,87,128–131^. It was therefore our task to provide a solution to estimation errors produced by such algorithms as discussed in Introduction section "Second step MAP1 inverse solution for functional connectivity of the GGS model and estimation errors".

A **gLASSO** inverse solution based upon a real-valued sampled covariance ${\widehat{{\varvec{\Sigma}}}}_{{\varvec{\iota}}{\varvec{\iota}}}\left(f\right)$ for ${\varvec{\iota}}\left(t,f\right)$ defined as the filtered process for ${\varvec{\iota}}\left(t\right)$ or sometimes the Hilbert envelope $-log\left\vert {\varvec{\iota}}\left(t,f\right)\right\vert$ is claimed to encode in ${\widehat{{\varvec{\Theta}}}}_{{\varvec{\iota}}{\varvec{\iota}}}\left(f\right)$ all connectivity information in state-of-the-art neuroimaging practice but only does it partially. Here, we employed the MEG-ROI-nets **gLASSO**^41^ upon the real-valued sampled covariance matrix ${\widehat{{\varvec{\Sigma}}}}_{{\varvec{\iota}}{\varvec{\iota}}}\left(f\right)$ for the filtered process ${\varvec{\iota}}\left(t,f\right)$ which does not uphold the *Gaussian Graphical Spectral* (GGS) model assumptions but is a less gross approximation than Hilbert envelope $-log\left\vert {\varvec{\iota}}\left(t,f\right)\right\vert$^1,21,25–27,39–41,132,133^.

For oscillatory network phenomena estimation of connectivity ${\widehat{{\varvec{\Theta}}}}_{{\varvec{\iota}}{\varvec{\iota}}}\left(f\right)$ required both amplitude and phase information that was determined with the *Hermitian Graphical LASSO* (**hgLASSO**)^20^. We remind the reader a **hgLASSO** inverse solution introduced in *Materials and Methods *section "Gaussian graphical spectral (GGS) model and MAP1 inverse solution with Hermitian graphical LASSO (hgLASSO)"* and *section "GGS model MAP1 inverse solution via Hermitian graphical LASSO (hgLASSO) algorithm" is only resource to find estimates ${\widehat{{\varvec{\Theta}}}}_{{\varvec{\iota}}{\varvec{\iota}}}\left(f\right)$ for the complex-valued precision matrix of such GGS model. At every frequency, the complex-valued sampled covariance matrix ${\widehat{{\varvec{\Sigma}}}}_{{\varvec{\iota}}{\varvec{\iota}}}\left(f\right)$ for ${\varvec{\iota}}\left(t,f\right)$ defined as the Hilbert transform for ${\varvec{\iota}}\left(t\right)$ conforms to the so-called cross-spectrum. Only this quantity, or equivalently its matrix inverse or precision-matrix ${\widehat{{\varvec{\Theta}}}}_{{\varvec{\iota}}{\varvec{\iota}}}\left(f\right)$, fully encodes the dynamical properties of oscillatory components ${\varvec{\iota}}\left(t,f\right)$ in activity ${\varvec{\iota}}\left(t\right)$.

Our **hgLASSO** algorithm showed consistency in simulations to the theoretical Rayleigh tendency of amplitudes for the Hermitian graph-elements in complex-valued *Gaussian Graphical Spectral* (GGS) models (Fig. 6). Such Rayleigh tendency in Hermitian graph-elements^30,80,81^ was a natural extension of the Chi-Square tendency in the real-valued case^82,134^. The complexity of the proposed algorithm was bounded by the Hermitian matrix eigen decomposition and we shall note no **gLASSO** algorithm was available which fulfills statistical guarantees, performs stably in the large dimensionality, and deals with a complex-valued cross-spectrum. Our **hgLASSO** is therefore an optimal procedure amongst state-of the-art algorithms for this type of graphical model either in real or complex-variable^29,30,35,37,41,45,81–83,128,135,136^.

Estimation of the *Hidden GGS* (HIGGS) connectivity ${\widehat{{\varvec{\Theta}}}}_{{\varvec{\iota}}{\varvec{\iota}}}\left(f\right)$ in simulations (Fig. 7) and macaque real data (Fig. 8) as in typical real life situations assumed prescreened network nodes by some method determining the statistically significant cortical regions or nodes for a given resting state or task activity transient ${\varvec{\iota}}\left(t,f\right)$^38,40,41,137^. Implementing **onestep** and **multistep** connectivity estimates ${\widehat{{\varvec{\Theta}}}}_{{\varvec{\iota}}{\varvec{\iota}}}\left(f\right)$ in the subspace of prescreened network nodes ruled out other sources of distortions and allowed us to evaluate in isolation the estimation errors exposed in *Introduction *section "First step MAP1 inverse solution for brain oscillations and estimation errors". These estimation errors are caused in theory by misspecification of the HIGGS model in inverse solution targeting connectivity estimates ${\widehat{{\varvec{\Theta}}}}_{{\varvec{\iota}}{\varvec{\iota}}}\left(f\right)$. Other sources of distortion were the localization errors and leakage in activity estimates $\widehat{{\varvec{\iota}}}\left(t,f\right)$ that would be produced by **multistep** (in the first step) or **onestep** (successive approximations) upon the whole cortical space^38,41,42,54^.

Inverse solutions for HIGGS connectivity ${\widehat{{\varvec{\Theta}}}}_{{\varvec{\iota}}{\varvec{\iota}}}\left(f\right)$ were implemented via the **hgLASSO**, for both **onestep**, in successive approximations in an iterative fashion, and **multistep**, in a first step, estimating the cross-spectrum ${\widehat{{\varvec{\Sigma}}}}_{{\varvec{\iota}}{\varvec{\iota}}}\left(f\right)$ as described in *Materials and Methods *section "Hidden Gaussian graphical spectral (HIGGS) model and inverse solutions via multistep MAP1 and onestep MAP2". Our **onestep** connectivity inverse solution was an extension to the *Expectation Maximization* (EM) algorithm as with the identification of the covariance matrix ${\widehat{{\varvec{\Sigma}}}}_{{\varvec{\iota}}{\varvec{\iota}}}\left(f\right)$ in a *Covariance Component Model* (CCM)^73–76^ or identification of the autoregressive-coefficients ${\widehat{\mathbf{\rm K}}}_{{\varvec{\iota}}{\varvec{\iota}}}\left(f\right)$ in a *State Space Model* (SSM)^48,77,78^. Implementation of the **hgLASSO** algorithm defined the flavor of the HIGGS model **onestep** identification connectivity defined as the complex-valued precision matrix ${\widehat{{\varvec{\Theta}}}}_{{\varvec{\iota}}{\varvec{\iota}}}\left(f\right)$. HIGGS obtaining an unbiased, scalable and stable inverse solution for a locally optima GGS within EM iterations^88^ was not considered by previous CCM or SSM identification approaches.

### Validation

We spoused our validation of estimation errors in HIGGS inverse solutions in *electroencephalogram* (EEG) in simulations (Fig. 7) and macaque real data (Fig. 8), since this is the “worst case scenario” compared to *magnetoencephalogram* (MEG) or *electrocorticogram* (ECoG). EEG is exquisitely sensitive to conductivity heterogeneity of head tissue layers, which produces quite large distortions of the electric lead fields ${\mathbf{L}}_{{\varvec{v}}{\varvec{\iota}}}$^7–9^. Additionally, the high conductivity of the scalp leads to blurring of the EEG potential, which aggravates the estimation errors of any activity estimates $\widehat{{\varvec{\iota}}}\left(t,f\right)$ or its cross-spectrum ${\widehat{{\varvec{\Sigma}}}}_{{\varvec{\iota}}{\varvec{\iota}}}\left(f\right)$, that are transferred to connectivity estimates ${\widehat{{\varvec{\Theta}}}}_{{\varvec{\iota}}{\varvec{\iota}}}\left(f\right)$^138^. Thus, EEG results for connectivity ${\widehat{{\varvec{\Theta}}}}_{{\varvec{\iota}}{\varvec{\iota}}}\left(f\right)$ presented here can be considered a lower bound for those of MEG or ECoG.

The methods under evaluation fell into three different modalities: I) the **onestep** successive approximations, employing our **hgLASSO** unbiased estimator for ${\widehat{{\varvec{\Theta}}}}_{{\varvec{\iota}}{\varvec{\iota}}}\left(f\right)$ (Fig. 7 a1, a1), and employing estimators hgRidge (Fig. 7 b1, b2) and hgNaïve (Fig. 7 c1, c2) which violated this unbiasedness condition in ${\widehat{{\varvec{\Theta}}}}_{{\varvec{\iota}}{\varvec{\iota}}}\left(f\right)$. II) the **multistep** methods with **eLORETA**^56^ (Fig. 7 d1, d2) and **LCMV**^55^ (Fig. 7 e1, e2), both employing the same **hgLASSO** unbiased estimator for ${\widehat{{\varvec{\Theta}}}}_{{\varvec{\iota}}{\varvec{\iota}}}\left(f\right)$. It has been argued^53^ that these types of **multistep** estimators reduce connectivity estimation errors. III) the same **multistep** methods with **eLORETA** (Fig. 7 f1, f2) and **LCMV** (Fig. 7 g1, g2) but employing MEG-ROI-nets **gLASSO** estimator^35^ for the real-valued ${\widehat{{\varvec{\Theta}}}}_{{\varvec{\iota}}{\varvec{\iota}}}\left(f\right)$ implemented in the MEG-ROI-nets package^41^ which does not uphold the GGS assumptions.

Even with the relatively simple ground truth some methods produced large estimation errors in these simulations (Fig. 7) indistinctly, employing (I) an idealized or planar (Fig. 7 left top) and (II) a human (Fig. 7 left bottom) *Electromagnetic Forward Model* (EFM). In simulations (Fig. 7 right) partial classification measures RECALL, PREC, and SENS quantify and confirm the essential flaw with **multistep** methods employing (II) **hgLASSO** or (III) **gLASSO**. The perfect **multistep** estimation in the planar EFM (Fig. 7a1), which benefited from the renowned statistical goodness of eLORETA and our unbiased **hgLASSO** algorithm, was undone in the human EFM (Fig. 7a2). Another **multistep** solution based on LCMV (Fig. 7e1, e2), with excellent performance anywhere else, does not reach expectations even in the planar EFM ideal circumstances (Fig. 7e1). Only the **onestep hgLASSO** sparse inverse solution showed no major estimation errors (Fig. 7a1, a2) and outscores any other non-sparse **onestep** (Fig. 7b1, b2, c1, c2) or sparse **multistep** (Fig. 7d1, d2, e1, e2) solutions in both planar (right-top) and human (right-bottom) models.

Connectivity leakage measured as 20% in PREC or RECALL (shown as colormap false positives) is a systematic property of these **multistep** methods appearing even in a relatively simple human network model. The situation is even worse when using the **gLASSO** approximation for these methods, which corrects leakage in either simulation model (Fig. 7f1, f2, g1, g2) as claimed before^41,53^ but produces extreme estimation errors, yielding random classification according to all classification measures. **gLASSO** fails under the specific assumption of a GGS model, which bases connectivity on Hermitian graph elements. Previous simulation studies validating this correction method did not corroborate them under the GGS assumption and were limited to even simpler network models (up to 5 nodes)^41^.

The qualitatively sparse pattern (Fig. 7) obtained in simulations via the **onestep** (Fig. 7a1, a2) and **multistep** eLORETA (Fig. 7d1, d2), with both methods employing the unbiased **hgLASSO** inverse solution, resembled the sparsity simulated in the ground truth (Fig. 7 left center) and minimized connectivity estimation errors. Sparsity renders factual biological circumstances such as efficiency in large-scale network phenomena^139^, as we illustrated with the macaque ECoG (Fig. 8) with the sparse alpha network (Fig. 8 left center) and sparse connectivities (Fig. 8a1, b1) obtained from any (**onestep** or **multistep**) method employing the unbiased **hgLASSO** estimator.

Remarkably, the dimensionality of the macaque ECoG alpha network (Fig. 8 left-center) ruled out the possibility of using current implementations of the **gLASSO** algorithm implemented in^35,83,87^, or by MEG-ROI-nets^41^. Confirmation in macaque shows the connectivity determined for this type of network from the ECoG (Fig. 8 left-top) and EEG (Fig. 8 left-bottom) by means of the **onestep hgLASSO** (Fig. 8a1, a2) and **multistep eLORETA hgLASSO** (Fig. 8b1, b2).

The macaque experiment (Fig. 8) confirms the benefit in our **onestep hgLASSO** inverse solution, targeting a severely ill-posed inverse problem in recovering connectivities for a large-scale alpha network (Fig. 8 left-center) based only on EEG 19-sensor observations (Fig. 8 left-bottom). Indeed, for both the **onestep** and **multistep** methods, there are striking similarities in the ECoG (Fig. 8 a1, b2) and EEG (Fig. 8a2, b2) solutions. Our **onestep** hgLASSO solutions (Fig. 8a1, a2) maximize the sparsity. With the Kullback–Leibler divergence (KLD) kernel (Fig. 8a3, b3), we illustrate contraposition between these sparsity and distortion levels, measured as multivariate relative entropy for GGS models. This measure represents divergence (incongruence) from the identity matrix for the product between the EEG precision matrix and ECoG covariance that would express a multivariate effect of distortions locally (for each graph element). The norm for this matrix, the KLD metric and others (Riemannian and Log-Euclid) shows a 1/3 congruence improvement.

## Conclusion

In this manuscript, we leveraged Bayesian inverse problem theory to investigate the benefits and shortcomings in facing functional connectivity estimation with a large family of inverse solution methods. This estimation must be taken with care from mild to severely ill-conditioned and high-dimensional settings such as the ones dealt with here, in simulations and real data. In such settings, achieving exact functional connectivity estimates, via Bayesian MAP2 methods for the HIGGS model is unfeasible and requires approximations. We have introduced a reasonable approximation leading to the iterated MAP1s that is feasible, via quasilinear inverse solutions that incorporate our hgLASSO algorithm. Our HIGGS implementation specifying the hgLASSO a priori achieves the best performance due to unbiased functional connectivity estimates at every iteration, outperforming state-of-the-art methods. The Bayesian theory and methods presented here could potentially be applied to signal processing and imaging other biological phenomena described by the cross-spectrum.

## Supplementary Information

Supplementary Information.

## Data Availability

The original contributions presented in the study are included in the article/Supplementary material. Code, simulations, and data are also freely available, further inquiries can be directed to the corresponding author/s. https://github.com/CCC-members/MEEG_Source_Connectivity_SoftPack. https://github.com/CCC-members/BC-VARETA_Toolbox.
