# Supplementary material for: Identifying oscillatory brain networks with hidden Gaussian graphical spectral models of MEEG
Source: Sci Rep. 2023 Jul 15;13:11466. doi: 10.1038/s41598-023-38513-y (PMC10349891; doi:10.1038/s41598-023-38513-y)
Supplement: Supplementary file 1 — Supplementary Information. [file 41598_2023_38513_MOESM1_ESM.docx]

Identifying oscillatory brain networks with Hidden Gaussian Graphical Spectral models of MEEG

**Deirel Paz-Linares^1,2^, Eduardo Gonzalez-Moreira^1,3,6^, Ariosky Areces-Gonzalez^1,4^, Ying Wang^1^, Min Li^1^, Eduardo Martinez-Montes^2^, Jorge Bosch-Bayard^5,2^, Maria L. Bringas-Vega^1,2^, Mitchell Valdes-Sosa and Pedro A. Valdes-Sosa^1,2,*^**

**^1^** The Clinical Hospital of Chengdu Brain Science Institute, MOE Key Lab for Neuroinformation, University of Electronic Science and Technology of China, Chengdu, China. **^2^** Neuroinformatic Department, Cuban Neuroscience Center, Havana, Cuba. **^3^** Center for Research in Informatics, Central University “Marta Abreu” of Las Villas, Santa Clara, Cuba. **^4^** Faculty of Technical Sciences, University of Pinar del Río “Hermanos Saiz Montes de Oca”, Pinar del Rio, Cuba. **^5^** McGill Centre for Integrative Neurosciences MCIN, Ludmer Centre for Mental Health, Montreal Neurological Institute, McGill University, Montreal, Canada. **^6^** Research Unit in Neurodevelopment, Institute of Neurobiology, Autonomous University of Mexico, Querétaro, México

**^*^** corresponding author: Pedro A. Valdes-Sosa ([pedro.valdes@neuroinformatics-collaboratory.org](mailto:pedro.valdes@neuroinformatics-collaboratory.org))

# SUPPLEMENTARY INFORMATION

## Notation for variables and mathematical operations

|  | $x$, *or* $\boldsymbol{x}$, *or* $\mathbf{X}$, *or* $\mathbb{X}$ | Respectively denote a scalar $x$ (italic lowercase), or vector $\boldsymbol{x}$ (bold italic lowercase), or matrix $\mathbf{X}$ (bold capital), or set $\mathbb{X}$ (double struck capital). |
| --- | --- | --- |
|  | $\boldsymbol{x}\left( i \right)$, *or* $\mathbf{X}\left( i,j \right)$ | For a vector $\boldsymbol{x}$ or matrix $\mathbf{X}$ (as defined in I-1) the $i$-th, or $i,j$-th element. |
|  | $x\left( t \right)$, *or* $\boldsymbol{x}\left( t \right)$, *or* $\mathbf{X}\left( t \right)$, *and* $x\left( t,f \right)$, *or* $\boldsymbol{x}\left( t,f \right)$, *or* $\mathbf{X}\left( t,f \right)$ | A scalar $x$ function, or vector $\boldsymbol{x}$ function, or matrix $\mathbf{X}$ function (as defined in I-1) with argument $\left( t \right)$, and when these are a function of a second argument ($f$). |
|  | $\boldsymbol{x}\left( i,t \right)$, *or* $\mathbf{X}\left( i,j,t \right)$, *and*  $\boldsymbol{x}\left( i,t,f \right)$, *or* $\mathbf{X}\left( i,j,t,f \right)$ | The $i$-th, or $i,j$-th element (as defined in I-2) for (as defined in I-3) a vector $\boldsymbol{x}\left( t \right)$ function, or matrix function $\mathbf{X}\left( t \right)$ with argument $\left( t \right)$, and a vector function $\boldsymbol{x}\left( t,f \right)$, or matrix function $\mathbf{X}\left( t,f \right)$. |
|  | $\left\vert\mathbf{X} \right\vert$ | Determinant of (as defined in I-1) the matrix $\mathbf{X}$. |
|  | $tr\left( \mathbf{X} \right)$ | Trace of (as defined in I-1) the matrix $\mathbf{X}$. |
|  | $\mathbf{X}^{-1}$ | Inverse of (as defined in I-1) the matrix $\mathbf{X}$. |
|  | $\boldsymbol{x}^{⊺}$, *or* $\mathbf{X}^{⊺}$ | Transpose of (as defined in I-1) the vector $\boldsymbol{x}$, or matrix $\mathbf{X}$. |
|  | $\boldsymbol{x}^{\dagger}$, *or* $\mathbf{X}^{\dagger}$ | Conjugate transpose of (as defined in I-1) the complex-valued vector $\boldsymbol{x}$, or matrix $\mathbf{X}$. |
|  | $p\left( x \right)$, *or* $p\left( \boldsymbol{x} \right)$, *or* $p\left( \mathbf{X} \right)$ | Types of probability density functions for (as defined in I-1) a random scalar $x$, or random vector $\boldsymbol{x}$, or random matrix $\mathbf{X}$. |
|  | $p\left( x,y \right)$, *or* $p\left( \boldsymbol{x}\boldsymbol{,}\boldsymbol{y} \right)$, *or* $p\left( \mathbf{X}\mathbf{,}\mathbf{Y} \right)$, *and* $p\left( x,\boldsymbol{y} \right)$, *or* $p\left( x\boldsymbol{,}\mathbf{Y} \right)$, *or* $p\left( \boldsymbol{x}\mathbf{,}\mathbf{Y} \right)$ | Types of joint probability density functions for random scalars $x,y$, or random vectors $\boldsymbol{x}\boldsymbol{,}\boldsymbol{y}$, or random matrices $\mathbf{X}\mathbf{,}\mathbf{Y}$ (as defined in I-1 and I-10) as well as their possible combinations. |
|  | $p\left( x \vert y \right)$, *or* $p\left( \boldsymbol{x} \vert\boldsymbol{y} \right)$, *or* $p\left( \boldsymbol{x} \vert\mathbf{Y} \right)$, *and* $p\left( \mathbf{X} \vert y \right)$, *or* $p\left( \mathbf{X} \vert\mathbf{Y} \right)$ | Types of conditional probability density functions for random scalars $x,y$, or random vectors $\boldsymbol{x}\boldsymbol{,}\boldsymbol{y}$, or random matrices $\mathbf{X}\mathbf{,}\mathbf{Y}$ (as defined in I-1, I-10 and I-11) as well as their possible combinations. |
|  | $N\left( \boldsymbol{x} \vert\boldsymbol{\mu}_{\boldsymbol{x}},\boldsymbol{\Sigma}_{\boldsymbol{x}} \right)$, *or* $N\left( \boldsymbol{x} \vert\boldsymbol{\mu}_{\boldsymbol{x}},\boldsymbol{\Theta}_{\boldsymbol{x}}^{-1} \right)$ | Real-valued Gaussian distribution for a vector $\boldsymbol{x}$ conditioned to (as defined in I-12) the mean $\boldsymbol{\mu}$ and real valued symmetric ensemble covariance-matrix $\boldsymbol{\Sigma}_{\boldsymbol{x}}$ or precision-matrix $\boldsymbol{\Theta}_{\boldsymbol{x}}$ (as defined in I-12). |
|  | $N^{\mathbb{C}}\left( \boldsymbol{x} \vert\boldsymbol{\mu}_{\boldsymbol{x}},\boldsymbol{\Sigma}_{\boldsymbol{x}} \right)$, *or* $N^{\mathbb{C}}\left( \boldsymbol{x} \vert\boldsymbol{\mu}_{\boldsymbol{x}},\boldsymbol{\Theta}_{\boldsymbol{x}}^{-1} \right)$ | Hermitian (complex-valued circularly symmetric) Gaussian distribution for a vector $\boldsymbol{x}$ with conditioned to (as defined in I-12) mean $\boldsymbol{\mu}_{\boldsymbol{x}}$ and complex valued hermitian ensemble covariance-matrix $\boldsymbol{\Sigma}_{\boldsymbol{x}}$ or precision-matrix $\boldsymbol{\Theta}_{\boldsymbol{x}}$. |
|  | $\exp\left( x \vert\alpha\right)$ | Exponential distribution for the scalar $x$ conditioned to (as defined in I-12) the scalar parameter of shape $\alpha$. |
|  | $Ga \left( x \vert\rho,\alpha\right)$ | Gamma distribution for the scalar $x$ conditioned (as defined in I-12) to the scalar parameters of shape $\rho$ and rate $\alpha$. |
|  | $\hat{x}$, *or* $\hat{\boldsymbol{x}}$*, or*  $\hat{\mathbf{X}}$***,*** *and,* $\check{x}$, *or* $\check{\boldsymbol{x}}$*, or* $\check{\mathbf{X}}$ | Estimator (hat) for (as defined in I-1) a scalar $x$, or vector $\boldsymbol{x}$, or matrix $\mathbf{X}$, also when these estimators denote a class of auxiliarly quantities (inverted hat). |
|  | $\hat{x}^{\left( k \right)}$, *or* ${\hat{\boldsymbol{x}}}^{\left( k \right)}$, *or* ${\hat{\mathbf{X}}}^{\left( k \right)}$, *and* $\check{x}^{\left( k \right)}$, *or* ${\check{\boldsymbol{x}}}^{\left( k \right)}$, ${\check{\mathbf{X}}}^{\left( k \right)}$ | The estimators (as defined in 4-14) given in the $k$-th iteration within a loop. |
|  | $\sum_{t\in\mathbb{T}}$, $\sum_{t=1}^{T}$ | Sum operator along index $t$, valid for $i,j,t,f$ in matrices or vectors (as defined in 4.4) that can be defined within a countable set $\mathbb{T}$ (as defined in 4-1) or from $1$ up to a maximum $T$. |
|  | $\prod_{t\in\mathbb{T}}$, $\prod_{t=1}^{T}$ | Product operator along index $t$, valid for $i,j,t,f$ in matrices or vectors (as defined in 4.4) that can be defined within a countable set $\mathbb{T}$ (as defined in 4-1) or from $1$ up to a maximum $T$. |
|  | $\left\Vert\boldsymbol{x} \right\Vert_{n}$, $n=1,2$, *or* $\left\Vert\mathbf{X} \right\Vert_{n}$, $n=1,2$ | L1 or L2 norms of the vector $\boldsymbol{x}$ or matrix $\mathbf{X}$. |
|  | $\mathbf{I}$, $\boldsymbol{1}$, $\boldsymbol{0}$ | Respectively Identity, Ones and Ceros matrices. |
|  | $\boldsymbol{\odot}$**,** $⊘$ | Elementwise (Hadamard) product a division operators for matrices or vectors. |
|  | ${argmin}_{\mathbf{X}}\left\{ f\left( \mathbf{X} \right) \right\}$ or ${argmax}_{\mathbf{X}}\left\{ f\left( \mathbf{X} \right) \right\}$ | Extreme values of the scalar function $f$, correspondingly minimum or maximum, in the matrix argument $\mathbf{X}$ , valid for a scalar $x$ or a vector $\boldsymbol{x}$. |
|  | ${zeros}_{\mathbf{X}}\left\{ f\left( \mathbf{X} \right) \right\}$ | Zeros of the scalar function $f$ in the matrix argument $\mathbf{X}$, valid for a scalar $x$ or a vector $\boldsymbol{x}$. |

## Nomenclature for theoretical quantities

|  | $\mathbb{E}$, *with* $E=\left\vert\mathbb{E} \right\vert$ *and* $e\in\mathbb{E}$ | Physical space of MEG/EEG/ECoG observations $\mathbb{E}$, with size $E$, sensors in this space are defined as $e\in\mathbb{E}$. |
| --- | --- | --- |
|  | $\mathbb{G}$, *with* $G=\left\vert\mathbb{G} \right\vert$ *and* $g\in\mathbb{G}$ | Physical space of local neural currents or cortical generators of the MEG/EEG/ECoG $\mathbb{G}$, with size $G$, cortical generators in this space are defined as $g\in\mathbb{G}$. |
|  | $\mathbb{T}$, *with* $T=\left\vert\mathbb{T} \right\vert$ *and* $t\in\mathbb{T}$ | Domain of time for processes, local neural currents or MEG/EEG/ECoG observations and their perturbations, $\mathbb{T}$ with size $T$, instances in this space are defined as $t\in\mathbb{T}$. |
|  | $\mathbb{F}$, *with* $F=\left\vert\mathbb{F} \right\vert$ *and* $f\in\mathbb{F}$ | Domain of frequency for spectral processes, spectral local neural currents or spectral MEG/EEG/ECoG observations and their perturbations, $\mathbb{F}$ with size $F$, instances in this space are defined as $f\in\mathbb{F}$. |
|  | $\boldsymbol{v}\left( t \right)$, *and* $\boldsymbol{v}\left( t,f \right)$ | MEG/EEG/ECoG observations $\boldsymbol{v}\left( t \right)$, in time $\mathbb{T}$ (as defined in II-3), and its Hilbert transform, MEG/EEG/ECoG spectral observations or MEG/EEG/ECoG oscillations $\boldsymbol{v}\left( t,f \right)$, in time $\mathbb{T}$ (as defined in II-3) and frequency $\mathbb{F}$ (as defined in II-4). Either vectors are defined in the space $\mathbb{E}$ (as defined in 5-1). |
|  | $\boldsymbol{\xi}\left( t \right)$, *and* $\boldsymbol{\xi}\left( t,f \right)$ | For the MEG/EEG/ECoG observations $\boldsymbol{v}\left( t \right)$ (as defined in II-5) an instrumental noise process $\boldsymbol{\xi}\left( t \right)$, and its Hilbert transform or spectral instrumental noise $\boldsymbol{\xi}\left( t,f \right)$. |
|  | $\boldsymbol{\iota}\left( t \right)$, *and* $\boldsymbol{\iota}\left( t,f \right)$ | Local neural currents or cortical activity $\boldsymbol{\iota}\left( t \right)$, in time $\mathbb{T}$ (as defined in II-3), and its Hilbert transform, cortical spectral activity or cortical oscillations $\boldsymbol{\iota}\left( t,f \right)$, in time $\mathbb{T}$ (as defined in II-3) and frequency $\mathbb{F}$ (as defined in II-4). Either vectors are defined in the space $\mathbb{E}$ (as defined in II-1). |
|  | $\boldsymbol{\zeta}\left( t \right)$, *and* $\boldsymbol{\zeta}\left( t,f \right)$ | For the cortical activity $\boldsymbol{\iota}\left( t \right)$ (as defined in II-7) a biological noise process $\boldsymbol{\zeta}\left( t \right)$, and its Hilbert transform or spectral biological noise $\boldsymbol{\zeta}\left( t,f \right)$. |
|  | $\mathbf{L}$, *with* $\boldsymbol{v}\left( t \right)=\mathbf{L}\boldsymbol{\iota}\left( t \right)+\boldsymbol{\xi}\left( t \right)$, *or* $\boldsymbol{v}\left( t,f \right)=\mathbf{L}\boldsymbol{\iota}\left( t,f \right)+\boldsymbol{\xi}\left( t,f \right)$ | Lead Field of the Electromagnetic Forward Model for MEG/EEG/ECoG $\mathbf{L}$, an $E\times G$-size matrix in the space product $\mathbb{E}\times\mathbb{G}$ (as defined in II-1 and II-2). With the observations $\boldsymbol{v}\left( t \right)$ or spectral observations $\boldsymbol{v}\left( t,f \right)$ (as defined in II-5) produced from cortical activity $\boldsymbol{\iota}\left( t \right)$ or spectral cortical activity $\boldsymbol{\iota}\left( t,f \right)$ (as defined in II-7). |
|  | $\mathbf{T}_{\boldsymbol{\xi v}}$*, with* $\hat{\boldsymbol{\xi}}\left( t,f \right)=\mathbf{T}_{\boldsymbol{\xi v}}\boldsymbol{v}\left( t,f \right)$ | Inverse operator $\mathbf{T}_{\boldsymbol{\xi v}}$ estimating (hat) the spectral instrumental noise $\hat{\boldsymbol{\xi}}\left( t,f \right)$ (as defined in 5-6) from MEG/EEG/ECoG spectral observations $\boldsymbol{v}\left( t,f \right)$ (as defined in 5-5), $E\times E$-size matrix in the space product $\mathbb{E}\times\mathbb{E}$ (as defined in 5-1). |
|  | $\mathbf{T}_{\boldsymbol{\iota v}}$*, with* $\hat{\boldsymbol{\iota}}\left( t,f \right)=\mathbf{T}_{\boldsymbol{\iota v}}\boldsymbol{v}\left( t,f \right)$ | Inverse operator $\mathbf{T}_{\boldsymbol{\iota v}}$ estimating (hat) the spectral cortical activity or cortical oscillations $\hat{\boldsymbol{\iota}}\left( t,f \right)$ (as defined in 5-7) from MEG/EEG/ECoG spectral observations $\boldsymbol{v}\left( t,f \right)$ (as defined in 5-5), $G\times E$-size matrix in the space product $\mathbb{G}\times\mathbb{E}$ (as defined in 5-1 and 5-2). |
|  | $\boldsymbol{\Sigma}_{\boldsymbol{v}}\left( f \right)$, *and* $\boldsymbol{\Sigma}_{\boldsymbol{\xi}}\left( f \right)$, *and* $\boldsymbol{\Sigma}_{\boldsymbol{\iota}}\left( f \right)$, *and* $\boldsymbol{\Sigma}_{\boldsymbol{\zeta}}\left( f \right)$ | Ensemble covariance-matrix $\boldsymbol{\Sigma}$ for a process at a given frequency $f\in\mathbb{F}$ and for all time instances $t\in\mathbb{T}$. Respectively for the MEG/EEG/ECoG spectral observations $\boldsymbol{v}\left( t,f \right)$ (as defined in 5-5), the spectral instrumental noise $\boldsymbol{\xi}\left( t,f \right)$ (as defined in 5-6), the spectral activity $\boldsymbol{\iota}\left( t,f \right)$ (as defined in 5-7), and the spectral biological noise $\boldsymbol{\zeta}\left( t,f \right)$ (as defined in 5-8). |
|  | ${\hat{\mathbf{S}}}_{\boldsymbol{v}}\left( f \right)$, *and* ${\hat{\boldsymbol{\Sigma}}}_{\boldsymbol{\xi}}\left( f \right)⊺$, *and* ${\hat{\boldsymbol{\Sigma}}}_{\boldsymbol{\iota}}\left( f \right)$, *and* ${\hat{\boldsymbol{\Sigma}}}_{\boldsymbol{\zeta}}\left( f \right)$ | Sampled covariance-matrix $\hat{\boldsymbol{\Sigma}}$ for a process at a given frequency $f\in\mathbb{F}$ and for all time instances $t\in\mathbb{T}$. Respectively for the MEG/EEG/ECoG spectral observations $\boldsymbol{v}\left( t,f \right)$ (as defined in 5-5), the spectral instrumental noise $\boldsymbol{\xi}\left( t,f \right)$ (as defined in 5-6), the spectral activity $\boldsymbol{\iota}\left( t,f \right)$ (as defined in 5-7), and the spectral biological noise $\boldsymbol{\zeta}\left( t,f \right)$ (as defined in 5-8). |
|  | ${\bar{\boldsymbol{\Sigma}}}_{\boldsymbol{\xi}}\left( f \right)$, *and* ${\bar{\boldsymbol{\Sigma}}}_{\boldsymbol{\iota}}\left( f \right)$ | First type maximum a posteriori estimator (bar $\bar{\boldsymbol{\Sigma}}$) of a covariance-matrix $\boldsymbol{\Sigma}$ (as defined in 5-12), and which if dependent of the second type maximum a posteriori estimators. Respectively, for the spectral instrumental noise $\boldsymbol{\xi}\left( t,f \right)$ (as defined in 5-6), spectral activity $\boldsymbol{\iota}\left( t,f \right)$ (as defined in 5-7). |
|  | ${\bar{\boldsymbol{\Sigma}}}_{\boldsymbol{\xi}}\left( f \right)$, *and* ${\bar{\boldsymbol{\Sigma}}}_{\boldsymbol{\iota}}\left( f \right)$ | First type maximum a posteriori estimator (bar $\bar{\boldsymbol{\Sigma}}$) of a covariance-matrix $\boldsymbol{\Sigma}$ (as defined in 5-12), and which if dependent of the second type maximum a posteriori estimators. Respectively, for the spectral instrumental noise $\boldsymbol{\xi}\left( t,f \right)$ (as defined in 5-6), spectral activity $\boldsymbol{\iota}\left( t,f \right)$ (as defined in 5-7). |
|  | ${\check{\mathbf{Z}}}_{\boldsymbol{\xi\xi}}\left( f \right)$, *and* ${\check{\boldsymbol{\Psi}}}_{\boldsymbol{\iota\iota}}\left( f \right)$ | Auxiliarly estimator (inverted hat $\check{\boldsymbol{\Psi}}$) Second type maximum a posteriori effective estimator of the covariance-matrices (as defined in 5-12) for the spectral instrumental noise $\boldsymbol{\xi}\left( t,f \right)$ (as defined in 5-6), spectral activity $\boldsymbol{\iota}\left( t,f \right)$ (as defined in 5-7). This estimation depends on the sampled estimator (as defined in 5-13) and a posteriori estimator (as defined in 5-14). |
|  | $\boldsymbol{\Theta}_{\boldsymbol{vv}}\left( f \right)$, *and* $\boldsymbol{\Theta}_{\boldsymbol{\xi\xi}}\left( f \right)$, *and* $\boldsymbol{\Theta}_{\boldsymbol{\iota\iota}}\left( f \right)$, *and* $\boldsymbol{\Theta}_{\boldsymbol{\zeta\zeta}}\left( f \right)$ | Ensemble precision-matrices $\boldsymbol{\Theta}$ (covariance-matrix inverse $\boldsymbol{\Sigma}^{\boldsymbol{-}\boldsymbol{1}}$, as defined in 5-12) in the time domain $t$ for the spectral observations $\boldsymbol{v}\left( t,f \right)$ (as defined in 5-5), spectral instrumental noise $\boldsymbol{\xi}\left( t,f \right)$ (as defined in 5-6), spectral activity $\boldsymbol{\iota}\left( t,f \right)$ (as defined in 5-7), and spectral biological noise $\boldsymbol{\zeta}\left( t,f \right)$ (as defined in 5-8). |
|  | ${\hat{\boldsymbol{\Theta}}}_{\boldsymbol{\xi\xi}}\left( f \right)$, *with* ${\hat{\boldsymbol{\Theta}}}_{\boldsymbol{\xi\xi}}\left( f \right)=\mathbf{W}_{\boldsymbol{\xi\xi}}\left( {\hat{\boldsymbol{\Sigma}}}_{\boldsymbol{\xi\xi}}\left( f \right) \right)$ *or* ${\hat{\boldsymbol{\Theta}}}_{\boldsymbol{\xi\xi}}\left( f \right)=\mathbf{W}_{\boldsymbol{\xi\xi}}\left( {\check{\boldsymbol{\Psi}}}_{\boldsymbol{\xi\xi}}\left( f \right) \right)$*, and* ${\hat{\boldsymbol{\Theta}}}_{\boldsymbol{\iota\iota}}\left( f \right)$, *with* ${\hat{\boldsymbol{\Theta}}}_{\boldsymbol{\iota\iota}}\left( f \right)=\mathbf{W}_{\boldsymbol{\iota\iota}}\left( {\hat{\boldsymbol{\Sigma}}}_{\boldsymbol{\iota\iota}}\left( f \right) \right)$ *or* ${\hat{\boldsymbol{\Theta}}}_{\boldsymbol{\iota\iota}}\left( f \right)=\mathbf{W}_{\boldsymbol{\iota\iota}}\left( {\check{\boldsymbol{\Psi}}}_{\boldsymbol{\iota\iota}}\left( f \right) \right)$ | Estimator (hat $\hat{\boldsymbol{\Theta}}$) of the precision-matrices (as defined in 5-16) for the spectral instrumental noise $\boldsymbol{\xi}\left( t,f \right)$ (as defined in 5-6), spectral activity $\boldsymbol{\iota}\left( t,f \right)$ (as defined in 5-7). This estimation is via an inverse operator via an inverse-operator $\mathbf{W}$ from the sampled covariance-matrix estimators (hat $\hat{\boldsymbol{\Sigma}}$) (as defined in 5-13) or the efective covariance-matrix estimators (inverted hat $\check{\boldsymbol{\Psi}}$) (as defined in 5-15). |
|  | $\boldsymbol{\sigma}^{\boldsymbol{2}}\left( f \right)\mathbf{=}diag\left( \boldsymbol{\Sigma}\left( f \right) \right)$, *or* $\boldsymbol{\theta}^{\boldsymbol{2}}\left( f \right)\mathbf{=}diag\left( \boldsymbol{\Theta}\left( f \right) \right)$ | Variances $\boldsymbol{\sigma}^{\boldsymbol{2}}$ from the covariance-matrices $\boldsymbol{\Sigma}$ (as defined 5-12) or precisions from the precision-matrices $\boldsymbol{\Theta}$ (as defined in 5-16). The same notation is extensible to the estimators (as defined in 5-14, 5-15 and 5-17). Special cases are the variances (or precisions) for the spectral instrumental noise $\boldsymbol{\sigma}_{\boldsymbol{\xi}}^{2}$ (or $\boldsymbol{\theta}_{\boldsymbol{\xi}}^{2}$) and biological noise $\boldsymbol{\sigma}_{\boldsymbol{\zeta}}^{2}$ (or $\boldsymbol{\theta}_{\boldsymbol{\zeta}}^{2}$). |
|  | $\Pi\left( \boldsymbol{A⨀\Theta}\left( f \right) \right)$ | Penalization function or exponent of the precision-matrix $\boldsymbol{\Theta}$ (as defined in 5-16) Gibbs prior parametrized in the regularization parameters or mask matrix $\mathbf{A}$. As a penalization function $\Pi$ we may use the hermitian graphical LASSO (hgLASSO) $\Pi=\left\Vert\cdot\right\Vert_{1}$, hermitian graphical Ridge (hgRidge) $\left\Vert\cdot\right\Vert_{2}^{2}$, or hermitian graphical Naïve $\Pi=0$. |
|  | $\boldsymbol{\Omega}\left( f \right)$*, and* $\hat{\boldsymbol{\Omega}}\left( f \right)$ | Set of hyperparameters $\boldsymbol{\Omega}$ and its estimator (hat $\hat{\boldsymbol{\Omega}}$) in a model. This set may be conformed by the covariance-matrices $\boldsymbol{\Sigma}$ (as defined in 5-12) or precision-matrices $\boldsymbol{\Theta}$ (as defined in 5-16). |
|  | $Q\left( \boldsymbol{\Omega}\left( f \right)\mathbf{,}\hat{\boldsymbol{\Omega}}\left( f \right) \right)$ | Expected $-log$ second-type likelihood of the data $\boldsymbol{v}\left( t,f \right)$ (as defined in 5-5), obtained after the expectation operation upon parameters $\boldsymbol{\iota}\left( t,f \right)$ (as defined in 5-7) of the data $\boldsymbol{v}\left( t,f \right)$ and parameters $\boldsymbol{\iota}\left( t,f \right)$ $-log$ joint conditional probability density function over the hyperparameters $\boldsymbol{\Omega}\left( f \right)$ (as defined in 5-20). the expectarion is based on the posterior distribution for the parameters $\boldsymbol{\iota}\left( t,f \right)$ parametrized in the estimated hyperparameters $\hat{\boldsymbol{\Omega}}\left( f \right)$. |
|  | $\mathcal{L}\left( \boldsymbol{\Omega}\left( f \right) \right)$ | Additive combination of $Q\left( \boldsymbol{\Omega}\left( f \right)\mathbf{,}\hat{\boldsymbol{\Omega}}\left( f \right) \right)$ (as defined in 5-21) and the $-log$ prior for the hyperparameters $\boldsymbol{\Omega}\left( f \right)$ (as defined in 5-20). |

## Bimodal Wishart form of the HIGGS expected second-type likelihood (Lemma 1)

***Lemma 1:*** *For a three-level Bayesian hierarchy due to the Hidden Gaussian Graphical Spectral (HIGGS) model (equation III-1) conditionally dependent to the precision-matrices (hyperparameters)* $\boldsymbol{\Omega}=\left\{ \boldsymbol{\Theta}_{\boldsymbol{\iota\iota}},\boldsymbol{\Theta}_{\boldsymbol{\xi\xi}} \right\}$

$\begin{matrix} p\left( \left. \boldsymbol{v}\left( t \right) \right|\boldsymbol{\iota}\left( t \right),\boldsymbol{\Theta}_{\boldsymbol{\xi\xi}} \right)=N_{p}^{\mathbb{C}}\left( \boldsymbol{v}\left( t \right) | \mathbf{L}_{\boldsymbol{v\iota}}\boldsymbol{\iota}\left( t \right),\boldsymbol{\Theta}_{\boldsymbol{\xi\xi}}^{-1} \right) \\ p\left( \left. \boldsymbol{\iota}\left( t \right) \right|\boldsymbol{\Theta}_{\boldsymbol{\iota\iota}} \right)=N_{p}^{\mathbb{C}}\left( \boldsymbol{\iota}\left( t \right) | \mathbf{0},\boldsymbol{\Theta}_{\boldsymbol{\iota\iota}}^{-1} \right) \end{matrix}$

*a local approximation to the intractable second-type likelihood* $p\left( \left. {\hat{\boldsymbol{\Sigma}}}_{\boldsymbol{vv}} \right|\boldsymbol{\Omega} \right)$*, which explains the observed cross-spectrum, sampled covariance-matrix* ${\hat{\boldsymbol{\Sigma}}}_{\boldsymbol{vv}}$ *(equation III-2) in terms of these hyperparameters* $\boldsymbol{\Omega}$*, at every iteration* $k$*-th of the Expectation Maximization (EM) algorithm*

${\hat{\boldsymbol{\Sigma}}}_{\boldsymbol{vv}}=\frac{1}{T}\sum_{t\mathbb{\in T}} \boldsymbol{v}\left( t \right){\boldsymbol{v}\left( t \right)}^{\dagger}$

*denominated expected second-type likelihood* $p^{\left( k \right)}\left( {\hat{\boldsymbol{\Sigma}}}_{\boldsymbol{vv}} | \boldsymbol{\Omega} \right)$*,* *admits a decomposition into two locally independent Wishart factors, a bimodal Wishart form (equation III-3) in terms of the effective sampled covariance-matrices* ${\check{\boldsymbol{\Psi}}}_{\boldsymbol{\iota\iota}}^{\left( k \right)}$ *and* ${\check{\boldsymbol{\Psi}}}_{\boldsymbol{\xi\xi}}^{\left( k \right)}$

$\begin{matrix} p^{\left( k \right)}\left( {\hat{\boldsymbol{\Sigma}}}_{\boldsymbol{vv}} | \boldsymbol{\Omega} \right)=p^{\left( k \right)}\left( \left. {\hat{\boldsymbol{\Sigma}}}_{\boldsymbol{vv}} \right|\boldsymbol{\Theta}_{\boldsymbol{\iota\iota}} \right)p^{\left( k \right)}\left( \left. {\hat{\boldsymbol{\Sigma}}}_{\boldsymbol{vv}} \right|\boldsymbol{\Theta}_{\boldsymbol{\xi\xi}} \right) \\ p^{\left( k \right)}\left( \left. {\hat{\boldsymbol{\Sigma}}}_{\boldsymbol{vv}} \right|\boldsymbol{\Theta}_{\boldsymbol{\iota\iota}} \right)\boldsymbol{=}W_{q}^{\mathbb{C}}\left( {\check{\boldsymbol{\Psi}}}_{\boldsymbol{\iota\iota}}^{\left( k \right)} | T^{-1}\boldsymbol{\Theta}_{\boldsymbol{\iota\iota}}^{-1},T \right) \\ p^{\left( k \right)}\left( \left. {\hat{\boldsymbol{\Sigma}}}_{\boldsymbol{vv}} \right|\boldsymbol{\Theta}_{\boldsymbol{\xi\xi}} \right)\boldsymbol{=}W_{p}^{\mathbb{C}}\left( {\check{\boldsymbol{\Psi}}}_{\boldsymbol{\xi\xi}}^{\left( k \right)} | T^{-1}\boldsymbol{\Theta}_{\boldsymbol{\xi\xi}}^{-1},T \right) \end{matrix}$

*due to the Gibbs exponential form expressing* $p^{\left( k \right)}\left( {\hat{\boldsymbol{\Sigma}}}_{\boldsymbol{vv}} | \boldsymbol{\Omega} \right)$ *as function of the expected* $-log$ *second-type likelihood from the EM integral operation* $Q\left( \boldsymbol{\Omega},{\hat{\boldsymbol{\Omega}}}^{\left( k \right)} \right)$ *(equation III-4) which may be expressed explicitly as an additive functions of* $\boldsymbol{\Theta}_{\boldsymbol{\iota\iota}}$ *and* $\boldsymbol{\Theta}_{\boldsymbol{\xi\xi}}$ *(equation III-5)*

$\begin{matrix} p^{\left( k \right)}\left( {\hat{\boldsymbol{\Sigma}}}_{\boldsymbol{vv}} | \boldsymbol{\Omega} \right)=exp\left( \left. Q\left( \boldsymbol{\Omega},{\hat{\boldsymbol{\Omega}}}^{\left( k \right)} \right) \right|1 \right) \\ \begin{matrix} Q\left( \boldsymbol{\Omega},{\hat{\boldsymbol{\Omega}}}^{\left( k \right)} \right)=-\sum_{t\mathbb{\in T}} E\left( log\left( p\left( \boldsymbol{v}\left( t \right),\boldsymbol{\iota}\left( t \right) | \boldsymbol{\Omega} \right) \right) | \boldsymbol{v}\left( t \right)\mathbf{,}{\hat{\boldsymbol{\Omega}}}^{\left( k \right)} \right) \\ E\left( log\left( p\left( \boldsymbol{v}\left( t \right),\boldsymbol{\iota}\left( t \right) | \boldsymbol{\Omega} \right) \right) | \boldsymbol{v}\left( t \right)\mathbf{,}{\hat{\boldsymbol{\Omega}}}^{\left( k \right)} \right)=\underset{\mathbb{C}^{q}}{\int}log\left( p\left( \boldsymbol{v}\left( t \right),\boldsymbol{\iota}\left( t \right) | \boldsymbol{\Omega} \right) \right) p\left( \boldsymbol{\iota}\left( t \right) | \boldsymbol{v}\left( t \right)\mathbf{,}{\hat{\boldsymbol{\Omega}}}^{\left( k \right)} \right)d\boldsymbol{\iota}\left( t \right) \end{matrix} \end{matrix}$

*where the explicit* $Q\left( \boldsymbol{\Omega},{\hat{\boldsymbol{\Omega}}}^{\left( k \right)} \right)$ *(equation III-5) is*

$Q\left( \boldsymbol{\Omega},{\hat{\boldsymbol{\Omega}}}^{\left( k \right)} \right)=-T\log\left| \boldsymbol{\Theta}_{\boldsymbol{\iota\iota}} \right|+T tr\left( {\check{\boldsymbol{\Psi}}}_{\boldsymbol{\iota\iota}}^{\left( k \right)}\boldsymbol{\Theta}_{\boldsymbol{\iota\iota}} \right)-T\log\left| \boldsymbol{\Theta}_{\boldsymbol{\xi\xi}} \right|+T tr\left( {\check{\boldsymbol{\Psi}}}_{\boldsymbol{\xi\xi}}^{\left( k \right)}\boldsymbol{\Theta}_{\boldsymbol{\xi\xi}} \right)$

***Proof of Lemma 1***

The general strategy for the identification of the HIGGS model (equation III-1) is based on the EM algorithm: a second-type maximum a posteriori estimation, performed iteratively for $\boldsymbol{\iota}\left( t \right)$ (parameters) and $\boldsymbol{\Omega}$ (hyperparameters) [1]–[4]. The EM expectation obtains a second-type likelihood (equation III-3) based on the posterior distribution of the parameters $p\left( \boldsymbol{\iota}\left( t \right) | \boldsymbol{v}\left( t \right)\mathbf{,}{\hat{\boldsymbol{\Omega}}}^{\left( k \right)} \right)$ (equation III-4) expressed upon fixed values of ${\hat{\boldsymbol{\Omega}}}^{\left( k \right)}$ (hyperparameters).

This posterior distribution is proportional to the factor of the first-type likelihood $N_{p}^{\mathbb{C}}\left( \boldsymbol{v}\left( t \right) | \mathbf{L}_{\boldsymbol{v\iota}}\boldsymbol{\iota}\left( t \right),{{\hat{\boldsymbol{\Theta}}}_{\boldsymbol{\xi\xi}}^{\left( k \right)}}^{-1} \right)$ and the prior $N_{p}^{\mathbb{C}}\left( \boldsymbol{\iota}\left( t \right) | \mathbf{0},{{\hat{\boldsymbol{\Theta}}}_{\boldsymbol{\iota\iota}}^{\left( k \right)}}^{-1} \right)$ (equation III-1) due to the Bayes theorem por conditional probabilities.

$p\left( \boldsymbol{\iota}\left( t \right) | \boldsymbol{v}\left( t \right)\mathbf{,}\boldsymbol{\Omega}^{\left( k \right)} \right)={N_{p}^{\mathbb{C}}\left( \boldsymbol{v}\left( t \right) | \mathbf{L}_{\boldsymbol{v\iota}}\boldsymbol{\iota}\left( t \right),{{\hat{\boldsymbol{\Theta}}}_{\boldsymbol{\xi\xi}}^{\left( k \right)}}^{-1} \right)N_{p}^{\mathbb{C}}\left( \boldsymbol{\iota}\left( t \right) | \mathbf{0},{{\hat{\boldsymbol{\Theta}}}_{\boldsymbol{\iota\iota}}^{\left( k \right)}}^{-1} \right)}/{p\left( \boldsymbol{v}\left( t \right) \right)}$

Analyzing the exponent (equation III-7) from the Bayesian expression (equation III-6) is enough to find the structure of the posterior distribution $p\left( \boldsymbol{\iota}\left( t \right) | \boldsymbol{v}\left( t \right)\mathbf{,}\boldsymbol{\Omega}^{\left( k \right)} \right)$.

$-\left( \boldsymbol{v}\left( t \right)-\mathbf{L}_{\boldsymbol{v\iota}}\boldsymbol{\iota}\left( t \right) \right)^{\dagger}{\hat{\boldsymbol{\Theta}}}_{\boldsymbol{\xi\xi}}^{\left( k \right)}\left( \boldsymbol{v}\left( t \right)-\mathbf{L}_{\boldsymbol{v\iota}}\boldsymbol{\iota}\left( t \right) \right)-\boldsymbol{\iota}^{\dagger}\left( t \right){\hat{\boldsymbol{\Theta}}}_{\boldsymbol{\iota\iota}}^{\left( k \right)}\boldsymbol{\iota}\left( t \right)$

Reorganizing terms in this exponent (equation III-7) we obtain the following expression (equation III-8), where $\mathbf{L}_{\boldsymbol{\iota v}}$ is the transpose for $\mathbf{L}_{\boldsymbol{v\iota}}$

$-\boldsymbol{\iota}^{\dagger}\left( t \right)\left( \mathbf{L}_{\boldsymbol{\iota v}}{\hat{\boldsymbol{\Theta}}}_{\boldsymbol{\xi\xi}}^{\left( k \right)}\mathbf{L}_{\boldsymbol{v\iota}}+{\hat{\boldsymbol{\Theta}}}_{\boldsymbol{\iota\iota}}^{\left( k \right)} \right)\boldsymbol{\iota}\left( t \right)+\boldsymbol{v}^{\dagger}\left( t \right){\hat{\boldsymbol{\Theta}}}_{\boldsymbol{\xi\xi}}^{\left( k \right)}\mathbf{L}_{\boldsymbol{v\iota}}\boldsymbol{\iota}\left( t \right)+\boldsymbol{\iota}^{\dagger}\left( t \right)\mathbf{L}_{\boldsymbol{\iota v}}{\hat{\boldsymbol{\Theta}}}_{\boldsymbol{\xi\xi}}^{\left( k \right)}\boldsymbol{v}\left( t \right)$

This expression (equation III-8) can then be expressed in terms of an auxiliary quantity in the EM iterations denominated source posterior precision-matrix ${\check{\boldsymbol{\Theta}}}_{\boldsymbol{\iota\iota}}^{\left( k \right)}$ (equation III-9).

$-\boldsymbol{\iota}^{\dagger}\left( t \right){\check{\boldsymbol{\Theta}}}_{\boldsymbol{\iota\iota}}^{\left( k \right)}\boldsymbol{\iota}\left( t \right)+\boldsymbol{v}^{\dagger}\left( t \right){\hat{\boldsymbol{\Theta}}}_{\boldsymbol{\xi\xi}}^{\left( k \right)}\mathbf{L}_{\boldsymbol{v\iota}}\boldsymbol{\iota}\left( t \right)+\boldsymbol{\iota}^{\dagger}\left( t \right)\mathbf{L}_{\boldsymbol{\iota v}}{\hat{\boldsymbol{\Theta}}}_{\boldsymbol{\xi\xi}}^{\left( k \right)}\boldsymbol{v}\left( t \right)$

Where this source posterior precision-matrix ${\check{\boldsymbol{\Theta}}}_{\boldsymbol{\iota\iota}}^{\left( k \right)}$ in (equation III-9), or source posterior covariance-matrix given by its inverse ${\check{\boldsymbol{\Sigma}}}_{\boldsymbol{\iota\iota}}^{\left( k \right)}={{\check{\boldsymbol{\Theta}}}_{\boldsymbol{\iota\iota}}^{\left( k \right)}}^{-1}$, is given by (equation III-10).

$\begin{matrix} {\check{\boldsymbol{\Theta}}}_{\boldsymbol{\iota\iota}}^{\left( k \right)}=\mathbf{L}_{\boldsymbol{\iota v}}{\hat{\boldsymbol{\Theta}}}_{\boldsymbol{\xi\xi}}^{\left( k \right)}\mathbf{L}_{\boldsymbol{v\iota}}+{\hat{\boldsymbol{\Theta}}}_{\boldsymbol{\iota\iota}}^{\left( k \right)} \\ {\check{\boldsymbol{\Sigma}}}_{\boldsymbol{\iota\iota}}^{\left( k \right)}\leftarrow\left( \mathbf{L}_{\boldsymbol{\iota v}}{\hat{\boldsymbol{\Theta}}}_{\boldsymbol{\xi\xi}}^{\left( k \right)}\mathbf{L}_{\boldsymbol{v\iota}}+{\hat{\boldsymbol{\Theta}}}_{\boldsymbol{\iota\iota}}^{\left( k \right)} \right)^{-1} \end{matrix}$

Introducing in the second and third term of the exponent (equation III-9) the product ${\check{\boldsymbol{\Theta}}}_{\boldsymbol{\iota\iota}}^{\left( k \right)}{\check{\boldsymbol{\Sigma}}}_{\boldsymbol{\iota\iota}}^{\left( k \right)}=\mathbf{I}_{q}$, of the source posterior precision-matrix and posterior covariance-matrix as given in (equation III-10) we obtain

$-\boldsymbol{\iota}^{\dagger}\left( t \right){\check{\boldsymbol{\Theta}}}_{\boldsymbol{\iota\iota}}^{\left( k \right)}\boldsymbol{\iota}\left( t \right)+\boldsymbol{v}^{\dagger}\left( t \right){\hat{\boldsymbol{\Theta}}}_{\boldsymbol{\xi\xi}}^{\left( k \right)}\mathbf{L}_{\boldsymbol{v\iota}}{\check{\boldsymbol{\Sigma}}}_{\boldsymbol{\iota\iota}}^{\left( k \right)}{\check{\boldsymbol{\Theta}}}_{\boldsymbol{\iota\iota}}^{\left( k \right)}\boldsymbol{\iota}\left( t \right)+\boldsymbol{\iota}^{\dagger}\left( t \right){\check{\boldsymbol{\Theta}}}_{\boldsymbol{\iota\iota}}^{\left( k \right)}{\check{\boldsymbol{\Sigma}}}_{\boldsymbol{\iota\iota}}^{\left( k \right)}\mathbf{L}_{\boldsymbol{\iota v}}{\hat{\boldsymbol{\Theta}}}_{\boldsymbol{\xi\xi}}^{\left( k \right)}\boldsymbol{v}\left( t \right)$

The second and third terms in the exponent (equation III-11) can be then expressed in terms of the estimates of the parameters in the EM iterations ${\hat{\boldsymbol{\iota}}}^{\left( k \right)}\left( t \right)$ and the source posterior precision-matrix ${\check{\boldsymbol{\Theta}}}_{\boldsymbol{\iota\iota}}^{\left( k \right)}$ (equation III-12).

$-\boldsymbol{\iota}^{\dagger}\left( t \right){\check{\boldsymbol{\Theta}}}_{\boldsymbol{\iota\iota}}^{\left( k \right)}\boldsymbol{\iota}\left( t \right)+{{\hat{\boldsymbol{\iota}}}^{\left( k \right)}}^{\dagger}\left( t \right){\check{\boldsymbol{\Theta}}}_{\boldsymbol{\iota\iota}}^{\left( k \right)}\boldsymbol{\iota}\left( t \right)+\boldsymbol{\iota}^{\dagger}\left( t \right){\check{\boldsymbol{\Theta}}}_{\boldsymbol{\iota\iota}}^{\left( k \right)}{\hat{\boldsymbol{\iota}}}^{\left( k \right)}\left( t \right)$

This parameter estimates ${\hat{\boldsymbol{\iota}}}^{\left( k \right)}\left( t \right)$ in (equation III-12) may be expressed locally linearly at EM iterations from the data $\boldsymbol{v}\left( t \right)$ in terms of another auxiliary quantity denominated source inverse-operator ${\check{\mathbf{T}}}_{\boldsymbol{\iota v}}^{\left( k \right)}$ for the lead field $\mathbf{L}_{\boldsymbol{v\iota}}$.

${\hat{\boldsymbol{\iota}}}^{\left( k \right)}\left( t \right)={\check{\mathbf{T}}}_{\boldsymbol{\iota v}}^{\left( k \right)}\boldsymbol{v}\left( t \right)$

Where the inverse-operator ${\check{\mathbf{T}}}_{\boldsymbol{\iota v}}^{\left( k \right)}$ in (equation III-13) is given in terms of the lead field $\mathbf{L}_{\boldsymbol{v\iota}}$, the source posterior covariance-matrix ${\check{\boldsymbol{\Sigma}}}_{\boldsymbol{\iota\iota}}^{\left( k \right)}$, and the residuals precision-matrix ${\hat{\boldsymbol{\Theta}}}_{\boldsymbol{\xi\xi}}^{\left( k \right)}$ as follows (equation III-14)

${\check{\mathbf{T}}}_{\boldsymbol{\iota v}}^{\left( k \right)}={\check{\boldsymbol{\Sigma}}}_{\boldsymbol{\iota\iota}}^{\left( k \right)}\mathbf{L}_{\boldsymbol{\iota v}}{\hat{\boldsymbol{\Theta}}}_{\boldsymbol{\xi\xi}}^{\left( k \right)}$

Completing the exponent in (equation III-12) with the term ${{\hat{\boldsymbol{\iota}}}^{\left( k \right)}}^{\dagger}\left( t \right){\check{\boldsymbol{\Theta}}}_{\boldsymbol{\iota\iota}}^{\left( k \right)}{\hat{\boldsymbol{\iota}}}^{\left( k \right)}\left( t \right)$ it is straightforward that the parameters posterior distribution can be defined as a circularly symmetric complex-valued Gaussian $N^{\mathbb{C}}$ (equation III-15) parametrized with the posterior covariance-matrix ${\check{\boldsymbol{\Sigma}}}_{\boldsymbol{\iota\iota}}^{\left( k \right)}$ from (equation III-10) and posterior mean ${\hat{\boldsymbol{\iota}}}^{\left( k \right)}\left( t \right)$ from (equation III-13).

$p\left( \boldsymbol{\iota}\left( t \right) | \boldsymbol{v}\left( t \right)\mathbf{,}{\hat{\boldsymbol{\Omega}}}^{\left( k \right)} \right)=N_{q}^{\mathbb{C}}\left( \boldsymbol{\iota}\left( t \right) | {\hat{\boldsymbol{\iota}}}^{\left( k \right)}\left( t \right),{\check{\boldsymbol{\Sigma}}}_{\boldsymbol{\iota\iota}}^{\left( k \right)} \right)$

The EM local approximation in HIGGS (equation III-1) for the intractable second-type likelihood $p^{\left( k \right)}\left( {\hat{\boldsymbol{\Sigma}}}_{\boldsymbol{vv}} | \boldsymbol{\Omega} \right)$ (equation III-3) is then expressed by means of the integral $Q\left( \boldsymbol{\Omega},{\hat{\boldsymbol{\Omega}}}^{\left( k \right)} \right)$ (equation III-4). This integral is interpreted as an expected $-log$ second-type likelihood that yields a local approximation to the second-type likelihood as a Gibb’s exponential form that must be combined with priors $p\left( \boldsymbol{\Omega} \right)$ for the second-type maximum a posteriori of the hyperparameters $\boldsymbol{\Omega}$. Expressing $Q\left( \boldsymbol{\Omega},{\hat{\boldsymbol{\Omega}}}^{\left( k \right)} \right)$ is much simpler by means of the additive terms $Q_{t}\left( \boldsymbol{\Omega},{\hat{\boldsymbol{\Omega}}}^{\left( k \right)} \right)=E\left( log\left( p\left( \boldsymbol{v}\left( t \right),\boldsymbol{\iota}\left( t \right) | \boldsymbol{\Omega} \right) \right) | \boldsymbol{v}\left( t \right)\mathbf{,}{\hat{\boldsymbol{\Omega}}}^{\left( k \right)} \right)$ (equation III-16), which are dependent on the data and parameters joint distribution $p\left( \boldsymbol{v}\left( t \right),\boldsymbol{\iota}\left( t \right) | \boldsymbol{\Omega} \right)$ and the parameters posterior distribution $p\left( \boldsymbol{\iota}\left( t \right) | \boldsymbol{v}\left( t \right)\mathbf{,}{\hat{\boldsymbol{\Omega}}}^{\left( k \right)} \right)$.

$\begin{matrix} Q\left( \boldsymbol{\Omega},{\hat{\boldsymbol{\Omega}}}^{\left( k \right)} \right)=\sum_{t\mathbb{\in T}} Q_{t}\left( \boldsymbol{\Omega},{\hat{\boldsymbol{\Omega}}}^{\left( k \right)} \right) \\ Q_{t}\left( \boldsymbol{\Omega},{\hat{\boldsymbol{\Omega}}}^{\left( k \right)} \right)=-E\left( log\left( p\left( \boldsymbol{v}\left( t \right),\boldsymbol{\iota}\left( t \right) | \boldsymbol{\Omega} \right) \right) | \boldsymbol{v}\left( t \right)\mathbf{,}{\hat{\boldsymbol{\Omega}}}^{\left( k \right)} \right) \end{matrix}$

This Bayesian formalism equivalent to the missing data problem ($\boldsymbol{\iota}\left( t \right)$), where the pair $\left\{ \boldsymbol{v}\left( t \right),\boldsymbol{\iota}\left( t \right) \right\}$ is denominated complete data and the joint distribution $p\left( \boldsymbol{v}\left( t \right),\boldsymbol{\iota}\left( t \right) | \boldsymbol{\Omega} \right)$ is denominated complete data likelihood. The complete data likelihood is factorized due to the Bayes theorem (equation III-17) into the observed data likelihood $p\left( \left. \boldsymbol{v}\left( t \right) \right|\boldsymbol{\iota}\left( t \right),\boldsymbol{\Omega} \right)$ and the missing data (parameters) likelihood $p\left( \left. \boldsymbol{\iota}\left( t \right) \right|\boldsymbol{\Omega} \right)$ that are according to the HIGGS model (equation III-1).

$p\left( \boldsymbol{v}\left( t \right),\boldsymbol{\iota}\left( t \right) | \boldsymbol{\Omega} \right)=p\left( \left. \boldsymbol{v}\left( t \right) \right|\boldsymbol{\iota}\left( t \right),\boldsymbol{\Omega} \right)p\left( \left. \boldsymbol{v}\left( t \right) \right|\boldsymbol{\iota}\left( t \right),\boldsymbol{\Omega} \right)=N_{p}^{\mathbb{C}}\left( \boldsymbol{v}\left( t \right) | \mathbf{L}_{\boldsymbol{v\iota}}\boldsymbol{\iota}\left( t \right),\boldsymbol{\Theta}_{\boldsymbol{\xi\xi}}^{-1} \right)N_{p}^{\mathbb{C}}\left( \boldsymbol{\iota}\left( t \right) | \mathbf{0},\boldsymbol{\Theta}_{\boldsymbol{\iota\iota}}^{-1} \right)$

The additive terms $Q_{t}\left( \boldsymbol{\Omega},{\hat{\boldsymbol{\Omega}}}^{\left( k \right)} \right)$ (equation III-16) due to the missing data posterior distribution $p\left( \boldsymbol{\iota}\left( t \right) | \boldsymbol{v}\left( t \right)\mathbf{,}{\hat{\boldsymbol{\Omega}}}^{\left( k \right)} \right)$ (equation III-15) and the complete data likelihood expression $p\left( \boldsymbol{v}\left( t \right),\boldsymbol{\iota}\left( t \right) | \boldsymbol{\Omega} \right)$ (equation III-17) and can be then expressed as follows (equation III-18).

$\begin{matrix} Q_{t}\left( \boldsymbol{\Omega},{\hat{\boldsymbol{\Omega}}}^{\left( k \right)} \right)=-E\left( log\left( p\left( \boldsymbol{v}\left( t \right),\boldsymbol{\iota}\left( t \right) | \boldsymbol{\Omega} \right) \right) | \boldsymbol{v}\left( t \right)\mathbf{,}{\hat{\boldsymbol{\Omega}}}^{\left( k \right)} \right) \\ E\left( log\left( p\left( \boldsymbol{v}\left( t \right),\boldsymbol{\iota}\left( t \right) | \boldsymbol{\Omega} \right) \right) | \boldsymbol{v}\left( t \right)\mathbf{,}{\hat{\boldsymbol{\Omega}}}^{\left( k \right)} \right)=\underset{\mathbb{C}^{q}}{\int}log\left( N_{p}^{\mathbb{C}}\left( \boldsymbol{v}\left( t \right) | \mathbf{L}_{\boldsymbol{v\iota}}\boldsymbol{\iota}\left( t \right),\boldsymbol{\Theta}_{\boldsymbol{\xi\xi}}^{-1} \right)N_{p}^{\mathbb{C}}\left( \boldsymbol{\iota}\left( t \right) | \mathbf{0},\boldsymbol{\Theta}_{\boldsymbol{\iota\iota}}^{-1} \right) \right) N_{q}^{\mathbb{C}}\left( \boldsymbol{\iota}\left( t \right) | {\hat{\boldsymbol{\iota}}}^{\left( k \right)}\left( t \right),{\check{\boldsymbol{\Sigma}}}_{\boldsymbol{\iota\iota}}^{\left( k \right)} \right)d\boldsymbol{\iota}\left( t \right) \end{matrix}$

Where the logarithm (equation III-18) of the factors $log\left( N_{p}^{\mathbb{C}}\left( \boldsymbol{v}\left( t \right) | \mathbf{L}_{\boldsymbol{v\iota}}\boldsymbol{\iota}\left( t \right),\boldsymbol{\Theta}_{\boldsymbol{\xi\xi}}^{-1} \right)N_{p}^{\mathbb{C}}\left( \boldsymbol{\iota}\left( t \right) | \mathbf{0},\boldsymbol{\Theta}_{\boldsymbol{\iota\iota}}^{-1} \right) \right)$ (equation III-17) can be expressed additively as (equation III-19).

$$log\left( N_{p}^{\mathbb{C}}\left( \boldsymbol{v}\left( t \right) | \mathbf{L}_{\boldsymbol{v\iota}}\boldsymbol{\iota}\left( t \right),\boldsymbol{\Theta}_{\boldsymbol{\xi\xi}}^{-1} \right)N_{p}^{\mathbb{C}}\left( \boldsymbol{\iota}\left( t \right) | \mathbf{0},\boldsymbol{\Theta}_{\boldsymbol{\iota\iota}}^{-1} \right) \right)=$$

$log\left| \boldsymbol{\Theta}_{\boldsymbol{\xi\xi}} \right|-\left( \boldsymbol{v}\left( t \right)-\mathbf{L}_{\boldsymbol{v\iota}}\boldsymbol{\iota}\left( t \right) \right)^{\dagger}\boldsymbol{\Theta}_{\boldsymbol{\xi\xi}}\left( \boldsymbol{v}\left( t \right)-\mathbf{L}_{\boldsymbol{v\iota}}\boldsymbol{\iota}\left( t \right) \right)+log\left| \boldsymbol{\Theta}_{\boldsymbol{\iota\iota}} \right|-\boldsymbol{\iota}^{\dagger}\left( t \right)\boldsymbol{\Theta}_{\boldsymbol{\iota\iota}}\boldsymbol{\iota}\left( t \right)$

Employing the trace properties to transform (equation III-19) we obtain (equation III-20) where an expansion of the second term in (III-19) is included from the second term to the fifth in (equation III-20).

$$log\left( N_{p}^{\mathbb{C}}\left( \boldsymbol{v}\left( t \right) | \mathbf{L}_{\boldsymbol{v\iota}}\boldsymbol{\iota}\left( t \right),\boldsymbol{\Theta}_{\boldsymbol{\xi\xi}}^{-1} \right)N_{p}^{\mathbb{C}}\left( \boldsymbol{\iota}\left( t \right) | \mathbf{0},\boldsymbol{\Theta}_{\boldsymbol{\iota\iota}}^{-1} \right) \right)=log\left| \boldsymbol{\Theta}_{\boldsymbol{\xi\xi}} \right|-tr\left( \boldsymbol{v}\left( t \right)\boldsymbol{v}^{\dagger}\left( t \right)\boldsymbol{\Theta}_{\boldsymbol{\xi\xi}} \right)+tr\left( \boldsymbol{v}\left( t \right)\boldsymbol{\iota}^{\dagger}\left( t \right)\mathbf{L}_{\boldsymbol{\iota v}}\boldsymbol{\Theta}_{\boldsymbol{\xi\xi}} \right)$$

$+tr\left( \mathbf{L}_{\boldsymbol{v\iota}}\boldsymbol{\iota}\left( t \right)\boldsymbol{v}\left( t \right)\boldsymbol{\Theta}_{\boldsymbol{\xi\xi}} \right)-tr\left( \mathbf{L}_{\boldsymbol{v\iota}}\boldsymbol{\iota}\left( t \right)\boldsymbol{\iota}^{\dagger}\left( t \right)\mathbf{L}_{\boldsymbol{\iota v}}\boldsymbol{\Theta}_{\boldsymbol{\xi\xi}} \right)+log\left| \boldsymbol{\Theta}_{\boldsymbol{\iota\iota}} \right|-tr\left( \boldsymbol{\iota}\left( t \right)\boldsymbol{\iota}^{\dagger}\left( t \right)\boldsymbol{\Theta}_{\boldsymbol{\iota\iota}} \right)$

From the expected $-log$ second-type likelihood (equation III-18) and the logarithm (equation III-20) we can then express $Q_{t}\left( \boldsymbol{\Omega},{\hat{\boldsymbol{\Omega}}}^{\left( k \right)} \right)$ as (equation III-21).

$Q_{t}\left( \boldsymbol{\Omega},{\hat{\boldsymbol{\Omega}}}^{\left( k \right)} \right)=-log\left| \boldsymbol{\Theta}_{\boldsymbol{\xi\xi}} \right|+tr\left( \boldsymbol{v}\left( t \right)\boldsymbol{v}^{\dagger}\left( t \right)\boldsymbol{\Theta}_{\boldsymbol{\xi\xi}} \right)-tr\left( \boldsymbol{v}\left( t \right)E\left( \boldsymbol{\iota}^{\dagger}\left( t \right) | \boldsymbol{v}\left( t \right)\mathbf{,}{\hat{\boldsymbol{\Omega}}}^{\left( k \right)} \right)\mathbf{L}_{\boldsymbol{\iota v}}\boldsymbol{\Theta}_{\boldsymbol{\xi\xi}} \right)-tr\left( \mathbf{L}_{\boldsymbol{v\iota}}E\left( \boldsymbol{\iota}\left( t \right) | \boldsymbol{v}\left( t \right)\mathbf{,}{\hat{\boldsymbol{\Omega}}}^{\left( k \right)} \right)\boldsymbol{v}\left( t \right)\boldsymbol{\Theta}_{\boldsymbol{\xi\xi}} \right)+tr\left( \mathbf{L}_{\boldsymbol{v\iota}}E\left( \boldsymbol{\iota}\left( t \right)\boldsymbol{\iota}^{\dagger}\left( t \right) | \boldsymbol{v}\left( t \right)\mathbf{,}{\hat{\boldsymbol{\Omega}}}^{\left( k \right)} \right)\mathbf{L}_{\boldsymbol{\iota v}}\boldsymbol{\Theta}_{\boldsymbol{\xi\xi}} \right)-log\left| \boldsymbol{\Theta}_{\boldsymbol{\iota\iota}} \right|+tr\left( E\left( \boldsymbol{\iota}\left( t \right)\boldsymbol{\iota}^{\dagger}\left( t \right) | \boldsymbol{v}\left( t \right)\mathbf{,}{\hat{\boldsymbol{\Omega}}}^{\left( k \right)} \right)\boldsymbol{\Theta}_{\boldsymbol{\iota\iota}} \right)$

Where it can be easily checked that for the expectation terms in (equation III-21) that following expressions hold (equation III-22).

$\begin{matrix} E\left( \boldsymbol{\iota}^{\dagger}\left( t \right) | \boldsymbol{v}\left( t \right)\mathbf{,}{\hat{\boldsymbol{\Omega}}}^{\left( k \right)} \right)={{\hat{\boldsymbol{\iota}}}^{\left( k \right)}}^{\dagger}\left( t \right) \\ E\left( \boldsymbol{\iota}\left( t \right) | \boldsymbol{v}\left( t \right)\mathbf{,}{\hat{\boldsymbol{\Omega}}}^{\left( k \right)} \right)={\hat{\boldsymbol{\iota}}}^{\left( k \right)}\left( t \right) \\ E\left( \boldsymbol{\iota}\left( t \right)\boldsymbol{\iota}^{\dagger}\left( t \right) | \boldsymbol{v}\left( t \right)\mathbf{,}{\hat{\boldsymbol{\Omega}}}^{\left( k \right)} \right)={\check{\boldsymbol{\Sigma}}}_{\boldsymbol{\iota\iota}}^{\left( k \right)}+{\hat{\boldsymbol{\iota}}}^{\left( k \right)}\left( t \right){{\hat{\boldsymbol{\iota}}}^{\left( k \right)}}^{\dagger}\left( t \right) \end{matrix}$

Then, plugging the expectation terms (equation III-22) into $Q_{t}\left( \boldsymbol{\Omega},{\hat{\boldsymbol{\Omega}}}^{\left( k \right)} \right)$ (equation III-21) we obtain (equation III-23).

$Q_{t}\left( \boldsymbol{\Omega},{\hat{\boldsymbol{\Omega}}}^{\left( k \right)} \right)=-log\left| \boldsymbol{\Theta}_{\boldsymbol{\xi\xi}} \right|+tr\left( \boldsymbol{v}\left( t \right)\boldsymbol{v}^{\dagger}\left( t \right)\boldsymbol{\Theta}_{\boldsymbol{\xi\xi}} \right)-tr\left( \boldsymbol{v}\left( t \right){{\hat{\boldsymbol{\iota}}}^{\left( k \right)}}^{\dagger}\left( t \right)\mathbf{L}_{\boldsymbol{\iota v}}\boldsymbol{\Theta}_{\boldsymbol{\xi\xi}} \right)-tr\left( \mathbf{L}_{\boldsymbol{v\iota}}{\hat{\boldsymbol{\iota}}}^{\left( k \right)}\left( t \right)\boldsymbol{v}\left( t \right)\boldsymbol{\Theta}_{\boldsymbol{\xi\xi}} \right)+tr\left( \mathbf{L}_{\boldsymbol{v\iota}}\left( {\check{\boldsymbol{\Sigma}}}_{\boldsymbol{\iota\iota}}^{\left( k \right)}+{\hat{\boldsymbol{\iota}}}^{\left( k \right)}\left( t \right){{\hat{\boldsymbol{\iota}}}^{\left( k \right)}}^{\dagger}\left( t \right) \right)\mathbf{L}_{\boldsymbol{\iota v}}\boldsymbol{\Theta}_{\boldsymbol{\xi\xi}} \right)-log\left| \boldsymbol{\Theta}_{\boldsymbol{\iota\iota}} \right|+tr\left( \left( {\check{\boldsymbol{\Sigma}}}_{\boldsymbol{\iota\iota}}^{\left( k \right)}+{\hat{\boldsymbol{\iota}}}^{\left( k \right)}\left( t \right){{\hat{\boldsymbol{\iota}}}^{\left( k \right)}}^{\dagger}\left( t \right) \right)\boldsymbol{\Theta}_{\boldsymbol{\iota\iota}} \right)$

Merging from the second term to the fifth in (equation III-23) we obtain (equation III-24).

$$Q_{t}\left( \boldsymbol{\Omega},{\hat{\boldsymbol{\Omega}}}^{\left( k \right)} \right)=-log\left| \boldsymbol{\Theta}_{\boldsymbol{\xi\xi}} \right|+tr\left( \left( \mathbf{L}_{\boldsymbol{v\iota}}{\check{\boldsymbol{\Sigma}}}_{\boldsymbol{\iota\iota}}^{\left( k \right)}\mathbf{L}_{\boldsymbol{\iota v}}+\boldsymbol{v}\left( t \right)\boldsymbol{v}^{\dagger}\left( t \right)-\boldsymbol{v}\left( t \right){{\hat{\boldsymbol{\iota}}}^{\left( k \right)}}^{\dagger}\left( t \right)\mathbf{L}_{\boldsymbol{\iota v}}-\mathbf{L}_{\boldsymbol{v\iota}}{\hat{\boldsymbol{\iota}}}^{\left( k \right)}\left( t \right)\boldsymbol{v}\left( t \right)+\mathbf{L}_{\boldsymbol{v\iota}}{\hat{\boldsymbol{\iota}}}^{\left( k \right)}\left( t \right){{\hat{\boldsymbol{\iota}}}^{\left( k \right)}}^{\dagger}\left( t \right)\mathbf{L}_{\boldsymbol{\iota v}} \right)\boldsymbol{\Theta}_{\boldsymbol{\xi\xi}} \right)$$

$-log\left| \boldsymbol{\Theta}_{\boldsymbol{\iota\iota}} \right|+tr\left( \left( {\check{\boldsymbol{\Sigma}}}_{\boldsymbol{\iota\iota}}^{\left( k \right)}+{\hat{\boldsymbol{\iota}}}^{\left( k \right)}\left( t \right){{\hat{\boldsymbol{\iota}}}^{\left( k \right)}}^{\dagger}\left( t \right) \right)\boldsymbol{\Theta}_{\boldsymbol{\iota\iota}} \right)$

Then (equation III-24) may be expressed in terms of residual posterior covariance-matrix ${\check{\boldsymbol{\Sigma}}}_{\boldsymbol{\xi\xi}}^{\left( k \right)}=\mathbf{L}_{\boldsymbol{v\iota}}{\check{\boldsymbol{\Sigma}}}_{\boldsymbol{\iota\iota}}^{\left( k \right)}\mathbf{L}_{\boldsymbol{\iota v}}$ (a projection by the Lead Field $\mathbf{L}_{\boldsymbol{v\iota}}$ of the posterior covariance-matrix for source cortical oscillations${\check{\boldsymbol{\Sigma}}}_{\boldsymbol{\iota\iota}}^{\left( k \right)}$) and the residuals estimates ${\hat{\boldsymbol{\xi}}}^{\left( k \right)}\left( t \right)\boldsymbol{=v}\left( t \right)-\mathbf{L}_{\boldsymbol{v\iota}}{\hat{\boldsymbol{\iota}}}^{\left( k \right)}\left( t \right)$ (equation III-25) compact and locally linear relations at EM iterations.

$$Q_{t}\left( \boldsymbol{\Omega},{\hat{\boldsymbol{\Omega}}}^{\left( k \right)} \right)=-log\left| \boldsymbol{\Theta}_{\boldsymbol{\xi\xi}} \right|+tr\left( \left( {\check{\boldsymbol{\Sigma}}}_{\boldsymbol{\xi\xi}}^{\left( k \right)}+{\hat{\boldsymbol{\xi}}}^{\left( k \right)}\left( t \right){{\hat{\boldsymbol{\xi}}}^{\left( k \right)}}^{\dagger}\left( t \right) \right)\boldsymbol{\Theta}_{\boldsymbol{\xi\xi}} \right)$$

$-log\left| \boldsymbol{\Theta}_{\boldsymbol{\iota\iota}} \right|+tr\left( \left( {\check{\boldsymbol{\Sigma}}}_{\boldsymbol{\iota\iota}}^{\left( k \right)}+{\hat{\boldsymbol{\iota}}}^{\left( k \right)}\left( t \right){{\hat{\boldsymbol{\iota}}}^{\left( k \right)}}^{\dagger}\left( t \right) \right)\boldsymbol{\Theta}_{\boldsymbol{\iota\iota}} \right)$

Then $Q\left( \boldsymbol{\Omega},{\hat{\boldsymbol{\Omega}}}^{\left( k \right)} \right)$ (equation III-16) can be expressed from the additive terms $Q_{t}\left( \boldsymbol{\Omega},{\hat{\boldsymbol{\Omega}}}^{\left( k \right)} \right)$ (equation III-25) to obtain the expected $-log$ second-type likelihood in terms of the sampled covariances for the residuals ${\hat{\boldsymbol{\Sigma}}}_{\boldsymbol{\xi\xi}}^{\left( k \right)}$ and source cortical oscillations${\hat{\boldsymbol{\Sigma}}}_{\boldsymbol{\iota\iota}}^{\left( k \right)}$, given for $T$ samples or time instances ($t\mathbb{\in T}$) (equation III-26).

$Q\left( \boldsymbol{\Omega},{\hat{\boldsymbol{\Omega}}}^{\left( k \right)} \right)=-Tlog\left| \boldsymbol{\Theta}_{\boldsymbol{\xi\xi}} \right|+Ttr\left( \left( {\check{\boldsymbol{\Sigma}}}_{\boldsymbol{\xi\xi}}^{\left( k \right)}+{\hat{\boldsymbol{\Sigma}}}_{\boldsymbol{\xi\xi}}^{\left( k \right)} \right)\boldsymbol{\Theta}_{\boldsymbol{\xi\xi}} \right)-Tlog\left| \boldsymbol{\Theta}_{\boldsymbol{\iota\iota}} \right|+Ttr\left( \left( {\check{\boldsymbol{\Sigma}}}_{\boldsymbol{\iota\iota}}^{\left( k \right)}+{\hat{\boldsymbol{\Sigma}}}_{\boldsymbol{\iota\iota}}^{\left( k \right)} \right)\boldsymbol{\Theta}_{\boldsymbol{\iota\iota}} \right)$

Where in (equation III-26) the sampled covariance-matrices for the residuals ${\hat{\boldsymbol{\Sigma}}}_{\boldsymbol{\xi\xi}}^{\left( k \right)}$ and source cortical oscillations${\hat{\boldsymbol{\Sigma}}}_{\boldsymbol{\iota\iota}}^{\left( k \right)}$ are defined as (equation III-27).

$\begin{matrix} {\hat{\boldsymbol{\Sigma}}}_{\boldsymbol{\xi\xi}}^{\left( k \right)}=\frac{1}{T}\sum_{t\mathbb{\in T}} {\hat{\boldsymbol{\xi}}}^{\left( k \right)}\left( t \right){{\hat{\boldsymbol{\xi}}}^{\left( k \right)}\left( t \right)}^{\dagger} \\ {\hat{\boldsymbol{\Sigma}}}_{\boldsymbol{\iota\iota}}^{\left( k \right)}=\frac{1}{T}\sum_{t\mathbb{\in T}} {\hat{\boldsymbol{\iota}}}^{\left( k \right)}\left( t \right){{\hat{\boldsymbol{\iota}}}^{\left( k \right)}}^{\dagger}\left( t \right) \end{matrix}$

These sampled covariances (equation III-27) may be expressed locally linearly at EM iterations from the data sampled covariance-matrix ${\hat{\boldsymbol{\Sigma}}}_{\boldsymbol{vv}}$ in terms of the inverse-operators for the residuals ${\check{\mathbf{T}}}_{\boldsymbol{\xi v}}^{\left( k \right)}$ and source cortical oscillations${\check{\mathbf{T}}}_{\boldsymbol{\iota v}}^{\left( k \right)}$ (equation III-28).

$\begin{matrix} {\hat{\boldsymbol{\Sigma}}}_{\boldsymbol{\xi\xi}}^{\left( k \right)}={\check{\mathbf{T}}}_{\boldsymbol{\xi v}}^{\left( k \right)}{\hat{\boldsymbol{\Sigma}}}_{\boldsymbol{vv}}{{\check{\mathbf{T}}}_{\boldsymbol{\xi v}}^{\left( k \right)}}^{\dagger} \\ {\hat{\boldsymbol{\Sigma}}}_{\boldsymbol{\iota\iota}}^{\left( k \right)}={\check{\mathbf{T}}}_{\boldsymbol{\iota v}}^{\left( k \right)}{\hat{\boldsymbol{\Sigma}}}_{\boldsymbol{vv}}{{\check{\mathbf{T}}}_{\boldsymbol{\iota v}}^{\left( k \right)}}^{\dagger} \end{matrix}$

This compact expression (equation III-28) is due to the relations of the residuals ${\hat{\boldsymbol{\xi}}}^{\left( k \right)}\left( t \right)$ and source cortical oscillations${\hat{\boldsymbol{\iota}}}^{\left( k \right)}\left( t \right)$ with the data $\boldsymbol{v}\left( t \right)$ (equation III-29).

$\begin{matrix} {\hat{\boldsymbol{\xi}}}^{\left( k \right)}\left( t \right)={\check{\mathbf{T}}}_{\boldsymbol{\xi v}}^{\left( k \right)}\boldsymbol{v}\left( t \right) \\ {\hat{\boldsymbol{\iota}}}^{\left( k \right)}\left( t \right)={\check{\mathbf{T}}}_{\boldsymbol{\iota v}}^{\left( k \right)}\boldsymbol{v}\left( t \right) \end{matrix}$

The inverse-operator ${\check{\mathbf{T}}}_{\boldsymbol{\xi v}}^{\left( k \right)}$ and relations for the residuals in (equation III-28) and (equation III-29) are given the residuals definition (equation III-30).

${\hat{\boldsymbol{\xi}}}^{\left( k \right)}\left( t \right)\boldsymbol{=v}\left( t \right)-\mathbf{L}_{\boldsymbol{v\iota}}{\hat{\boldsymbol{\iota}}}^{\left( k \right)}\left( t \right)$

This definition (equation III-30) may be reformulated due to the relation ${\hat{\boldsymbol{\iota}}}^{\left( k \right)}\left( t \right)={\check{\mathbf{T}}}_{\boldsymbol{\iota v}}^{\left( k \right)}\boldsymbol{v}\left( t \right)$ (equation III-13) that yields the following expression for ${\check{\mathbf{T}}}_{\boldsymbol{\xi v}}^{\left( k \right)}$ (equation III-31).

${\check{\mathbf{T}}}_{\boldsymbol{\xi v}}^{\left( k \right)}=\mathbf{I}_{p}-\mathbf{L}_{\boldsymbol{v\iota}}{\check{\mathbf{T}}}_{\boldsymbol{\iota v}}^{\left( k \right)}$

The expected $-log$ second-type likelihood $Q\left( \boldsymbol{\Omega},{\hat{\boldsymbol{\Omega}}}^{\left( k \right)} \right)$ (equation III-26) may be represented in bimodal compact form (equation III-32) through auxiliary quantities denominated effective covariance-matrices for residuals ${\check{\boldsymbol{\Psi}}}_{\boldsymbol{\xi\xi}}^{\left( k \right)}$ and source cortical oscillations${\check{\boldsymbol{\Psi}}}_{\boldsymbol{\iota\iota}}^{\left( k \right)}$.

$Q\left( \boldsymbol{\Omega},{\hat{\boldsymbol{\Omega}}}^{\left( k \right)} \right)=-Tlog\left| \boldsymbol{\Theta}_{\boldsymbol{\xi\xi}} \right|+Ttr\left( {\check{\boldsymbol{\Psi}}}_{\boldsymbol{\xi\xi}}^{\left( k \right)}\boldsymbol{\Theta}_{\boldsymbol{\xi\xi}} \right)-Tlog\left| \boldsymbol{\Theta}_{\boldsymbol{\iota\iota}} \right|+Ttr\left( {\check{\boldsymbol{\Psi}}}_{\boldsymbol{\iota\iota}}^{\left( k \right)}\boldsymbol{\Theta}_{\boldsymbol{\iota\iota}} \right)$

Where the effective covariance-matrix for source cortical oscillations${\check{\boldsymbol{\Psi}}}_{\boldsymbol{\iota\iota}}^{\left( k \right)}$ (equation III-33) is given by the additive combination of the posterior ${\check{\boldsymbol{\Sigma}}}_{\boldsymbol{\iota\iota}}^{\left( k \right)}$ and sampled ${\hat{\boldsymbol{\Sigma}}}_{\boldsymbol{\iota\iota}}^{\left( k \right)}$ covariance-matrices for source activity. The effective covariance-matrix for residuals ${\check{\boldsymbol{\Psi}}}_{\boldsymbol{\xi\xi}}^{\left( k \right)}$ (equation III-34) is given by the additive combination of the posterior ${\check{\boldsymbol{\Sigma}}}_{\boldsymbol{\xi\xi}}^{\left( k \right)}$ and sampled ${\hat{\boldsymbol{\Sigma}}}_{\boldsymbol{\xi\xi}}^{\left( k \right)}$ covariance-matrices for the residuals.

${\check{\boldsymbol{\Psi}}}_{\boldsymbol{\iota\iota}}^{\left( k \right)}={\check{\boldsymbol{\Sigma}}}_{\boldsymbol{\iota\iota}}^{\left( k \right)}+{\hat{\boldsymbol{\Sigma}}}_{\boldsymbol{\iota\iota}}^{\left( k \right)}$

${\check{\boldsymbol{\Psi}}}_{\boldsymbol{\xi\xi}}^{\left( k \right)}={\check{\boldsymbol{\Sigma}}}_{\boldsymbol{\xi\xi}}^{\left( k \right)}+{\hat{\boldsymbol{\Sigma}}}_{\boldsymbol{\xi\xi}}^{\left( k \right)}$

The expression $p^{\left( k \right)}\left( {\hat{\boldsymbol{\Sigma}}}_{\boldsymbol{vv}} | \boldsymbol{\Omega} \right)=exp\left( \left. Q\left( \boldsymbol{\Omega},\boldsymbol{\Omega}^{\left( k \right)} \right) \right|1 \right)$ (equation III-35) based on $Q\left( \boldsymbol{\Omega},{\hat{\boldsymbol{\Omega}}}^{\left( k \right)} \right)$ (equation III-32) yields the factor, where the effective covariance-matrix for source cortical oscillations${\check{\boldsymbol{\Psi}}}_{\boldsymbol{\iota\iota}}^{\left( k \right)}$ (equation III-33) and residuals ${\check{\boldsymbol{\Psi}}}_{\boldsymbol{\xi\xi}}^{\left( k \right)}$ (equation III-34) are a function of ${\hat{\boldsymbol{\Sigma}}}_{\boldsymbol{vv}}$ through (equation III-28) the sampled covariance-matrices for source cortical oscillations${\hat{\boldsymbol{\Sigma}}}_{\boldsymbol{\iota\iota}}^{\left( k \right)}$ and residuals ${\hat{\boldsymbol{\Sigma}}}_{\boldsymbol{\xi\xi}}^{\left( k \right)}$.

$$p^{\left( k \right)}\left( {\hat{\boldsymbol{\Sigma}}}_{\boldsymbol{vv}} | \boldsymbol{\Omega} \right)=exp\left( \left. -Tlog\left| \boldsymbol{\Theta}_{\boldsymbol{\xi\xi}} \right|+Ttr\left( {\check{\boldsymbol{\Psi}}}_{\boldsymbol{\xi\xi}}^{\left( k \right)}\boldsymbol{\Theta}_{\boldsymbol{\xi\xi}} \right) \right|1 \right)exp\left( \left. -Tlog\left| \boldsymbol{\Theta}_{\boldsymbol{\iota\iota}} \right|+Ttr\left( {\check{\boldsymbol{\Psi}}}_{\boldsymbol{\iota\iota}}^{\left( k \right)}\boldsymbol{\Theta}_{\boldsymbol{\iota\iota}} \right) \right|1 \right)$$

$\begin{matrix} p^{\left( k \right)}\left( {\hat{\boldsymbol{\Sigma}}}_{\boldsymbol{vv}} | \boldsymbol{\Omega} \right)=p^{\left( k \right)}\left( \left. {\hat{\boldsymbol{\Sigma}}}_{\boldsymbol{vv}} \right|\boldsymbol{\Theta}_{\boldsymbol{\iota\iota}} \right)p^{\left( k \right)}\left( \left. {\hat{\boldsymbol{\Sigma}}}_{\boldsymbol{vv}} \right|\boldsymbol{\Theta}_{\boldsymbol{\xi\xi}} \right) \\ p^{\left( k \right)}\left( \left. {\hat{\boldsymbol{\Sigma}}}_{\boldsymbol{vv}} \right|\boldsymbol{\Theta}_{\boldsymbol{\iota\iota}} \right)\boldsymbol{=}W_{q}^{\mathbb{C}}\left( {\check{\boldsymbol{\Psi}}}_{\boldsymbol{\iota\iota}}^{\left( k \right)} | T^{-1}\boldsymbol{\Theta}_{\boldsymbol{\iota\iota}}^{-1},T \right) \\ p^{\left( k \right)}\left( \left. {\hat{\boldsymbol{\Sigma}}}_{\boldsymbol{vv}} \right|\boldsymbol{\Theta}_{\boldsymbol{\xi\xi}} \right)\boldsymbol{=}W_{p}^{\mathbb{C}}\left( {\check{\boldsymbol{\Psi}}}_{\boldsymbol{\xi\xi}}^{\left( k \right)} | T^{-1}\boldsymbol{\Theta}_{\boldsymbol{\xi\xi}}^{-1},T \right) \end{matrix}$

## HIGGS second-type maximum a posteriori and priors (Corollary to Lemma 1)

The second-type maximum a posteriori distribution $p^{\left( k \right)}\left( \boldsymbol{\Omega} | {\hat{\boldsymbol{\Sigma}}}_{\boldsymbol{vv}} \right)$, locally approximated at EM iterations, of the Hidden Gaussian Graphical (HIGGS) model hyperparameters, Lemma 1 (equation III-1), is thus expressed analytically by the Bayes theorem (equation IV-1). This Bayes theorem combines second-type likelihood iterated approximation $p^{\left( k \right)}\left( {\hat{\boldsymbol{\Sigma}}}_{\boldsymbol{vv}} | \boldsymbol{\Omega} \right)$, as a function of the observed cross-spectrum ${\hat{\boldsymbol{\Sigma}}}_{\boldsymbol{vv}}$, and hyperparameters prior $p\left( \boldsymbol{\Omega} \right)$.

$p^{\left( k \right)}\left( \boldsymbol{\Omega} | {\hat{\boldsymbol{\Sigma}}}_{\boldsymbol{vv}} \right)=\frac{p^{\left( k \right)}\left( {\hat{\boldsymbol{\Sigma}}}_{\boldsymbol{vv}} | \boldsymbol{\Omega} \right)p\left( \boldsymbol{\Omega} \right)}{p\left( {\hat{\boldsymbol{\Sigma}}}_{\boldsymbol{vv}} \right)}$

As expressed by the expected $-log$ second-type likelihood from the EM integral operation $Q\left( \boldsymbol{\Omega},{\hat{\boldsymbol{\Omega}}}^{\left( k \right)} \right)$ the expected second-type likelihood above $p^{\left( k \right)}\left( {\hat{\boldsymbol{\Sigma}}}_{\boldsymbol{vv}} | \boldsymbol{\Omega} \right)$ is given by the Gibbs form (equation IV-2).

$p^{\left( k \right)}\left( {\hat{\boldsymbol{\Sigma}}}_{\boldsymbol{vv}} | \boldsymbol{\Omega} \right)=exp\left( \left. Q\left( \boldsymbol{\Omega},\boldsymbol{\Omega}^{\left( k \right)} \right) \right|1 \right)$

The second-type maximum a posteriori estimator for hyperparameters ${\hat{\boldsymbol{\Omega}}}^{\left( k+1 \right)}$ may then be obtained maximizing the posterior distribution $p^{\left( k \right)}\left( \boldsymbol{\Omega} | {\hat{\boldsymbol{\Sigma}}}_{\boldsymbol{vv}} \right)$ that is given by the proportion (equation IV-3) of $p^{\left( k \right)}\left( {\hat{\boldsymbol{\Sigma}}}_{\boldsymbol{vv}} | \boldsymbol{\Omega} \right)$ as a function of $Q\left( \boldsymbol{\Omega},{\hat{\boldsymbol{\Omega}}}^{\left( k \right)} \right)$ and $p\left( \boldsymbol{\Omega} \right)$.

$\begin{matrix} {\hat{\boldsymbol{\Omega}}}^{\left( k+1 \right)}\boldsymbol{=}{argmax}_{\boldsymbol{\Omega}}\left( p^{\left( k \right)}\left( \boldsymbol{\Omega} | {\hat{\boldsymbol{\Sigma}}}_{\boldsymbol{vv}} \right) \right) \\ p^{\left( k \right)}\left( \boldsymbol{\Omega} | {\hat{\boldsymbol{\Sigma}}}_{\boldsymbol{vv}} \right)\propto exp\left( \left. Q\left( \boldsymbol{\Omega},{\hat{\boldsymbol{\Omega}}}^{\left( k \right)} \right) \right|1 \right)p\left( \boldsymbol{\Omega} \right) \end{matrix}$

Or equivalently, this second-type maximum a posteriori estimator for hyperparameters ${\hat{\boldsymbol{\Omega}}}^{\left( k+1 \right)}$ (equation IV-3), may then be obtained minimizing the target function $\mathcal{L}^{\left( k \right)}\left( \boldsymbol{\Omega} \right)$ (equation IV-4).

$\begin{matrix} {\hat{\boldsymbol{\Omega}}}^{\left( k+1 \right)}\boldsymbol{=}{argmin}_{\boldsymbol{\Omega}}\left( \mathcal{L}^{\left( k \right)}\left( \boldsymbol{\Omega} \right) \right) \\ \mathcal{L}^{\left( k \right)}\left( \boldsymbol{\Omega}\left( f \right) \right)=Q\left( \boldsymbol{\Omega},{\hat{\boldsymbol{\Omega}}}^{\left( k \right)} \right)-\log p\left( \boldsymbol{\Omega} \right) \end{matrix}$

Because of the bimodal $-log$ Wishart form of $Q\left( \boldsymbol{\Omega},{\hat{\boldsymbol{\Omega}}}^{\left( k \right)} \right)$, a consequence of Lemma 1 (equation III-3), that implies the separability $\mathcal{L}^{\left( k \right)}\left( \boldsymbol{\Omega} \right)=\mathcal{L}^{\left( k \right)}\left( \boldsymbol{\Theta}_{\boldsymbol{\iota\iota}} \right)+\mathcal{L}^{\left( k \right)}\left( \boldsymbol{\Theta}_{\boldsymbol{\xi\xi}} \right)$ for the precision-matrices $\boldsymbol{\Omega}=\left\{ \boldsymbol{\Theta}_{\boldsymbol{\iota\iota}},\boldsymbol{\Theta}_{\boldsymbol{\xi\xi}} \right\}$ in (equation IV-4) and their a priori independency $p\left( \boldsymbol{\Omega} \right)=p\left( \boldsymbol{\Theta}_{\boldsymbol{\iota\iota}} \right)p\left( \boldsymbol{\Theta}_{\boldsymbol{\xi\xi}} \right)$, the second-type maximum a posteriori estimators ${\hat{\boldsymbol{\Omega}}}^{\left( k+1 \right)}=\left\{ {\hat{\boldsymbol{\Theta}}}_{\boldsymbol{\iota\iota}}^{\left( k+1 \right)},{\hat{\boldsymbol{\Theta}}}_{\boldsymbol{\xi\xi}}^{\left( k+1 \right)} \right\}$ are given by (equation IV-5) for ${\hat{\boldsymbol{\Theta}}}_{\boldsymbol{\iota\iota}}^{\left( k+1 \right)}$ and (equation IV-6) for ${\hat{\boldsymbol{\Theta}}}_{\boldsymbol{\xi\xi}}^{\left( k+1 \right)}$.

$\begin{matrix} {\hat{\boldsymbol{\Theta}}}_{\boldsymbol{\iota\iota}}^{\left( k+1 \right)}\boldsymbol{=}{argmin}_{\boldsymbol{\Theta}_{\boldsymbol{\iota\iota}}}\left( \mathcal{L}^{\left( k \right)}\left( \boldsymbol{\Theta}_{\boldsymbol{\iota\iota}} \right) \right) \\ \mathcal{L}^{\left( k \right)}\left( \boldsymbol{\Theta}_{\boldsymbol{\iota\iota}} \right)=-Tlog\left| \boldsymbol{\Theta}_{\boldsymbol{\iota\iota}} \right|+Ttr\left( {\check{\boldsymbol{\Psi}}}_{\boldsymbol{\iota\iota}}^{\left( k \right)}\boldsymbol{\Theta}_{\boldsymbol{\iota\iota}} \right)-\log p\left( \boldsymbol{\Theta}_{\boldsymbol{\iota\iota}} \right) \end{matrix}$

$\begin{matrix} {\hat{\boldsymbol{\Theta}}}_{\boldsymbol{\xi\xi}}^{\left( k+1 \right)}\boldsymbol{=}{argmin}_{\boldsymbol{\Theta}_{\boldsymbol{\xi\xi}}}\left( \mathcal{L}^{\left( k \right)}\left( \boldsymbol{\Theta}_{\boldsymbol{\xi\xi}} \right) \right) \\ \mathcal{L}^{\left( k \right)}\left( \boldsymbol{\Theta}_{\boldsymbol{\xi\xi}} \right)=-Tlog\left| \boldsymbol{\Theta}_{\boldsymbol{\xi\xi}} \right|+Ttr\left( {\check{\boldsymbol{\Psi}}}_{\boldsymbol{\xi\xi}}^{\left( k \right)}\boldsymbol{\Theta}_{\boldsymbol{\xi\xi}} \right)-\log p\left( \boldsymbol{\Theta}_{\boldsymbol{\xi\xi}} \right) \end{matrix}$

The final expression of the target functions above for the sources $\mathcal{L}^{\left( k \right)}\left( \boldsymbol{\Theta}_{\boldsymbol{\iota\iota}} \right)$ (equation IV-5), and residuals $\mathcal{L}^{\left( k \right)}\left( \boldsymbol{\Theta}_{\boldsymbol{\xi\xi}} \right)$, (equation IV-6), defining the solutions of iterated Gaussian Graphical Spectral (GGS) models, is due to the priors upon the precision-matrices $\boldsymbol{\Theta}_{\boldsymbol{\iota\iota}}$ (equation IV-7) and $\boldsymbol{\Theta}_{\boldsymbol{\xi\xi}}$ (equation IV-8).

$p\left( \boldsymbol{\Theta}_{\boldsymbol{\iota\iota}} \right)=\exp\left( \Pi\left( \mathbf{A}_{\boldsymbol{\iota\iota}}\boldsymbol{\odot}\boldsymbol{\Theta}_{\boldsymbol{\iota\iota}} \right) | \alpha_{\boldsymbol{\iota}}T \right)$

$p\left( \theta_{\boldsymbol{\xi}}^{2} \right)=\exp\left( \theta_{\boldsymbol{\xi}}^{2} | \alpha_{\boldsymbol{\xi}}pT \right)$

Where the prior structure of the GGS precision-matrices is represented wuith the ad-hoc $\mathbf{A}_{\boldsymbol{\iota\iota}}$ for the sources (equation IV-6), and $\mathbf{A}_{\boldsymbol{\xi\xi}}$ for the residuals (equation IV-7). The scale (regularization) parameters control the level of penalization exerted by these priors, given by $\alpha_{\boldsymbol{\iota}}$ that impose $\Pi$ the L1 or L2 norm upon the multivariate $\boldsymbol{\Theta}_{\boldsymbol{\iota\iota}}$, and $\alpha_{\boldsymbol{\xi}}$ that may be interpreted as the residuals inferior limit represented by the scalar precison $\theta_{\boldsymbol{\xi}}^{2}$ due to a reparameterization of the residuals precision-matrix $\boldsymbol{\Theta}_{\boldsymbol{\xi\xi}}=\theta_{\boldsymbol{\xi}}^{2}\mathbf{A}_{\boldsymbol{\xi\xi}}$. The estimators for the second-type maximum a posteriori are then expressed as (equation IV-9), for ${\hat{\boldsymbol{\Theta}}}_{\boldsymbol{\iota\iota}}^{\left( k+1 \right)}$ based on the source target function (equation IV-5) and prior (equation IV-7), and (equation IV-10), for ${\hat{\boldsymbol{\Theta}}}_{\boldsymbol{\xi\xi}}^{\left( k+1 \right)}$ based on the residual target function (equation IV-6) and prior (equation IV-8).

${\hat{\boldsymbol{\Theta}}}_{\boldsymbol{\iota\iota}}^{\left( k+1 \right)}={argmin}_{\boldsymbol{\Theta}_{\boldsymbol{\iota\iota}}}\left\{ -log\left| \boldsymbol{\Theta}_{\boldsymbol{\iota\iota}} \right|+tr\left( {\check{\boldsymbol{\Psi}}}_{\boldsymbol{\iota\iota}}^{\left( k \right)}\boldsymbol{\Theta}_{\boldsymbol{\iota\iota}} \right)+\alpha_{\boldsymbol{\iota}}\Pi\left( \mathbf{A}_{\boldsymbol{\iota\iota}}\boldsymbol{\odot}\boldsymbol{\Theta}_{\boldsymbol{\iota\iota}} \right) \right\}$

${\hat{\theta}_{\boldsymbol{\xi}}^{2}}^{\left( k+1 \right)}={argmin}_{\theta_{\boldsymbol{\xi}}^{2}}\left\{ -plog\left( \theta_{\boldsymbol{\xi}}^{2} \right)+\theta_{\boldsymbol{\xi}}^{2}tr\left( {\check{\boldsymbol{\Psi}}}_{\boldsymbol{\xi\xi}}^{\left( k \right)}\mathbf{A}_{\boldsymbol{\xi\xi}} \right)\boldsymbol{+}\alpha_{\boldsymbol{\xi}}p\theta_{\boldsymbol{\xi}}^{2} \right\}$

Note that the penalty$\Pi$ defines the particular flavor of the second-type maximum a posteriori estimates for the precision-matrix ${\hat{\boldsymbol{\Theta}}}_{\boldsymbol{\iota\iota}}^{\left( k+1 \right)}$ of the hermitian graphical model of the sources placing in the target function (equation IV-9): “hgNaive” with $\Pi=0$ (equation IV-11), “Ridge” with $\Pi=\left\| \cdot\right\|_{2}^{2}$ (equation IV-12), and “hgLASSO” with $\Pi=\left\| \cdot\right\|_{1}$ (equation IV-13).

${\hat{\boldsymbol{\Theta}}}_{\boldsymbol{\iota\iota}}^{\left( k+1 \right)}={argmin}_{\boldsymbol{\Theta}_{\boldsymbol{\iota\iota}}}\left\{ -log\left| \boldsymbol{\Theta}_{\boldsymbol{\iota\iota}} \right|+tr\left( {\check{\boldsymbol{\Psi}}}_{\boldsymbol{\iota\iota}}^{\left( k \right)}\boldsymbol{\Theta}_{\boldsymbol{\iota\iota}} \right) \right\}$

${\hat{\boldsymbol{\Theta}}}_{\boldsymbol{\iota\iota}}^{\left( k+1 \right)}={argmin}_{\boldsymbol{\Theta}_{\boldsymbol{\iota\iota}}}\left\{ -log\left| \boldsymbol{\Theta}_{\boldsymbol{\iota\iota}} \right|+tr\left( {\check{\boldsymbol{\Psi}}}_{\boldsymbol{\iota\iota}}^{\left( k \right)}\boldsymbol{\Theta}_{\boldsymbol{\iota\iota}} \right)+\alpha_{\boldsymbol{\iota}}\left\| \mathbf{A}_{\boldsymbol{\iota\iota}}\boldsymbol{\odot}\boldsymbol{\Theta}_{\boldsymbol{\iota\iota}} \right\|_{2}^{2} \right\}$

${\hat{\boldsymbol{\Theta}}}_{\boldsymbol{\iota\iota}}^{\left( k+1 \right)}={argmin}_{\boldsymbol{\Theta}_{\boldsymbol{\iota\iota}}}\left\{ -log\left| \boldsymbol{\Theta}_{\boldsymbol{\iota\iota}} \right|+tr\left( {\check{\boldsymbol{\Psi}}}_{\boldsymbol{\iota\iota}}^{\left( k \right)}\boldsymbol{\Theta}_{\boldsymbol{\iota\iota}} \right)+\alpha_{\boldsymbol{\iota}}\left\| \mathbf{A}_{\boldsymbol{\iota\iota}}\boldsymbol{\odot}\boldsymbol{\Theta}_{\boldsymbol{\iota\iota}} \right\|_{1} \right\}$

The second-type maximum a posteriori estimates for the scalar precision ${\hat{\theta}_{\boldsymbol{\xi}}^{2}}^{\left( k+1 \right)}$, a reparameterization $\boldsymbol{\Theta}_{\boldsymbol{\xi\xi}}=\theta_{\boldsymbol{\xi}}^{2}\mathbf{A}_{\boldsymbol{\xi\xi}}$ for the hermitian graphical model of the residuals, can be obtained in terms of the zero (equation IV-14) due to the direct differentiation of the target function (equation IV-10).

$\begin{matrix} {\hat{\theta}_{\boldsymbol{\xi}}^{2}}^{\left( k+1 \right)}=1/\left( {tr\left( {\check{\boldsymbol{\Psi}}}_{\boldsymbol{\xi\xi}}^{\left( k \right)}\left( f \right)\mathbf{A}_{\boldsymbol{\xi\xi}} \right)}/p+\alpha_{\boldsymbol{\xi}} \right) \\ {\hat{\theta}_{\boldsymbol{\xi}}^{2}}^{\left( k+1 \right)}={zero}_{\theta_{\boldsymbol{\xi}}^{2}}\left\{ -p\theta_{\boldsymbol{\xi}}^{-2}+tr\left( {\check{\boldsymbol{\Psi}}}_{\boldsymbol{\xi\xi}}^{\left( k \right)}\mathbf{A}_{\boldsymbol{\xi\xi}} \right)\boldsymbol{+}\alpha_{\boldsymbol{\xi}}p \right\} \end{matrix}$

## Complex-valued Andrews and Mallows Lemma: Local Quadratic Approximation (LQA) of the hermitian graphical LASSO (hgLASSO) prior (Lemma 2)

***Lemma 2 (corollary of Andrews and Mallows for the hermitian graphical LASSO):*** *The measurable space of the random matrix* $\boldsymbol{\Theta}$ *with Gibbs probability density function* $p\left( \boldsymbol{\Theta} \right)=\exp\left( \Pi\left( \boldsymbol{A\odot\Theta} \right) | \alpha T \right)$*, with penalization function of the hermitian graphical LASSO model (*$\Pi=\left\| \cdot\right\|_{1}$*), admits a hierarchical (conditional) representation in the product of measurable spaces for the random variables: 1) a conditional expectation* $\left. \boldsymbol{\Theta} \right|\boldsymbol{\Gamma}$ *(equation V-1) with Gaussian improper probability density function (a density but not a probability), and 2)* $\boldsymbol{\Gamma}$ *(equation V-2) with Gamma probability density function.*

$p\left( \boldsymbol{\Theta} | \boldsymbol{\Gamma} \right)\propto\prod_{i,j=1}^{q} N_{1}\left( \left| \boldsymbol{\Theta}\left( i,j \right) \right| | 0,{\boldsymbol{\Gamma}^{2}\left( i,j \right)}/T \right)$

$p\left( \boldsymbol{\Theta} | \boldsymbol{\Gamma} \right)\mathbf{=}\prod_{i,j}^{q} Ga\left( \boldsymbol{\Gamma}^{2}\left( i,j \right) | 1,{T\alpha^{2}\mathbf{A}^{2}\left( i,j \right)}/2 \right)$

***Proof of Lemma 2***

The hierarchical representation of the Gibbs prior with hermitian graphical LASSO exponent $\Pi\left( \boldsymbol{A\odot\Theta} \right)=\left\| \boldsymbol{A\odot\Theta} \right\|_{1}$, can be built on corollaries of the Andrews and Mallows Lemma [5], for the extension of real-valued Laplace probability density function to the real-valued or complex-valued matrix case, by considering improper density functions or simply more general measurable spaces. By the Andrews and Mallows Lemma in the real-valued Laplace, also for the real-valued or complex-valued case the integral representation holds (equation V-3).

$e^{-\alpha\left| z \right|}=\int N_{1}\left( \left| z \right| | 0,x \right)Ga\left( x | 1,{\alpha^{2}}/2 \right)dx$

The measurable space in which the variable $\left. z \right|x$ is given by the Gaussian improper probability density function $p\left( z | x \right)=N_{1}\left( \left| z \right| | 0,x \right)$, where its variance $x$ has Gamma probability density function $p\left( x \right)=Ga\left( x | 1,{\alpha^{2}}/2 \right)$ (equation V-3). Therefore, the measure in the product space of $\left. z \right|x$ and $\tau$ is has density represented as an improper product of Gaussian and Gamma probability density functions (equation V-4). We denominate this the generalization of Andrews and Mallows Lemma for real-valued or complex-valued Laplace probability density function.

$p\left( z,x \right)\propto N_{1}\left( \left| z \right| | 0,x \right)Ga\left( x | 1,{\alpha^{2}}/2 \right)$

The hermitian graphical LASSO Gibbs probability density function, that may be expressed for $\boldsymbol{\Theta}$ as (equation V-5), there is a priori independency of the precision-matrix elements $p\left( \boldsymbol{\Theta} \right)=\prod_{i,j=1}^{q} p\left( \boldsymbol{\Theta}\left( i,j \right) \right)$, and therefore $\boldsymbol{\Theta}\left( i,j \right)$ possesses a Gibbs probability density function as follows (equation V-6).

$p\left( \boldsymbol{\Theta} \right)=\exp\left( \left\| \boldsymbol{A\odot\Theta} \right\|_{1} | \alpha T \right)$

$p\left( \boldsymbol{\Theta}\left( i,j \right) \right)=\exp\left( \left| \mathbf{A}\left( i,j \right)\boldsymbol{\Theta}\left( i,j \right) \right| | \alpha T \right)$

If we apply the generalization of Andrews and Mallows Lemma for real-valued or complex-valued Laplace probability density function (equation V-4) to $\exp\left( \left| \mathbf{A}\left( i,j \right)\boldsymbol{\Theta}\left( i,j \right) \right| | \alpha T \right)$ in (equation V-6), by substituting $\alpha=\alpha T^{1/2}\mathbf{A}\left( i,j \right)$, $z=T^{1/2}\boldsymbol{\Theta}\left( i,j \right)$ and $x=\boldsymbol{\Gamma}^{2}\left( i,j \right)$ for the measure in the product space of the precision-matrix elements conditional expectation $\left. \boldsymbol{\Theta}\left( i,j \right) \right|\boldsymbol{\Gamma}\left( i,j \right)$ and variance matrix elements $\boldsymbol{\Gamma}\left( i,j \right)$ we obtain (equation V-7).

$p\left( \boldsymbol{\Theta}\left( i,j \right),\boldsymbol{\Gamma}\left( i,j \right) \right)\propto N_{1}^{T}\left( \left| \boldsymbol{\Theta}\left( i,j \right) \right| | 0,{\boldsymbol{\Gamma}^{2}\left( i,j \right)}/T \right){Ga}^{T}\left( \boldsymbol{\Gamma}^{2}\left( i,j \right) | 1,{T\alpha^{2}\mathbf{A}^{2}\left( i,j \right)}/2 \right)$

For $\exp\left( \left\| \boldsymbol{A\odot\Theta} \right\|_{1} | \alpha T \right)$ in (equation V-5) the measure in the product space of the precision-matrix conditional expectation $\left. \boldsymbol{\Theta} \right|\boldsymbol{\Gamma}$ and variances matrix $\boldsymbol{\Gamma}$ we obtain (equation V-8).

$p\left( \boldsymbol{\Theta},\boldsymbol{\Gamma} \right)=\prod_{i,j}^{q} N_{1}^{T}\left( \left| \boldsymbol{\Theta}\left( i,j \right) \right| | 0,{\boldsymbol{\Gamma}^{2}\left( i,j \right)}/T \right){Ga}^{T}\left( \boldsymbol{\Gamma}^{2}\left( i,j \right) | 1,T{\alpha^{2}\mathbf{A}^{2}\left( i,j \right)}/2 \right)$ $∎$

**Remark:** In Lemma 2 we stablish a statistical equivalence between the Gibbs probability density function with argument in the hermitian graphical LASSO prior and a hierarchical improper representation through a Gaussian probability density function of the conditional expectation $\left. \boldsymbol{\Theta} \right|\boldsymbol{\Gamma}$, and variances (weights) $\boldsymbol{\Gamma}$ with Gamma probability density function. Remarkably, this representation we are not using a probability density function in the strict mathematical sense but a measure density of a product of measurable spaces for $\left. \boldsymbol{\Theta} \right|\boldsymbol{\Gamma}$ and $\boldsymbol{\Gamma}$.

## Concavity of the first-type maximum a posteriori with hgLASSO LQA prior (Lemma 3)

***Lemma 3 (concavity of the Local Quadratic Approximation for the hermitian graphical LASSO prior):*** *The target function* $\mathcal{L}\left( \boldsymbol{\Theta},\boldsymbol{\Gamma} \right)$ *(*$-log$ *of the improper probability density function for the measurable product space* $\left. \boldsymbol{\Theta} \right|\boldsymbol{\Gamma}\times\boldsymbol{\Gamma}$*) may be represented as (equation VI-1) an it is strictly concave on the intercept of the region of positive definiteness of its arguments (equation VI-2) and the region comprehended by the set of inequalities (equation VI-3).*

$\mathcal{L}\left( \boldsymbol{\Theta,\Gamma} \right)=-T\log\left| \boldsymbol{\Theta} \right| +T tr\left( \boldsymbol{\Psi\Theta} \right)+\frac{T}{2}\left\| \boldsymbol{\Theta}⊘\boldsymbol{\Gamma} \right\|_{2}^{2}+\frac{1}{2}\sum_{i,j=1}^{q} \log\boldsymbol{\Gamma}^{2}\left( i,j \right)+\frac{T\alpha^{2}}{2}\sum_{i,j=1}^{q} \boldsymbol{\Gamma}^{2}\left( i,j \right)\mathbf{A}^{2}\left( i,j \right)$

$\left\{ \boldsymbol{\Theta}\boldsymbol{≽}0\boldsymbol{,}\boldsymbol{\Gamma}\boldsymbol{≽}0 \right\}$

$\left\{ 3T\left| \boldsymbol{\Theta}\left( i,j \right) \right|^{2}-\boldsymbol{\Gamma}^{2}\left( i,j \right)+T\alpha^{2}\mathbf{A}^{2}\left( i,j \right)\boldsymbol{\Gamma}^{4}\left( i,j \right)\geq0 \right\}_{i,j=1}^{q}$

*Then,* $\mathcal{L}\left( \boldsymbol{\Theta},\boldsymbol{\Gamma} \right)$ *has a minimum within these regions defined in (equation VI-2) and (equation VI-3) given by the intercept of the system (equation VI-4) and (equation VI-5).*

$-\boldsymbol{\Theta}^{-1}+\boldsymbol{\Psi}+\boldsymbol{\Theta}⊘\boldsymbol{\Gamma}^{.2}=\mathbf{0}_{q}$

$\left\{ -T\left| \boldsymbol{\Theta}\left( i,j \right) \right|^{2}+\boldsymbol{\Gamma}^{2}\left( i,j \right)+T\alpha^{2}\mathbf{A}^{2}\left( i,j \right)\boldsymbol{\Gamma}^{4}\left( i,j \right)=0 \right\}_{i,j=1}^{q}$

***Proof of Lemma 3***

With the hierarchical representation of the hermitian graphical LASSO precision-matrix $\boldsymbol{\Theta}$ prior, extension of Andrews and Mallows Lemma (Lemma 2), we attain a modified target function $\mathcal{L}\left( \boldsymbol{\Theta,\Gamma} \right)$ of the precision-matrix that builds in the combination of the sampled covariance-matrix $p\left( \left. \boldsymbol{\Psi} \right|\boldsymbol{\Theta} \right)$ Wishart likelihood dependent on the local quadratic approximation (improper probability density function of $\left. \boldsymbol{\Theta} \right|\boldsymbol{\Gamma}\times\boldsymbol{\Gamma}$) (equation VI-1). Other terms are related to the normalization constant of the precision-matrix $\left. \boldsymbol{\Theta} \right|\boldsymbol{\Gamma}$ Gaussian prior and the precision-matrix variances (weights) $\boldsymbol{\Gamma}$ Gamma prior. To build the target function $\mathcal{L}\left( \boldsymbol{\Theta,\Gamma} \right)$ we may employ the Bayes theorem for the improper posterior probability density function $p\left( \boldsymbol{\Theta},\boldsymbol{\Gamma} | \boldsymbol{\Psi} \right)$ based on the Wishart likelihood $p\left( \boldsymbol{\Psi} | \boldsymbol{\Theta} \right)$ and improper probability density function for $p\left( \left. \boldsymbol{\Theta} \right|\boldsymbol{\Gamma}\times\boldsymbol{\Gamma} \right)=p\left( \boldsymbol{\Theta} | \boldsymbol{\Gamma} \right)p\left( \boldsymbol{\Gamma} \right)$ (equation VI-6).

$\begin{matrix} p\left( \boldsymbol{\Theta},\boldsymbol{\Gamma} | \boldsymbol{\Psi} \right)\propto p\left( \boldsymbol{\Psi} | \boldsymbol{\Theta} \right)p\left( \boldsymbol{\Theta} | \boldsymbol{\Gamma} \right)p\left( \boldsymbol{\Gamma} \right) \\ p\left( \boldsymbol{\Theta},\boldsymbol{\Gamma} | \boldsymbol{\Psi} \right)\propto W_{q}^{\mathbb{C}}\left( \boldsymbol{\Psi} | T^{-1}\boldsymbol{\Theta}^{-1},T \right)\prod_{i,j=1}^{q} N_{1}\left( \left| \boldsymbol{\Theta}\left( i,j \right) \right| | 0,{\boldsymbol{\Gamma}^{2}\left( i,j \right)}/T \right)Ga\left( \boldsymbol{\Gamma}^{2}\left( i,j \right) | 1,T{\alpha^{2}\mathbf{A}^{2}\left( i,j \right)}/2 \right) \end{matrix}$

We then define the target function $\mathcal{L}\left( \boldsymbol{\Theta},\boldsymbol{\Gamma} \right)=-\log p\left( \boldsymbol{\Theta},\boldsymbol{\Gamma} | \boldsymbol{\Psi} \right)$ in (equation VI-6) as function dependent on the precision-matrix $\boldsymbol{\Theta}$ and precision-matrix variances $\boldsymbol{\Gamma}$ (equation VI-7),

$\mathcal{L}\left( \boldsymbol{\Theta},\boldsymbol{\Gamma} \right)=-T\log\left| \boldsymbol{\Theta} \right| +T tr\left( \boldsymbol{\Psi\Theta} \right)-\sum_{i,j=1}^{q} \log N_{1}\left( \left| \boldsymbol{\Theta}\left( i,j \right) \right| | 0,{\boldsymbol{\Gamma}^{2}\left( i,j \right)}/T \right)-\sum_{i,j=1}^{q} \log Ga\left( \boldsymbol{\Gamma}^{2}\left( i,j \right) | 1,T{\alpha_{\boldsymbol{\iota}}^{2}\mathbf{A}^{2}\left( i,j \right)}/2 \right)$

Given the definition of the univariate Gaussian probability density function in the third term of $\mathcal{L}\left( \boldsymbol{\Theta},\boldsymbol{\Gamma} \right)$ (equation VI-7) we may express (equation VI-8), where we omit other normalization terms not dependent on $\boldsymbol{\Theta}$ or $\boldsymbol{\Gamma}$.

$\sum_{i,j=1}^{q} \log N_{1}\left( \left| \boldsymbol{\Theta}\left( i,j \right) \right| | 0,{\boldsymbol{\Gamma}^{2}\left( i,j \right)}/T \right)=-\frac{1}{2}\sum_{i,j=1}^{q} \log\boldsymbol{\Gamma}^{2}\left( i,j \right)-\frac{T}{2}\sum_{i,j=1}^{q} {\left| \boldsymbol{\Theta}\left( i,j \right) \right|^{2}}/{\boldsymbol{\Gamma}^{2}\left( i,j \right)}$

Where above (equation VI-8), we may redefine the term involving $\boldsymbol{\Theta}$ as an L2 norm scaled by $\boldsymbol{\Gamma}$ (equation VI-9).

$\left\| \boldsymbol{\Theta}⊘\boldsymbol{\Gamma} \right\|_{2}^{2}=\sum_{ij=1}^{q} {\left| \boldsymbol{\Theta}\left( i,j \right) \right|^{2}}/{\boldsymbol{\Gamma}^{2}\left( i,j \right)}$

Given the definition of the univariate Gamma probability density function in the fourth term of $\mathcal{L}\left( \boldsymbol{\Theta},\boldsymbol{\Gamma} \right)$ (equation 6-7) we may express (equation VI-10), where we omit other normalization terms not dependent on $\boldsymbol{\Gamma}$.

$\sum_{i,j=1}^{q} \log Ga\left( \boldsymbol{\Gamma}^{2}\left( i,j \right) | 1,{T\alpha_{\boldsymbol{\iota}}^{2}\mathbf{A}^{2}\left( i,j \right)}/2 \right)=-\frac{T\alpha^{2}}{2}\sum_{i,j=1}^{q} \boldsymbol{\Gamma}^{2}\left( i,j \right)\mathbf{A}^{2}\left( i,j \right)$

Then employing the expressions (equation VI-8) and (equation VI-10) and substituting the L2 norm (equation VI-9) we may express the target function $\mathcal{L}\left( \boldsymbol{\Theta,\Gamma} \right)$ (equation VI-7) as a compact form of the precision-matrix $\boldsymbol{\Theta}$ precision-matrix, $\boldsymbol{\Gamma}$ and its prior (equation VI-11).

$\mathcal{L}\left( \boldsymbol{\Theta},\boldsymbol{\Gamma} \right)=-T\log\left| \boldsymbol{\Theta} \right| +T tr\left( \boldsymbol{\Psi\Theta} \right)+\frac{T}{2}\left\| \boldsymbol{\Theta}⊘\boldsymbol{\Gamma} \right\|_{2}^{2}+\frac{1}{2}\sum_{i,j=1}^{q} \log\boldsymbol{\Gamma}^{2}\left( i,j \right)+\frac{T\alpha^{2}}{2}\sum_{i,j=1}^{q} \boldsymbol{\Gamma}^{2}\left( i,j \right)\mathbf{A}^{2}\left( i,j \right)$

The concavity of the target function $\mathcal{L}\left( \boldsymbol{\Theta},\boldsymbol{\Gamma} \right)$ (equation VI-11) may be analyzed in terms of positive definiteness of the Hessian operator $\mathcal{H}$ computed by a block array of the second order matrix derivatives over the arguments $\boldsymbol{\Theta}$ and $\boldsymbol{\Gamma}$ (equation VI-12).

$\mathcal{H}\left( \mathcal{L}\left( \boldsymbol{\Theta},\boldsymbol{\Gamma} \right) \right)=\left[ \begin{matrix} \frac{\partial^{2}\mathcal{L}\left( \boldsymbol{\Theta},\boldsymbol{\Gamma} \right)}{\partial\boldsymbol{\Theta}\partial\boldsymbol{\Theta}} & \frac{\partial^{2}\mathcal{L}\left( \boldsymbol{\Theta},\boldsymbol{\Gamma} \right)}{\partial\boldsymbol{\Gamma}\partial\boldsymbol{\Theta}} \\ \frac{\partial^{2}\mathcal{L}\left( \boldsymbol{\Theta},\boldsymbol{\Gamma} \right)}{\partial\boldsymbol{\Theta}\partial\boldsymbol{\Gamma}} & \frac{\partial^{2}\mathcal{L}\left( \boldsymbol{\Theta},\boldsymbol{\Gamma} \right)}{\partial\boldsymbol{\Gamma}\partial\boldsymbol{\Gamma}} \end{matrix} \right]$

The block hessian operator (equation VI-12) is positive definite if and only if its diagonal blocks are positive definite. Given the expression of $\mathcal{L}\left( \boldsymbol{\Theta},\boldsymbol{\Gamma} \right)$ in (equation VI-11) we can deduce the structure of the hessian diagonal blocks from the following expressions (equation VI-13) and (equation VI-14).

$\frac{\partial^{2}\mathcal{L}\left( \boldsymbol{\Theta},\boldsymbol{\Gamma} \right)}{\partial\boldsymbol{\Theta}\partial\boldsymbol{\Theta}}=\frac{\partial^{2}}{\partial\boldsymbol{\Theta}\partial\boldsymbol{\Theta}}\left( -T\log\left| \boldsymbol{\Theta} \right| +T tr\left( \boldsymbol{\Psi\Theta} \right)+\frac{T}{2}\left\| \boldsymbol{\Theta}⊘\boldsymbol{\Gamma} \right\|_{2}^{2} \right)$

$\frac{\partial^{2}\mathcal{L}\left( \boldsymbol{\Theta},\boldsymbol{\Gamma} \right)}{\partial\boldsymbol{\Gamma}\partial\boldsymbol{\Gamma}}=\frac{\partial^{2}}{\partial\boldsymbol{\Gamma}\partial\boldsymbol{\Gamma}}\left( \frac{T}{2}\left\| \boldsymbol{\Theta}⊘\boldsymbol{\Gamma} \right\|_{2}^{2}+\frac{1}{2}\sum_{i,j=1}^{q} \log\boldsymbol{\Gamma}^{2}\left( i,j \right)+\frac{T\alpha^{2}}{2}\sum_{i,j=1}^{q} \boldsymbol{\Gamma}^{2}\left( i,j \right)\mathbf{A}^{2}\left( i,j \right) \right)$

Effectuating the derivatives, $\partial\boldsymbol{\Theta}\partial\boldsymbol{\Theta}$ for (equation VI-13) above we obtain the expression in (equation VI-15) and $\partial\boldsymbol{\Gamma}\partial\boldsymbol{\Gamma}$ for (equation VI-14) above we obtain the expression in (equation VI-16).

$\frac{\partial^{2}\mathcal{L}\left( \boldsymbol{\Theta},\boldsymbol{\Gamma} \right)}{\partial\boldsymbol{\Theta}\partial\boldsymbol{\Theta}}=T\boldsymbol{\Theta}^{-1}\otimes\boldsymbol{\Theta}^{-1} +\frac{T}{2} diag\left( vect\left( \mathbf{1}_{q}⊘\boldsymbol{\Gamma}^{.2} \right) \right)$

$\frac{\partial^{2}\mathcal{L}\left( \boldsymbol{\Theta},\boldsymbol{\Gamma} \right)}{\partial\boldsymbol{\Gamma}\partial\boldsymbol{\Gamma}}=3Tdiag\left( vect\left( \left| \boldsymbol{\Theta} \right|^{.2}⊘\boldsymbol{\Gamma}^{.4} \right) \right)-diag\left( vect\left( \mathbf{1}_{q}⊘\boldsymbol{\Gamma}^{.2} \right) \right)+T\alpha^{2}diag\left( vect\left( \mathbf{A}^{.2} \right) \right)$

For the derivative $\partial\boldsymbol{\Theta}\partial\boldsymbol{\Theta}$ in (equation VI-15) within the region of positive definiteness of $\boldsymbol{\Theta}\boldsymbol{≽}0$ the first term (Kronecker product obtained from $\log\left| \boldsymbol{\Theta} \right|$) is also positive definite $\boldsymbol{\Theta}^{-1}\otimes\boldsymbol{\Theta}^{-1}\boldsymbol{≽}0$, and since the elements $\boldsymbol{\Gamma}\left( i,j \right)>0$ are positive making the second term positive definite anywhere, then the whole expression (equation VI-15) is positive definite within the region of positive definiteness of $\boldsymbol{\Theta}\boldsymbol{≽}0$. The derivative $\partial\boldsymbol{\Gamma}\partial\boldsymbol{\Gamma}$ in (equation VI-16) is positive definite within the region of positive derivative elements $\partial\boldsymbol{\Gamma}\left( i,j \right)\partial\boldsymbol{\Gamma}\left( i,j \right)$ (equation VI-17) yielding the following set of inequalities (equation 6-18).

$\frac{\partial^{2}\mathcal{L}\left( \boldsymbol{\Theta},\boldsymbol{\Gamma} \right)}{\partial\boldsymbol{\Gamma}\left( i,j \right)\partial\boldsymbol{\Gamma}\left( i,j \right)}=\frac{\partial^{2}}{\partial\boldsymbol{\Gamma}\left( i,j \right)\partial\boldsymbol{\Gamma}\left( i,j \right)}\left( \frac{T}{2}\frac{\left| \boldsymbol{\Theta}\left( i,j \right) \right|^{2}}{\boldsymbol{\Gamma}^{2}\left( i,j \right)}+\frac{1}{2}\log\boldsymbol{\Gamma}^{2}\left( i,j \right)+\frac{T\alpha^{2}}{2}\boldsymbol{\Gamma}^{2}\left( i,j \right)\mathbf{A}^{2}\left( i,j \right) \right)=3T\frac{\left| \boldsymbol{\Theta}\left( i,j \right) \right|^{2}}{\boldsymbol{\Gamma}^{4}\left( i,j \right)}-\frac{1}{\boldsymbol{\Gamma}^{2}\left( i,j \right)}+T\alpha^{2}\mathbf{A}^{2}\left( i,j \right)$

$\left\{ 3T\frac{\left| \boldsymbol{\Theta}\left( i,j \right) \right|^{2}}{\boldsymbol{\Gamma}^{4}\left( i,j \right)}+-\frac{1}{\boldsymbol{\Gamma}^{2}\left( i,j \right)}+T\alpha^{2}\mathbf{A}^{2}\left( i,j \right)>0 \right\}_{i,j=1}^{q}$

Therefore, solutions for the minimum of $\mathcal{L}\left( \boldsymbol{\Theta},\boldsymbol{\Gamma} \right)$ in (equation VI-11) may be obtained by means of the derivatives $\partial\boldsymbol{\Theta}$ (equation VI-19) and $\partial\boldsymbol{\Gamma}$ (equation VI-20) in the region of the product space $\boldsymbol{\Theta}\times\boldsymbol{\Gamma}$.

$\frac{\partial\mathcal{L}\left( \boldsymbol{\Theta},\boldsymbol{\Gamma} \right)}{\partial\boldsymbol{\Theta}}=\frac{\partial}{\partial\boldsymbol{\Theta}}\left( -T\log\left| \boldsymbol{\Theta} \right| +T tr\left( \boldsymbol{\Psi\Theta} \right)+\frac{T}{2}\left\| \boldsymbol{\Theta}⊘\boldsymbol{\Gamma} \right\|_{2}^{2} \right)$

$\frac{\partial\mathcal{L}\left( \boldsymbol{\Theta},\boldsymbol{\Gamma} \right)}{\partial\boldsymbol{\Gamma}}=\frac{\partial}{\partial\boldsymbol{\Gamma}}\left( \frac{T}{2}\left\| \boldsymbol{\Theta}⊘\boldsymbol{\Gamma} \right\|_{2}^{2}+\frac{1}{2}\sum_{i,j=1}^{q} \log\boldsymbol{\Gamma}^{2}\left( i,j \right)+\frac{T\alpha^{2}}{2}\sum_{i,j=1}^{q} \boldsymbol{\Gamma}^{2}\left( i,j \right)\mathbf{A}^{2}\left( i,j \right) \right)$

Effectuating the derivatives, $\partial\boldsymbol{\Theta}$ for (equation VI-19) above we obtain the expression in (equation VI-21) and $\partial\boldsymbol{\Gamma}$ for (equation VI-20) above we obtain the expression in (equation VI-22).

$\frac{\partial\mathcal{L}\left( \boldsymbol{\Theta},\boldsymbol{\Gamma} \right)}{\partial\boldsymbol{\Theta}}=-T\left( \boldsymbol{\Theta}^{-1} \right)^{\mathcal{T}}+T\boldsymbol{\Psi}^{\mathcal{T}}+T\boldsymbol{\Theta}^{\mathcal{T}}⊘\boldsymbol{\Gamma}^{\mathbf{2}}$

$\frac{\partial\mathcal{L}\left( \boldsymbol{\Theta},\boldsymbol{\Gamma} \right)}{\partial\boldsymbol{\Gamma}}=-Tdiag\left( vect\left( \left| \boldsymbol{\Theta} \right|^{.2}⊘\boldsymbol{\Gamma}^{.3} \right) \right)+diag\left( vect\left( \mathbf{1}_{q}⊘\boldsymbol{\Gamma} \right) \right)+T\alpha^{2}diag\left( vect\left( \boldsymbol{\Gamma}⨀\mathbf{A}^{.\mathbf{2}} \right) \right)$

It can be checked that for the derivative $\partial\boldsymbol{\Gamma}$ (equation VI-22) the zero also makes positive the hessian $\partial\boldsymbol{\Gamma}\partial\boldsymbol{\Gamma}$ (equation 2-16). By substituting this zero given in (equation VI-23), in the hessian (equation 2-16) this yields a positive-definite expression (equation VI-24) anywhere the elements $\boldsymbol{\Gamma}\left( i,j \right)>0$ are positive.

$-Tdiag\left( vect\left( \left| \boldsymbol{\Theta} \right|^{.2}⊘\boldsymbol{\Gamma}^{.4} \right) \right)+diag\left( vect\left( \mathbf{1}_{q}⊘\boldsymbol{\Gamma}^{.\mathbf{2}} \right) \right)+T\alpha^{2}diag\left( vect\left( \mathbf{A}^{.2} \right) \right)=0$

$\frac{\partial^{2}\mathcal{L}\left( \boldsymbol{\Theta},\boldsymbol{\Gamma} \right)}{\partial\boldsymbol{\Gamma}\partial\boldsymbol{\Gamma}}=2diag\left( vect\left( \mathbf{1}_{q}⊘\boldsymbol{\Gamma} \right) \right)+4T\alpha^{2}diag\left( vect\left( \mathbf{A}^{.\mathbf{2}} \right) \right)\boldsymbol{≽}0$

From these derivatives in (equation VI-21) and (equation VI-22) the target function $\mathcal{L}\left( \boldsymbol{\Theta},\boldsymbol{\Gamma} \right)$ and possess a minimum in the point due to the systems in (equation VI-25) and (equation VI-26).

$-\boldsymbol{\Theta}^{-1}+\boldsymbol{\Psi}+\boldsymbol{\Theta}⊘\boldsymbol{\Gamma}^{\mathbf{2}}=\mathbf{0}_{q}$

$\left\{ -T\left| \boldsymbol{\Theta}\left( i,j \right) \right|^{2}+\boldsymbol{\Gamma}^{2}\left( i,j \right)+T\alpha^{2}\mathbf{A}^{2}\left( i,j \right)\boldsymbol{\Gamma}^{4}\left( i,j \right)=0 \right\}_{i,j=1}^{q}$

This minimum is unique due to the convexity within the region defined in the product space $\boldsymbol{\Theta}\times\boldsymbol{\Gamma}$ since the following inequalities are fulfilled (equation VI-27) and (equation VI-28).

$\left\{ \boldsymbol{\Theta}\boldsymbol{≽}0\boldsymbol{,}\boldsymbol{\Gamma}\boldsymbol{≽}0 \right\}$

$\left\{ 3T\frac{\left| \boldsymbol{\Theta}\left( i,j \right) \right|^{2}}{\boldsymbol{\Gamma}^{4}\left( i,j \right)}+-\frac{1}{\boldsymbol{\Gamma}^{2}\left( i,j \right)}+T\alpha^{2}\mathbf{A}^{2}\left( i,j \right)>0 \right\}_{i,j=1}^{q}$

For every element $\boldsymbol{\Gamma}\left( i,j \right)$ of the fourth order system (equation VI-26) the minimum is given by the discriminant formula, roots of second order polynomials (equation VI-29). In matrix form this solution is expressed as (equation VI-30).

$\boldsymbol{\Gamma}\left( i,j \right)=\left( \left( -1+\left( 1+4T^{2}\alpha^{2}\mathbf{A}^{2}\left( i,j \right)\left| \boldsymbol{\Theta}\left( i,j \right) \right|^{2} \right)^{\frac{1}{2}} \right)/\left( 2T\alpha^{2}\mathbf{A}^{2}\left( i,j \right) \right) \right)^{\frac{1}{2}}$

$\boldsymbol{\Gamma}=\left( \left( -\mathbf{1}_{q}+\left( \mathbf{1}_{q}+4T^{2}\alpha^{2}\mathbf{A}^{.2}\odot\left| \boldsymbol{\Theta} \right|^{.2} \right)^{.\frac{1}{2}} \right)⊘\left( 2T\alpha^{2}\mathbf{A}^{.2} \right) \right)^{.\frac{1}{2}}$

Given the positive definiteness of $\mathbf{A}$ and $\boldsymbol{\Theta}$, it can also be deduced that the solution $\boldsymbol{\Gamma}$ (equation VI-30) is positive definite by following the steps in proposition 3 of Lemma 4. $∎$

According to this local quadratic approximation, we can define a new random matrix through the scaling transformation $\tilde{\boldsymbol{\Theta}}=\boldsymbol{\Theta}⊘\boldsymbol{\Gamma}$ (standard precision-matrix), so that its prior is a Gibbs probability density function of the squared L2 norm (equation VI-31).

$p\left( \tilde{\boldsymbol{\Theta}} \right)\propto e^{-\frac{T}{2}\left\| \tilde{\boldsymbol{\Theta}} \right\|_{2}^{2}}$

For the solution based of this standard precision-matrix we also standardize the sampled covariance-matrix due to the transformation $\tilde{\boldsymbol{\Psi}}=\left( \boldsymbol{\Psi}^{-1}⊘\boldsymbol{\Gamma} \right)^{-1}$ that keeps the stochastic properties of the Wishart distribution when conditioned to $\tilde{\boldsymbol{\Theta}}$.

## Standardization of the first-type likelihood with hgLASSO LQA prior (Lemma 4)

***Lemma 4 (standardization of the Wishart distribution):*** *Let* $\boldsymbol{\Psi}$ *and* $\tilde{\boldsymbol{\Psi}}$ *(referred as standardized sampled covariance matrix) be* $\left( q\times q \right)$ *hermitian random matrices with complex-valued Wishart probability density functions* $W_{q}^{\mathbb{C}}$ *defined in (equation VII-1) and (equation VII-2). The Wishart* $W_{q}^{\mathbb{C}}$ *has* $T$ *degrees of freedom and positive definite hermitic scale matrices,* $\boldsymbol{\Sigma}$ *for (equation VII-1), and* $\tilde{\boldsymbol{\Sigma}}=\left( \boldsymbol{\Sigma}^{-1}⊘\boldsymbol{\Gamma} \right)^{-1}$ *(referred as standardized scale matrix) for (equation VII-2), with* $\boldsymbol{\Gamma}$ *being a* $\left( q\times q \right)$ *positive definite matrix of positive weights.*

$p\left( \boldsymbol{\Psi} \right)\mathbf{=}W_{q}^{\mathbb{C}}\left( \boldsymbol{\Psi} | \boldsymbol{\Sigma},T \right)\propto\left| \boldsymbol{\Psi} \right|^{\left( T-q \right)}\left| \boldsymbol{\Sigma} \right|^{-T}e^{-tr\left( \boldsymbol{\Sigma}^{-1}\boldsymbol{\Psi} \right)}$

$p\left( \tilde{\boldsymbol{\Psi}} \right)\mathbf{=}W_{q}^{\mathbb{C}}\left( \tilde{\boldsymbol{\Psi}} | \tilde{\boldsymbol{\Sigma}},T \right)\propto\left| \tilde{\boldsymbol{\Psi}} \right|^{\left( T-q \right)}\left| \tilde{\boldsymbol{\Sigma}} \right|^{-T}e^{-tr\left( {\tilde{\boldsymbol{\Sigma}}}^{-1}\tilde{\boldsymbol{\Sigma}} \right)}$

*Then for* $\boldsymbol{\Phi}$ *(referred de-standardization of* $\tilde{\boldsymbol{\Psi}}$*) defined by the relationship to the standard sampled covariance-matrix* $\tilde{\boldsymbol{\Psi}}=\left( \boldsymbol{\Phi}^{-1}⊘\boldsymbol{\Gamma} \right)^{-1}$ *(or* $\boldsymbol{\Phi=}\left( {\tilde{\boldsymbol{\Psi}}}^{-1}\odot\boldsymbol{\Gamma} \right)^{-1}$*), it can be verified:*

*a) All entries* $\boldsymbol{\Phi}^{-1}\left( i,j \right)$ *of the de-standardization inverse* $\boldsymbol{\Phi}^{-1}$ *keep the Wishart probability density function independency property: they are stochastically independent among them and from the set* $\left\{ \boldsymbol{\Phi}\left( i^{'},j^{'} \right):\left( i^{'},j^{'} \right)\neq\left( i,j \right) \right\}$*.*

*b) All entries* $\boldsymbol{\Phi}^{-1}\left( i,j \right)$ *of the de-standardization inverse* $\boldsymbol{\Phi}^{-1}$ *keep the Wishart marginal probability density function: complex-valued inverse Gamma probability density function with parameter of shape* $\left( T-q+1 \right)$ *and scale* $\boldsymbol{\Sigma}^{-1}\left( i,j \right)$*.*

***Proof of Lemma 4***

**Proposition 1**

If $\boldsymbol{\Psi}$ is a $\left( q\times q \right)$ complex-valued random matrix with Wishart probability density function (equation VII-1) of $T$ degrees of freedom and positive definite scale matrix $\boldsymbol{\Sigma}$. Then the random matrix obtained from the consecutive rows and columns permutation operations, has also Wishart probability density function of $T$ degrees of freedom and positive definite scale matrix (equation VII-3).

$\left( \mathbf{P}_{j\leftrightarrow j^{'}}^{col}\mathbf{P}_{i\leftrightarrow i^{'}}^{row}\boldsymbol{\Sigma}^{-1} \right)^{-1}$

**Proof of Proposition 1:**

For the Wishart probability density function of the random matrix $\boldsymbol{\Psi}$ that can be expressed as (equation VII-4) it holds that the determinants and trace terms are invariant to consecutive rows and columns permutation operations (equation VII-3), represented as the identities (equation VII-5), (equation VII-6), and (equation VII-7).

$W_{q}^{\mathbb{C}}\left( \boldsymbol{\Psi} | \boldsymbol{\Sigma},T \right)$ $\propto$ $\left| \boldsymbol{\Psi} \right|^{\left( T-q \right)}\left| \boldsymbol{\Sigma} \right|^{-T}e^{-tr\left( \boldsymbol{\Sigma}^{-1}\boldsymbol{\Psi} \right)}$

$\left| \boldsymbol{\Sigma}^{-1} \right|=\left| \mathbf{P}_{j\leftrightarrow j^{'}}^{col}\mathbf{P}_{i\leftrightarrow i^{'}}^{row}\boldsymbol{\Sigma}^{-1} \right|$

$\left| \boldsymbol{\Psi} \right|=\left| \mathbf{P}_{j\leftrightarrow j^{'}}^{col}\mathbf{P}_{i\leftrightarrow i^{'}}^{row}\boldsymbol{\Psi} \right|$

$tr\left( \boldsymbol{\Sigma}^{-1}\boldsymbol{\Psi} \right)=tr\left( \mathbf{P}_{j\leftrightarrow j^{'}}^{col}\mathbf{P}_{i\leftrightarrow i^{'}}^{row}\boldsymbol{\Sigma}^{-1}\mathbf{P}_{j\leftrightarrow j^{'}}^{col}\mathbf{P}_{i\leftrightarrow i^{'}}^{row}\boldsymbol{\Psi} \right)$

Due to identities (equation VII-5), (equation VII-6), and (equation VII-7) the Wishart $W_{q}^{\mathbb{C}}$ probability density function values for the original and permuted random matrices are identic (equation VII-8).

$W_{q}^{\mathbb{C}}\left( \boldsymbol{\Psi} | \boldsymbol{\Sigma},m \right)=W_{q}^{\mathbb{C}}\left( \mathbf{P}_{j\leftrightarrow j^{'}}^{col}\mathbf{P}_{i\leftrightarrow i^{'}}^{row}\boldsymbol{\Psi} | \left( \mathbf{P}_{j\leftrightarrow j^{'}}^{col}\mathbf{P}_{i\leftrightarrow i^{'}}^{row}\boldsymbol{\Sigma}^{-1} \right)^{-1},T \right)$

By definition, the Wishart is a joint probability density function of the set of random matrix entries in $\boldsymbol{\Psi}$ (equation VII-9). For permutations this joint probability density function would be invariant, as represented by same Wishart (equation VII-10).

$p\left( \left\{ \boldsymbol{\Psi}\left( i,j \right):i,j=1\ldots q \right\} \right)=p\left( \boldsymbol{\Psi} \right)=W_{q}^{\mathbb{C}}\left( \boldsymbol{\Psi} | \boldsymbol{\Sigma},T \right)$

$p\left( \left\{ \mathbf{P}_{j\leftrightarrow j^{'}}^{col}\mathbf{P}_{i\leftrightarrow i^{'}}^{row}\boldsymbol{\Psi}\left( i,j \right):i,j=1\ldots q \right\} \right)=p\left( \mathbf{P}_{j\leftrightarrow j^{'}}^{col}\mathbf{P}_{i\leftrightarrow i^{'}}^{row}\boldsymbol{\Psi} \right)=W_{q}^{\mathbb{C}}\left( \boldsymbol{\Psi} | \boldsymbol{\Sigma},T \right)$

From the identity between Wishart probability density functions (equation VII-8), it is clear that the permuted random matrix follows also a Wishart probability density function with permuted scale matrix (equation VII-11).

$p\left( \mathbf{P}_{j\leftrightarrow j^{'}}^{col}\mathbf{P}_{i\leftrightarrow i^{'}}^{row}\boldsymbol{\Psi} \right)=W_{q}^{\mathbb{C}}\left( \mathbf{P}_{j\leftrightarrow j^{'}}^{col}\mathbf{P}_{i\leftrightarrow i^{'}}^{row}\boldsymbol{\Psi} | \left( \mathbf{P}_{j\leftrightarrow j^{'}}^{col}\mathbf{P}_{i\leftrightarrow i^{'}}^{row}\boldsymbol{\Sigma}^{-1} \right)^{-1},T \right)∎$

An element $\boldsymbol{\Psi}\left( i,j \right)$ of a random matrix $\boldsymbol{\Psi}$ with Wishart probability density function (equation VII-4), the conditional probability density function regarding the remaining elements $\left\{ \bar{\boldsymbol{\Psi}\left( i,j \right)} \right\}=\left\{ \boldsymbol{\Psi}\left( i^{'},j^{'} \right):\left( i^{'},j^{'} \right)\neq\left( i,j \right) \right\}$ may be expressed as (equation VII-12).

$p\left( \boldsymbol{\Psi}\left( i,j \right) | \left\{ \bar{\boldsymbol{\Psi}\left( i,j \right)} \right\} \right)\propto\left| \boldsymbol{\Psi} \right|^{\left( T-q \right)}e^{-tr\left( \boldsymbol{\Sigma}^{-1}\boldsymbol{\Psi} \right)}$

An explicit form of this conditional probability density function for $\boldsymbol{\Psi}\left( i,j \right)$ (equation VII-12) may be obtained by applying the consecutive permutation operations (equation VII-13) and then consider the conditional conditional probability density function of the first element (equation VII-14).

$\mathbf{P}_{j\leftrightarrow1}^{col}\mathbf{P}_{i\leftrightarrow1}^{row}\boldsymbol{\Psi}$

$p\left( \mathbf{P}_{j\leftrightarrow1}^{col}\mathbf{P}_{i\leftrightarrow1}^{row}\boldsymbol{\Psi}\left( 1,1 \right) | \left\{ \bar{\mathbf{P}_{j\leftrightarrow1}^{col}\mathbf{P}_{i\leftrightarrow1}^{row}\boldsymbol{\Psi}\left( 1,1 \right)} \right\} \right)\propto\left| \mathbf{P}_{j\leftrightarrow1}^{col}\mathbf{P}_{i\leftrightarrow1}^{row}\boldsymbol{\Psi} \right|^{\left( T-q \right)}e^{-tr\left( \mathbf{P}_{j\leftrightarrow1}^{col}\mathbf{P}_{i\leftrightarrow1}^{row}\boldsymbol{\Sigma}^{-1}\mathbf{P}_{j\leftrightarrow1}^{col}\mathbf{P}_{i\leftrightarrow1}^{row}\boldsymbol{\Psi} \right)}$

Due to Proposition 1 the probability density function of the permuted random matrix (equation VII-13) is also Wishart, and therefore, for the element (equation VII-14) the conditional probability density function can be expressed as (equation VII-11). Without losing generality we can consider first the element in the original random matrix $\boldsymbol{\Psi}\left( 1,1 \right)$ (equaion VII-15) and then this result will also apply for all $\boldsymbol{\Psi}\left( i,j \right)$ (equation VII-12).

$p\left( \boldsymbol{\Psi}\left( 1,1 \right) | \left\{ \bar{\boldsymbol{\Psi}\left( 1,1 \right)} \right\} \right)\propto\left| \boldsymbol{\Psi} \right|^{\left( T-q \right)}e^{-tr\left( \boldsymbol{\Sigma}^{-1}\boldsymbol{\Psi} \right)}$

Partitioning the random matrix $\boldsymbol{\Psi}$ and $\boldsymbol{\Sigma}^{-1}$ into the following block structures (equation VII-16) and (equation VII-17) we can find a simplified expression of the conditional probability density function (equation VII-15).

$\boldsymbol{\Psi}=\left( \begin{matrix} \boldsymbol{\Psi}\left( 1,1 \right) & \boldsymbol{\Psi}\left( 1,2 \right) \\ \boldsymbol{\Psi}\left( 2,1 \right) & \boldsymbol{\Psi}\left( 2,2 \right) \end{matrix} \right)$

$\boldsymbol{\Sigma}^{-1}=\left( \begin{matrix} \boldsymbol{\Sigma}^{-1}\left( 1,1 \right) & \boldsymbol{\Sigma}^{-1}\left( 1,2 \right) \\ \boldsymbol{\Sigma}^{-1}\left( 2,1 \right) & \boldsymbol{\Sigma}^{-1}\left( 2,2 \right) \end{matrix} \right)$

From this block structures (equation VII-16) and (equation VII-17) we can deduce the form of the term in the exponent $tr\left( \boldsymbol{\Sigma}^{-1}\boldsymbol{\Psi} \right)$ (equation VII-15) due to the product $\boldsymbol{\Sigma}^{-1}\boldsymbol{\Psi}$ (equation VII-18) that yields an expression sparable for $\boldsymbol{\Psi}\left( 1,1 \right)$ (equation VII-19). From the block structure (equation VII-16) the detrminant $\left| \boldsymbol{\Psi} \right|$ (equation VII-15) also yields an expression sparable for $\boldsymbol{\Psi}\left( 1,1 \right)$ (equation VII-20).

$\boldsymbol{\Sigma}^{-1}\boldsymbol{\Psi}=\left( \begin{matrix} \boldsymbol{\Sigma}^{-1}\left( 1,1 \right)\boldsymbol{\Psi}\left( 1,1 \right)+\boldsymbol{\Sigma}^{-1}\left( 1,2 \right)\boldsymbol{\Psi}\left( 2,1 \right) & \boldsymbol{\Sigma}^{-1}\left( 1,1 \right)\boldsymbol{\Psi}\left( 1,2 \right)+\boldsymbol{\Sigma}^{-1}\left( 1,2 \right)\boldsymbol{\Psi}\left( 2,2 \right) \\ \boldsymbol{\Sigma}^{-1}\left( 2,1 \right)\boldsymbol{\Psi}\left( 1,1 \right)+\boldsymbol{\Sigma}^{-1}\left( 2,2 \right)\boldsymbol{\Psi}\left( 2,1 \right) & \boldsymbol{\Sigma}^{-1}\left( 2,1 \right)\boldsymbol{\Psi}\left( 1,2 \right)+\boldsymbol{\Sigma}^{-1}\left( 2,2 \right)\boldsymbol{\Psi}\left( 2,2 \right) \end{matrix} \right)$

$tr\left( \boldsymbol{\Sigma}^{-1}\boldsymbol{\Psi} \right)=\boldsymbol{\Sigma}^{-1}\left( 1,1 \right)\boldsymbol{\Psi}\left( 1,1 \right)+\boldsymbol{\Sigma}^{-1}\left( 1,2 \right)\boldsymbol{\Psi}\left( 2,1 \right)+tr\left( \boldsymbol{\Sigma}^{-1}\left( 2,1 \right)\boldsymbol{\Psi}\left( 1,2 \right)+\boldsymbol{\Sigma}^{-1}\left( 2,2 \right)\boldsymbol{\Psi}\left( 2,2 \right) \right)$

$\left| \boldsymbol{\Psi} \right|=\left| \boldsymbol{\Psi}\left( 2,2 \right) \right|\left( \boldsymbol{\Psi}\left( 1,1 \right)-\boldsymbol{\Psi}\left( 1,2 \right)\left( \boldsymbol{\Psi}\left( 2,2 \right) \right)^{-1}\boldsymbol{\Psi}\left( 2,1 \right) \right)$

We therefore may express the conditional probability density function as (equation VII-21) by completing the exponent with the term $\boldsymbol{\Psi}\left( 1,2 \right)\left( \boldsymbol{\Psi}\left( 2,2 \right) \right)^{-1}\boldsymbol{\Psi}\left( 2,1 \right)$.

$p\left( \boldsymbol{\Psi}\left( 1,1 \right) | \left\{ \bar{\boldsymbol{\Psi}\left( 1,1 \right)} \right\} \right)\propto\left( \boldsymbol{\Psi}\left( 1,1 \right)-\boldsymbol{\Psi}\left( 1,2 \right)\left( \boldsymbol{\Psi}\left( 2,2 \right) \right)^{-1}\boldsymbol{\Psi}\left( 2,1 \right) \right)^{\left( T-q \right)}e^{-\boldsymbol{\Sigma}^{-1}\left( 1,1 \right)\left( \boldsymbol{\Psi}\left( 1,1 \right)-\boldsymbol{\Psi}\left( 1,2 \right)\left( \boldsymbol{\Psi}\left( 2,2 \right) \right)^{-1}\boldsymbol{\Psi}\left( 2,1 \right) \right)}$

Due to the structure of the block inverse for $\boldsymbol{\Psi}^{-\mathbf{1}}$ (equation VII-22), the argument of the conditional probability density function above (equation VII-21) is the element $\boldsymbol{\Psi}^{-\mathbf{1}}\left( 1,1 \right)$ of this block inverse $\boldsymbol{\Psi}^{-\mathbf{1}}$ (equation VII-23).

$\boldsymbol{\Psi}^{-\mathbf{1}}=\left( \begin{matrix} \frac{\mathbf{1}}{\boldsymbol{\Psi}\left( 1,1 \right)-\boldsymbol{\Psi}\left( 1,2 \right)\left( \boldsymbol{\Psi}\left( 2,2 \right) \right)^{-1}\boldsymbol{\Psi}\left( 2,1 \right)} & -\frac{\left( \boldsymbol{\Psi}\left( 2,2 \right) \right)^{-1}\boldsymbol{\Psi}\left( 2,1 \right)}{\boldsymbol{\Psi}\left( 1,1 \right)-\boldsymbol{\Psi}\left( 1,2 \right)\left( \boldsymbol{\Psi}\left( 2,2 \right) \right)^{-1}\boldsymbol{\Psi}\left( 2,1 \right)} \\ -\frac{\left( \boldsymbol{\Psi}\left( 2,2 \right) \right)^{-1}\boldsymbol{\Psi}\left( 2,1 \right)}{\boldsymbol{\Psi}\left( 1,1 \right)-\boldsymbol{\Psi}\left( 1,2 \right)\left( \boldsymbol{\Psi}\left( 2,2 \right) \right)^{-1}\boldsymbol{\Psi}\left( 2,1 \right)} & \left( \boldsymbol{\Psi}\left( 2,2 \right) \right)^{-1}+\frac{\left( \boldsymbol{\Psi}\left( 2,2 \right) \right)^{-1}\boldsymbol{\Psi}\left( 2,1 \right)\boldsymbol{\Psi}\left( 1,2 \right)\left( \boldsymbol{\Psi}\left( 2,2 \right) \right)^{-1}}{\boldsymbol{\Psi}\left( 1,1 \right)-\boldsymbol{\Psi}\left( 1,2 \right)\left( \boldsymbol{\Psi}\left( 2,2 \right) \right)^{-1}\boldsymbol{\Psi}\left( 2,1 \right)} \end{matrix} \right)$

$\boldsymbol{\Psi}^{-\mathbf{1}}\left( 1,1 \right)=\frac{\mathbf{1}}{\boldsymbol{\Psi}\left( 1,1 \right)-\boldsymbol{\Psi}\left( 1,2 \right)\left( \boldsymbol{\Psi}\left( 2,2 \right) \right)^{-1}\boldsymbol{\Psi}\left( 2,1 \right)}$

From these relationships (equation VII-22) and (equation VII-23) it is deduced that any element in the random matrix $\boldsymbol{\Psi}^{-\mathbf{1}}$, a random variable $\boldsymbol{\Psi}^{-\mathbf{1}}\left( 1,1 \right)$ is conditionally independent from $\left\{ \bar{\boldsymbol{\Psi}\left( 1,1 \right)} \right\}$ and has complex-valued inverse Gamma probability density function with shape parameter $\left( T-q \right)$ and scale parameter $\boldsymbol{\Sigma}^{-1}\left( 1,1 \right)$ (equation VII-24).

$p\left( \boldsymbol{\Psi}^{-\mathbf{1}}\left( 1,1 \right) \right)\propto\left( \boldsymbol{\Psi}^{-\mathbf{1}}\left( 1,1 \right) \right)^{-\left( T-q \right)}e^{-\frac{\boldsymbol{\Sigma}^{-1}\left( 1,1 \right)}{\boldsymbol{\Psi}^{-\mathbf{1}}\left( 1,1 \right)}}$

Now we define, 1) a random matrix $\tilde{\boldsymbol{\Psi}}$ (referred as standardized sampled covariance matrix) with Wishart probability density function that can be expressed as (equation VII-25) with scale matrix $\tilde{\boldsymbol{\Sigma}}$, defined by the tranformation of the element $\boldsymbol{\Sigma}^{-1}\left( 1,1 \right)$ from the scale matrix for the Wishart probability density function of the random matrix $\boldsymbol{\Psi}$ (equation VII-4), by a positive number $\boldsymbol{\Gamma}\left( 1,1 \right)$ (equation VII-26), and 2) another random matrix $\boldsymbol{\Phi}$ through the relationsship with the standardized sampled covariance matrix $\tilde{\boldsymbol{\Psi}}$ (equation VII-27).

$W_{q}^{\mathbb{C}}\left( \tilde{\boldsymbol{\Psi}} | \tilde{\boldsymbol{\Sigma}},T \right)\propto\left| \tilde{\boldsymbol{\Psi}} \right|^{\left( T-q \right)}\left| \tilde{\boldsymbol{\Sigma}} \right|^{-T}e^{-tr\left( {\tilde{\boldsymbol{\Sigma}}}^{-1}\tilde{\boldsymbol{\Sigma}} \right)}$

$\tilde{\boldsymbol{\Sigma}}=\left( \begin{matrix} \frac{\boldsymbol{\Sigma}^{-1}\left( 1,1 \right)}{\boldsymbol{\Gamma}\left( 1,1 \right)} & \boldsymbol{\Sigma}^{-1}\left( 1,2 \right) \\ \boldsymbol{\Sigma}^{-1}\left( 2,1 \right) & \boldsymbol{\Sigma}^{-1}\left( 1,1 \right) \end{matrix} \right)^{-1}$

$\tilde{\boldsymbol{\Psi}}=\left( \begin{matrix} \frac{\boldsymbol{\Phi}^{-1}\left( 1,1 \right)}{\boldsymbol{\Gamma}\left( 1,1 \right)} & \boldsymbol{\Phi}^{-1}\left( 1,2 \right) \\ \boldsymbol{\Phi}^{-1}\left( 2,1 \right) & \boldsymbol{\Phi}^{-1}\left( 2,2 \right) \end{matrix} \right)^{-1}$

**Proposition 2**

The element $\boldsymbol{\Phi}^{-1}\left( 1,1 \right)$ of the matrix $\boldsymbol{\Phi}^{-1}$ (equation VII-27) has identical marginal pdf that the element $\boldsymbol{\Psi}^{-\mathbf{1}}\left( 1,1 \right)$ of the matrix $\boldsymbol{\Psi}$, when $\tilde{\boldsymbol{\Psi}}$ has Wishart probability density function (equation VII-25) with $T$ degrees of freedom and scale matrix $\tilde{\boldsymbol{\Sigma}}$ (equation VII-26).

**Proof of Proposition 2**

Using analogous representation to (equation VII-12) for the conditional probability density function of $\tilde{\boldsymbol{\Psi}}\left( 1,1 \right)$ given the remaining elements $\left\{ \bar{\tilde{\boldsymbol{\Psi}}\left( 1,1 \right)} \right\}=\left\{ \tilde{\boldsymbol{\Psi}}\left( i^{'},j^{'} \right):\left( i^{'},j^{'} \right)\neq\left( i,j \right) \right\}$ (equation VII-28).

$p\left( \tilde{\boldsymbol{\Psi}}\left( 1,1 \right) | \left\{ \bar{\tilde{\boldsymbol{\Psi}}\left( 1,1 \right)} \right\} \right)\propto\left| \tilde{\boldsymbol{\Psi}} \right|^{\left( T-q \right)}e^{-tr\left( {\tilde{\boldsymbol{\Sigma}}}^{-1}\boldsymbol{\Psi} \right)}$

Given the property of the Wishart probability density function deduced before in **Proposition 1** (equation VII-24) it is clear that the random variable ${\tilde{\boldsymbol{\Psi}}}^{-\mathbf{1}}\left( 1,1 \right)$ is independent from $\left\{ \bar{\tilde{\boldsymbol{\Psi}}\left( 1,1 \right)} \right\}$ and it has inverse Gamma probability density function (equation VII-29) with shape parameter $\left( T-q \right)$ and scale parameter ${\tilde{\boldsymbol{\Sigma}}}^{-1}\left( 1,1 \right)$ (equation VII-26).

$p\left( {\tilde{\boldsymbol{\Psi}}}^{-\mathbf{1}}\left( 1,1 \right) \right)\propto\left( {\tilde{\boldsymbol{\Psi}}}^{-\mathbf{1}}\left( 1,1 \right) \right)^{-\left( T-q \right)}e^{-\frac{{\tilde{\boldsymbol{\Sigma}}}^{-1}\left( 1,1 \right)}{{\tilde{\boldsymbol{\Psi}}}^{-\mathbf{1}}\left( 1,1 \right)}}$

By definition of $\boldsymbol{\Phi}^{-1}\left( 1,1 \right)=\boldsymbol{\Gamma}\left( 1,1 \right){\tilde{\boldsymbol{\Psi}}}^{-\mathbf{1}}\left( 1,1 \right)$ (equation VII-27), it holds that $\boldsymbol{\Phi}^{-1}\left( 1,1 \right)$ is also independent and it has inverse Gamma probability density function (equation VII-29) with shape parameter $\left( m-q \right)$ and scale parameter $\boldsymbol{\Gamma}\left( 1,1 \right){\tilde{\boldsymbol{\Sigma}}}^{-1}\left( 1,1 \right)=\boldsymbol{\Sigma}^{-1}\left( 1,1 \right)$ (equation VII-26).

$p\left( \boldsymbol{\Phi}^{-1}\left( 1,1 \right) \right)\propto\left( \boldsymbol{\Phi}^{-1}\left( 1,1 \right) \right)^{-\left( T-q \right)}e^{-\frac{\boldsymbol{\Sigma}^{-1}\left( 1,1 \right)}{\boldsymbol{\Phi}^{-1}\left( 1,1 \right)}}$

From the relationshsip of $\boldsymbol{\Phi}^{-1}\left( 1,1 \right)$ with $\boldsymbol{\Phi}\left( 1,1 \right)$ due to the block inverse, the probability density function (equation VII-29) can be employed to express the conditional probability density function for $\boldsymbol{\Phi}\left( 1,1 \right)$ (equation VII-30) regarding the remaining elements $\left\{ \bar{\boldsymbol{\Phi}\left( 1,1 \right)} \right\}$, which yields the same Wishart marginal distribution as for $\boldsymbol{\Psi}\left( 1,1 \right)$ (equation VII-21).

$p\left( \boldsymbol{\Phi}\left( 1,1 \right) | \left\{ \bar{\boldsymbol{\Phi}\left( 1,1 \right)} \right\} \right)\propto\left( \boldsymbol{\Phi}\left( 1,1 \right)-\boldsymbol{\Phi}\left( 1,2 \right)\left( \boldsymbol{\Phi}\left( 2,2 \right) \right)^{-1}\boldsymbol{\Phi}\left( 2,1 \right) \right)^{\left( T-q \right)}e^{-\boldsymbol{\Sigma}^{-1}\left( 1,1 \right)\left( \boldsymbol{\Phi}\left( 1,1 \right)-\boldsymbol{\Phi}\left( 1,2 \right)\left( \boldsymbol{\Phi}\left( 2,2 \right) \right)^{-1}\boldsymbol{\Phi}\left( 2,1 \right) \right)}∎$

Due to **Proposition 1** we can iteratively apply the scaling operation described in (equation VII-26) and (equation VII-27) for all elements $\tilde{\boldsymbol{\Psi}}=\left( \boldsymbol{\Phi}^{-1}⊘\boldsymbol{\Gamma} \right)^{-1}$, and with $\tilde{\boldsymbol{\Psi}}$ described by $p\left( \tilde{\boldsymbol{\Psi}} \right)=W_{q}^{\mathbb{C}}\left( \tilde{\boldsymbol{\Psi}} | \tilde{\boldsymbol{\Sigma}},T \right)$ Wishart probability density function of $T$ degrees of freedom and positive definite scale matrix $\tilde{\boldsymbol{\Sigma}}=\left( \boldsymbol{\Sigma}^{-1}⊘\boldsymbol{\Gamma} \right)^{-1}$. In this case the random matrix $\boldsymbol{\Phi}^{-1}$ identical marginal stochastic properties to $\boldsymbol{\Psi}^{-\mathbf{1}}$.

To complete the proof it is enough to show that the scale matrix $\tilde{\boldsymbol{\Sigma}}=\left( \boldsymbol{\Sigma}^{-1}⊘\boldsymbol{\Gamma} \right)^{-1}$ of the Wishart probability density function keeps being positive definite, as we can check from the following proposition which is a Corollary of Schur product theorem [6].

**Proposition 3**

If $\boldsymbol{\Sigma}$ and $\boldsymbol{\Gamma}$ are positive definite matrices then the matrix $\tilde{\boldsymbol{\Sigma}}=\left( \boldsymbol{\Sigma}^{-1}⊘\boldsymbol{\Gamma} \right)^{-1}$ from the scaling operation is also positive definite.

**Proof of Proposition 3**

If $\boldsymbol{\Sigma}$ is positive definite so it is also the inverse $\boldsymbol{\Sigma}^{-1}$. Then, let’s analyze the positive definiteness of the Hadamard scaling $\mathbf{1}_{q}⊘\boldsymbol{\Gamma}$. This Hadamard scaling can be expressed as an elementwise exponentiation $\mathbf{1}_{q}⊘\boldsymbol{\Gamma}=e^{.-log\left( \boldsymbol{\Gamma} \right)}$, $log\left( \boldsymbol{\Gamma} \right)$ acts as an elementwise function. The logarithm can be expressed as an infinite Taylor series (equation VII-32).

$log\left( \boldsymbol{\Gamma} \right)=\sum_{\mathcal{l=}1}^{\infty} \frac{\left( -1 \right)^{\mathcal{l+}1}}{\mathcal{l}}\left( \boldsymbol{\Gamma}-\mathbf{1}_{q} \right)^{\mathcal{.l}}$

Without losing generality we can consider that the elements in $\boldsymbol{\Gamma}$ belong to the open interval $\boldsymbol{\Gamma}\left( i,j \right)\in\left( 0,1 \right),\forall i,j$. Given the assumption before, the odd terms in the Taylor series of $-log\left( \boldsymbol{\Gamma} \right)$ become positive, the series (equation VII-32) can be rearranged into (equation VII-33).

$-log\left( \boldsymbol{\Gamma} \right)=\sum_{\mathcal{l=}1}^{\infty} \frac{\left( \mathbf{1}_{q}-\boldsymbol{\Gamma} \right)^{\mathcal{.l}}}{\mathcal{l}}$

Finally, by substituting in the elementwise exponential function in (equation VII-33) we can express the Hadamard Scaling as the element wise product of exponentials (equation VII-34).

$\mathbf{1}_{q}⊘\boldsymbol{\Gamma}=\prod_{\mathcal{l=}1}^{\infty} .\boldsymbol{e}^{.\frac{\left( \mathbf{1}_{q}-\boldsymbol{\Gamma} \right)^{\mathcal{l}}}{\mathcal{l}}}$

Here (equation VII-34), given the positive definiteness of $\boldsymbol{\Gamma}$ and since $\boldsymbol{\Gamma}\left( i,j \right)\in\left( 0,1 \right),\forall i,j$ it holds that $\left( \mathbf{1}_{q}-\boldsymbol{\Gamma} \right)$ is positive definite. Also, the elementwise exponentiation (equation VII-35) and (equation VII-36), and element wise product operation (equation VII-37) keep the positive definiteness.

$\left( \mathbf{1}_{q}-\boldsymbol{\Gamma} \right)^{\mathcal{.l}}$

$\boldsymbol{e}^{.\frac{\left( \mathbf{1}_{q}-\boldsymbol{\Gamma} \right)^{\mathcal{l}}}{\mathcal{l}}}$

$\prod_{\mathcal{l=}1}^{\infty} \cdot$

Thus, it is clear that ${\tilde{\boldsymbol{\Sigma}}}^{-1}=\boldsymbol{\Sigma}^{-1}⊘\boldsymbol{\Gamma}$ is positive definite so also it is $\tilde{\boldsymbol{\Sigma}}$.$∎$

**Remark:** By Lemma 4 we build a statistically equivalent model of the random matrix $\boldsymbol{\Psi}$, the de-standardization $\boldsymbol{\Phi}$ defined through the from the standard sampled covariance matrix $\tilde{\boldsymbol{\Psi}}$, in the sense that it keeps the same multivariate structure through the Wishart probability density function, and its inverse $\boldsymbol{\Phi}^{-1}$ keeps the same stochastic properties than $\boldsymbol{\Psi}^{-1}$, i.e. all their elements are independent with identical marginal probability density function [7]. This result prescribes a statistical equivalence between a model of the sampled covariance matrix $\boldsymbol{\Psi}$ defined by a Wishart probability density function, of $T$ degrees of freedom and positive definite scale matrix $\left( T\boldsymbol{\Theta} \right)^{-1}$, with precision matrix $\boldsymbol{\Theta}$, and model of the standard sampled covariance matrix $\tilde{\boldsymbol{\Psi}}$ defined by a Wishart probability density function (equation VII-38) and (equation VII-39), of $T$ degrees of freedom and positive definite scale matrix $\left( T\tilde{\boldsymbol{\Theta}} \right)^{-1}$, with standard Precision matrix $\tilde{\boldsymbol{\Theta}}$ given.

$p\left( \left. \tilde{\boldsymbol{\Psi}} \right|\tilde{\boldsymbol{\Theta}} \right)=W_{q}^{\mathbb{C}}\left( \tilde{\boldsymbol{\Psi}} | \left( T\tilde{\boldsymbol{\Theta}} \right)^{-1},T \right)$

$W_{q}^{\mathbb{C}}\left( \tilde{\boldsymbol{\Psi}} | \left( T\tilde{\boldsymbol{\Theta}} \right)^{-1},T \right)\propto\left| \tilde{\boldsymbol{\Psi}} \right|^{T-q}\left| \tilde{\boldsymbol{\Theta}} \right|^{T}\boldsymbol{e}^{\boldsymbol{-}Ttr\left( \tilde{\boldsymbol{\Theta}}\tilde{\boldsymbol{\Psi}} \right)}$

This statistical standardization is also consistent when the sample number $T$ tends to infinite, given the inverse sampled covariance matrix tendency in probability to $\boldsymbol{\Theta}$, i.e. $P\left( \boldsymbol{\Psi}^{-\mathbf{1}}\in\mathcal{B}\left( \boldsymbol{\Theta} \right) \right)\to1$ as $T\to\infty$ for any open set $\mathcal{B}\left( \boldsymbol{\Theta} \right)$ containing $\boldsymbol{\Theta}$ in the $q^{2}$-dimensional complex-valued Euclidean space. A natural estimator of the standard precision matrix $\tilde{\boldsymbol{\Theta}}$ can be computed by maximum Likelihood through direct differentiation of (equation VII-38) and (equation VII-39) $\hat{\tilde{\boldsymbol{\Theta}}}={\tilde{\boldsymbol{\Psi}}}^{-\mathbf{1}}$, then, in agreement with the conditions and equivalence shown in Lemma 4 it holds that (equation VII-40).

$\hat{\tilde{\boldsymbol{\Theta}}}=\boldsymbol{\Psi}⊘\boldsymbol{\Gamma}$

Therefore, given the tendency in probability of $\boldsymbol{\Psi}^{-\mathbf{1}}$ it can also be directly deduced the tendency in probability of the standard precision matrix estimator, i.e. $P\left( \hat{\tilde{\boldsymbol{\Theta}}}\in\mathcal{B}\left( \boldsymbol{\Theta}⊘\boldsymbol{\Gamma} \right) \right)\to1$ as $T\to\infty$ for any open set $\mathcal{B}\left( \boldsymbol{\Theta}⊘\boldsymbol{\Gamma} \right)$ containing $\boldsymbol{\Theta}⊘\boldsymbol{\Gamma}$ in the $q^{2}$-dimensional complex Euclidean space. $∎$

## hermitian graphical Ridge (hgRidge) and hgLASSO LQA estimator (Lemma 5)

***Lemma 5 (hermitian graphical Ridge estimator):*** *Given the local quadratic approximation of Lemma 3 and standardization of Lemma 4 the hermitian graphical LASSO admits a Ridge local representation: the pair given by the Likelihood (equation VIII-1) and prior (equation VIII-2).*

$p\left( \left. \tilde{\boldsymbol{\Psi}} \right|\tilde{\boldsymbol{\Theta}} \right)=W_{q}^{\mathbb{C}}\left( \tilde{\boldsymbol{\Psi}} | T^{-1}{\tilde{\boldsymbol{\Theta}}}^{-1},T \right)$

$p\left( \tilde{\boldsymbol{\Theta}} \right)=\exp\left( \left\| \tilde{\boldsymbol{\Theta}} \right\|_{2}^{2} | T/2 \right)$

*Furthermore, the hermitian graphical Ridge estimator, that maximizes the posterior distribution due to the pair (equation VIII-1) and (equation VIII-2) is the solution of the Riccati matrix equation (equation VIII-3).*

${\tilde{\boldsymbol{\Theta}}}^{2}+\tilde{\boldsymbol{\Psi}}\tilde{\boldsymbol{\Theta}}-\mathbf{I}_{q}=\mathbf{0}_{q}$

*There is the unique positive definite and hermitic solution to (equation VIII-3) that also shares the eigenspace with* $\tilde{\boldsymbol{\Psi}}$ *expressed by the matrix square root formula (equation VIII-4).*

$\hat{\tilde{\boldsymbol{\Theta}}}=-\frac{1}{2}\tilde{\boldsymbol{\Psi}}+\frac{1}{2}\sqrt{{\tilde{\boldsymbol{\Psi}}}^{2}+4\mathbf{I}_{q}}$

***Proof of Lemma 5***

We base on a model with Wishart likelihood for the sampled covariance matrix $\boldsymbol{\Psi}$ (equation VIII-5) and the local quadratic approximation of Lemma 3 for the hermitian graphical LASSO prior upon the precision matrix $\boldsymbol{\Theta}$ (equation VIII-6).

$p\left( \left. \boldsymbol{\Psi} \right|\boldsymbol{\Theta} \right)=W_{q}^{\mathbb{C}}\left( \boldsymbol{\Psi} | T^{-1}\boldsymbol{\Theta}^{-1},T \right)$

$p\left( \boldsymbol{\Theta} \right)=\exp\left( \left\| \boldsymbol{\Theta}⊘\boldsymbol{\Gamma} \right\|_{2}^{2} | T/2 \right)$

We demonstrated the statistical equivalence between this model (equation VIII-5) and (equation VIII-6) and standard formulation of Lemma 4 for the Wishart likelihood upon $\tilde{\boldsymbol{\Psi}}=\left( \boldsymbol{\Psi}^{-1}⊘\boldsymbol{\Gamma} \right)^{-1}$ (equation VIII-7) with hermitian graphical Ridge prior upon $\tilde{\boldsymbol{\Theta}}=\boldsymbol{\Theta}⊘\boldsymbol{\Gamma}$ (equation VIII-8).

$p\left( \left. \tilde{\boldsymbol{\Psi}} \right|\tilde{\boldsymbol{\Theta}} \right)=W_{q}^{\mathbb{C}}\left( \tilde{\boldsymbol{\Psi}} | T^{-1}{\tilde{\boldsymbol{\Theta}}}^{-1},T \right)$

$p\left( \tilde{\boldsymbol{\Theta}} \right)=\exp\left( \left\| \tilde{\boldsymbol{\Theta}} \right\|_{2}^{2} | T/2 \right)$

The posterior distribution of based on the Bayes theorem for the likelihood (equation VIII-7) and prior (equation VIII-8) is the given by (equation VIII-9).

$p\left( \left. \tilde{\boldsymbol{\Theta}} \right|\tilde{\boldsymbol{\Psi}} \right)=\left| \tilde{\boldsymbol{\Theta}} \right|^{T}\boldsymbol{e}^{\boldsymbol{-}Ttr\left( \tilde{\boldsymbol{\Theta}}\tilde{\boldsymbol{\Psi}} \right)}e^{-\frac{T}{2}\left\| \tilde{\boldsymbol{\Theta}} \right\|_{2}^{2}}$

Applying the $-log$ transformation to this posterior distribution (equation VIII-9) we obtain the standard target function $\mathcal{L}\left( \tilde{\boldsymbol{\Theta}} \right)$ and estimator $\hat{\tilde{\boldsymbol{\Theta}}}$ (equation VIII-10).

$\begin{matrix} \mathcal{L}\left( \tilde{\boldsymbol{\Theta}} \right)=-T\log\left| \tilde{\boldsymbol{\Theta}} \right|+Ttr\left( \tilde{\boldsymbol{\Theta}}\tilde{\boldsymbol{\Psi}} \right)+\frac{T}{2}\left\| \tilde{\boldsymbol{\Theta}} \right\|_{2}^{2} \\ \hat{\tilde{\boldsymbol{\Theta}}}={argmin}_{\tilde{\boldsymbol{\Theta}}}\left\{ -\log\left| \tilde{\boldsymbol{\Theta}} \right|+tr\left( \tilde{\boldsymbol{\Theta}}\tilde{\boldsymbol{\Psi}} \right)+\frac{1}{2}\left\| \tilde{\boldsymbol{\Theta}} \right\|_{2}^{2} \right\} \end{matrix}$

Since this target function is convex and differentiable the necessary and sufficient condition for the minimum given by the zero of the derivative ${\partial\mathcal{L}\left( \tilde{\boldsymbol{\Theta}} \right)}/{\partial\tilde{\boldsymbol{\Theta}}}=\boldsymbol{0}_{q}$ represented by the following matrix equation (equation VIII-11) with solution for the zero $\hat{\tilde{\boldsymbol{\Theta}}}$ given by the matrix squared root formula (equation VIII-12).

$-{\tilde{\boldsymbol{\Theta}}}^{-1}+\tilde{\boldsymbol{\Psi}}+\tilde{\boldsymbol{\Theta}}=\boldsymbol{0}_{q}$

$\hat{\tilde{\boldsymbol{\Theta}}}=-\frac{1}{2}\tilde{\boldsymbol{\Psi}}+\frac{1}{2}\sqrt{{\tilde{\boldsymbol{\Psi}}}^{2}+4\mathbf{I}_{q}}$

**Remark:** The expression (equation VIII-11) constitutes a special case of the Riccati matrix equation [8]. Positive definiteness and hermiticity of its solution $\hat{\tilde{\boldsymbol{\Theta}}}$, as a natural property of this estimator, is required. Also, it can be checked that the solution $\hat{\tilde{\boldsymbol{\Theta}}}$ (equation VIII-12) must share the same eigenspace with $\tilde{\boldsymbol{\Psi}}$, since from (equation VIII-11) two auxiliary second order matrix equations hold, i.e. ${\tilde{\boldsymbol{\Theta}}}^{2}+\tilde{\boldsymbol{\Theta}}\tilde{\boldsymbol{\Psi}}-\mathbf{I}_{q}=\mathbf{0}$ and ${\tilde{\boldsymbol{\Theta}}}^{2}+\tilde{\boldsymbol{\Psi}}\tilde{\boldsymbol{\Theta}}-\mathbf{I}_{q}=\mathbf{0}_{q}$, given the left and right multiplication by the Standard Precision matrix. This imply that any solution $\hat{\tilde{\boldsymbol{\Theta}}}$ commute with $\tilde{\boldsymbol{\Psi}}$, and thus they share the eigenspace.$∎$

Let’s show first that the proposed solution is positive definite. Given the positive definiteness and hermiticity of the complex-valued matrix $\tilde{\boldsymbol{\Psi}}$ it admits a singular value decomposition $\tilde{\boldsymbol{\Psi}}=\tilde{\mathbf{U}}\tilde{\mathbf{D}}{\tilde{\mathbf{U}}}^{\dagger}$ with real and positive singular values $\tilde{\mathbf{D}}$. Thus, the argument in the matrix square root of formula (equation VIII-12) admits a singular value decomposition of the kind ${\tilde{\boldsymbol{\Psi}}}^{2}+4\mathbf{I}_{q}=\tilde{\mathbf{U}}\left( {\tilde{\mathbf{D}}}^{\mathbf{2}}+4\mathbf{I}_{q} \right){\tilde{\mathbf{U}}}^{\dagger}$, where its singular values, given by ${\tilde{\mathbf{D}}}^{\mathbf{2}}+4\mathbf{I}_{q}$, are also real and positive. In consequence the square root term (equation VIII-12) has also real and positive singular values given by the following singular value decomposition:

$\sqrt{{\tilde{\boldsymbol{\Psi}}}^{2}+4\mathbf{I}_{q}}=\tilde{\mathbf{U}}\sqrt{{\tilde{\mathbf{D}}}^{\mathbf{2}}+4\mathbf{I}_{q}}{\tilde{\mathbf{U}}}^{\dagger}$

Finally, the singular value decomposition of the standard precision matrix estimator $\hat{\tilde{\boldsymbol{\Theta}}}$ (equation VIII-12) can be expressed as (equation VIII-14).

$\hat{\tilde{\boldsymbol{\Theta}}}=\tilde{\mathbf{U}}\left( \frac{1}{2}\sqrt{{\tilde{\mathbf{D}}}^{\mathbf{2}}+4\mathbf{I}_{q}}-\frac{1}{2}\boldsymbol{I}_{q} \right){\tilde{\mathbf{U}}}^{\dagger}$

It is clear, by formula (equation VIII-14), that the singular values of the standard precision matrix estimator are real and positive numbers. The Standard Precision matrix estimator commutes with the standard sampled covariance matrix $\tilde{\boldsymbol{\Psi}}$ since they share the eigenspace, i.e. $\tilde{\boldsymbol{\Psi}}\hat{\tilde{\boldsymbol{\Theta}}}=\hat{\tilde{\boldsymbol{\Theta}}}\tilde{\boldsymbol{\Psi}}$. Given the singular value decomposition in equation (equation VIII-14) we can get that the singular value decomposition of $\tilde{\boldsymbol{\Psi}}\hat{\tilde{\boldsymbol{\Theta}}}$ can be expressed as (equation VIII-15).

$\tilde{\boldsymbol{\Psi}}\hat{\tilde{\boldsymbol{\Theta}}}=\tilde{\mathbf{U}}\left( \tilde{\mathbf{D}}\left( \frac{1}{2}\sqrt{{\tilde{\mathbf{D}}}^{2}+4\mathbf{I}_{q}}-\frac{1}{2}\mathbf{I}_{q} \right) \right){\tilde{\mathbf{U}}}^{\dagger}$

The product of diagonal matrices always commutes, so we can directly check that (equation VIII-15) is also the singular value decomposition of $\hat{\tilde{\boldsymbol{\Theta}}}\tilde{\boldsymbol{\Psi}}$. To check that the proposed estimator (equation VIII-12) satisfies the equation (equation VIII-11) it is enough to check that it also satisfies the pair of equations $\hat{\tilde{\boldsymbol{\Theta}}}\tilde{\boldsymbol{\Psi}}=\mathbf{I}_{q}-{\hat{\tilde{\boldsymbol{\Theta}}}}^{2}$ and $\tilde{\boldsymbol{\Psi}}\hat{\tilde{\boldsymbol{\Theta}}}=\mathbf{I}_{q}-{\hat{\tilde{\boldsymbol{\Theta}}}}^{2}$. The left side in both equations are equal, i.e. $\hat{\tilde{\boldsymbol{\Theta}}}\tilde{\boldsymbol{\Psi}}=\tilde{\boldsymbol{\Psi}}\hat{\tilde{\boldsymbol{\Theta}}}$, and given by formula (equation VIII-15), so, lets evaluate the right side.

$\mathbf{I}_{q}-{\hat{\tilde{\boldsymbol{\Theta}}}}^{2}=\mathbf{I}_{q}-\left( -\frac{1}{2}\tilde{\boldsymbol{\Psi}}+\frac{1}{2}\sqrt{{\tilde{\boldsymbol{\Psi}}}^{2}+4\lambda\mathbf{I}_{q}} \right)^{2}$

Effectuating the matrix square operation in (equation VIII-16) we obtain (equation VIII-17).

$\mathbf{I}_{q}-{\hat{\tilde{\boldsymbol{\Theta}}}}^{2}=\mathbf{I}_{q}-\left( \frac{1}{2} \right)^{\mathbf{2}}\left( {\tilde{\boldsymbol{\Psi}}}^{2}-\tilde{\boldsymbol{\Psi}}\sqrt{{\tilde{\boldsymbol{\Psi}}}^{\mathbf{2}}+4\mathbf{I}_{q}}-\sqrt{{\tilde{\boldsymbol{\Psi}}}^{\mathbf{2}}+4\mathbf{I}_{q}} \tilde{\boldsymbol{\Psi}}+{\tilde{\boldsymbol{\Psi}}}^{2}+4\mathbf{I}_{q} \right)$

From the singular value decomposition analysis above the matrices sampled covariance matrix $\tilde{\boldsymbol{\Psi}}$ and the square root term in (equation VIII-13) share the same eigenspace and thus commute, so, rearranging (equation VIII-17) it can be obtained (equation VIII-18).

$\mathbf{I}_{q}-\lambda{\hat{\tilde{\boldsymbol{\Theta}}}}^{2}=\mathbf{I}_{q}-2\left( \frac{1}{2} \right)^{\mathbf{2}}{\tilde{\boldsymbol{\Psi}}}^{2}+2\left( \frac{1}{2} \right)^{\mathbf{2}}\tilde{\boldsymbol{\Psi}}\sqrt{{\tilde{\boldsymbol{\Psi}}}^{\mathbf{2}}+4\mathbf{I}_{q}}-4\left( \frac{1}{2} \right)^{\mathbf{2}}\mathbf{I}_{q}$

From (equation VIII-18) and considering (equation VIII-17) it is direct that following identity holds (equation VIII-19).

$\mathbf{I}_{q}-{\hat{\tilde{\boldsymbol{\Theta}}}}^{2}=-\frac{1}{2}{\tilde{\boldsymbol{\Psi}}}^{2}+\frac{1}{2}\tilde{\boldsymbol{\Psi}}\sqrt{{\tilde{\boldsymbol{\Psi}}}^{\mathbf{2}}+4\mathbf{I}_{q}}=\tilde{\boldsymbol{\Psi}}\hat{\tilde{\boldsymbol{\Theta}}}$

Now we need to proof that the proposed estimator (equation VIII-12) is the unique solution of equation (equation VIII-11) that commute with $\tilde{\boldsymbol{\Psi}}$. Let $\hat{\tilde{\boldsymbol{\Phi}}}$ be another solution that also commutes with $\tilde{\boldsymbol{\Psi}}$. Since the matrix $\hat{\tilde{\boldsymbol{\Phi}}}$ commutes with $\tilde{\boldsymbol{\Psi}}$ it has the same eigenspace, i.e. it admits a singular value decomposition of the kind $\hat{\tilde{\boldsymbol{\Phi}}}=\tilde{\mathbf{U}}\tilde{\mathbf{E}}{\tilde{\mathbf{U}}}^{\dagger}$. Also, since $\hat{\tilde{\boldsymbol{\Phi}}}$ satisfies equation (equation VIII-11) it can be checked that $-{\tilde{\mathbf{E}}}^{-1}+\tilde{\mathbf{D}}+\tilde{\mathbf{E}}=\mathbf{0}_{q}$. The solution of this equation is straightforward given diagonal matrices $\tilde{\mathbf{E}}$ and $\tilde{\mathbf{D}}$ (equation VIII-20).

$\tilde{\mathbf{E}}=\frac{1}{2}\sqrt{{\tilde{\mathbf{D}}}^{\mathbf{2}}+4\mathbf{I}_{q}}-\frac{1}{2}\mathbf{I}_{q}$

This (equation VIII-20) therefore yields that $\hat{\tilde{\boldsymbol{\Phi}}}=\hat{\tilde{\boldsymbol{\Theta}}}$ since they have identical eigenspace and eigenvalues.$∎$

# References

[1] A. P. Dempster, N. M. Laird, and D. B. Rubin, “Maximum likelihood from incomplete data via the EM algorithm,” *J. R. Stat. Soc. Ser. B*, vol. 39, no. 1, pp. 1–22, 1977.

[2] C. Liu and D. B. Rubin, “The ECME algorithm: a simple extension of EM and ECM with faster monotone convergence,” *Biometrika*, vol. 81, no. 4, pp. 633–648, 1994.

[3] G. J. McLachlan and T. Krishnan, *The EM algorithm and extensions*, vol. 382. John Wiley & Sons, 2007.

[4] A. Wills, B. Ninness, and S. Gibson, “Maximum likelihood estimation of state space models from frequency domain data,” *IEEE Trans. Automat. Contr.*, vol. 54, no. 1, pp. 19–33, 2009.

[5] Andrews, “Scale Mixtures of Normal Distributions,” *J. R. Stat. Soc. Ser. B*, vol. 148, pp. 148–162, 1974, doi: https://www.jstor.org/stable/2984774.

[6] J. Schur, “Bemerkungen zur Theorie der beschränkten Bilinearformen mit unendlich vielen Veränderlichen.,” 1911.

[7] M. Drton, H. Massam, and I. Olkin, “Moments of minors of Wishart matrices,” *Ann. Stat.*, vol. 36, no. 5, pp. 2261–2283, 2008.

[8] J. Honorio and T. Jaakkola, “Inverse Covariance Estimation for High-Dimensional Data in Linear Time and Space: Spectral Methods for Riccati and Sparse Models,” *arXiv Prepr. arXiv1309.6838*, pp. 1329–1336, 2013, [Online]. Available: http://arxiv.org/abs/1309.6838
